# Candidate Chemosensory Genes Identified in the Adult Antennae of *Sympiezomias velatus* and Binding Property of Odorant-Binding Protein 15

**DOI:** 10.3389/fphys.2022.907667

**Published:** 2022-05-31

**Authors:** Xiao Li, Jian-Wen Li, Wen-Xiu Sun, Wei Li, Hua-Yuan Gao, Tong-Xian Liu, Ming-Jing Qu

**Affiliations:** ^1^ Shandong Peanut Research Institute, Qingdao, China; ^2^ College of Life Sciences, Yangtze University, Jingzhou, China; ^3^ Weinan Product Quality Supervision and Inspection Institute, Weinan, China; ^4^ Peanut Research Institute, Jilin Academy of Agricultural Sciences, Changchun, China; ^5^ College of Plant Health and Medicine, Qingdao Agricultural University, Qingdao, China

**Keywords:** *Sympiezomias velatus*, chemosensory genes, transcriptome analysis, expression pattern, odorant-binding protein, fluorescence competition binding assay

## Abstract

Chemosensory genes play important roles in insect behaviors and have thus become potential molecular targets for pest control based on the manipulation of chemoreception-driven behaviors. The great gray weevil *Sympiezomias velatus* (Chevrolat) (Coleoptera: Curculionidae) is an important agricultural pest that causes serious economic losses to many crops in China, but its chemosensory genes have not been reported. Here we assembled the antennal transcriptomes of female and male adult *S. velatus* and revealed the major chemosensory genes necessary for olfaction. A total of 138 candidate chemosensory genes in six families were identified, including 41 encoding odorant-binding proteins (OBPs), 11 encoding chemosensory proteins (CSPs), 62 encoding odorant receptors (ORs), 15 encoding gustatory receptors (GRs), six encoding ionotropic receptors (IRs), and three encoding sensory neuron membrane proteins (SNMPs). We analyzed their phylogenetic relationship based on the amino acid sequences of these chemosensory-related protein families in *S. velatus* and other insects, and the expression profiles based on their antennal transcriptomes. Chemosensory genes that show antenna-abundant/specific or sex-biased expression were observed, suggesting that these genes might have functions in olfaction. Furthermore, we chose an antenna-abundant OBP belonging to ABPX subfamily, SvelOBP15, to investigate its binding property. The results showed that among 33 tested compounds, SvelOBP15 displayed high binding affinities (Ki = 7.36–12.94 μmol/L) with farnesol, nerolidol, limonene and diisobutyl phthalate, indicating that SvelOBP15 plays olfactory roles by binding and transporting specific plant volatiles. These findings will help us better understand the olfactory systems of *S. velatus*, and provide a basis for functional elucidation of these chemosensory genes.

## Introduction

The olfactory system plays a crucial role in insect behaviors, such as foraging, mating, oviposition selection, spawning and avoiding predators (Sanchez-Gracia et al., 2009; Fleischer et al., 2018). In this sophisticated and senstive system, the antenna is the primary olfactory organ with numerous sensilla distributed on its surface. Olfactory sensory neurons (OSNs) housed within the olfactory sensilla play a key role in olfactory signal transduction (i.e., transforming odor cues into neuronal electrical signals) (Schmidt and Benton, 2020). Several chemosensory gene families have been demonstrated to be involved in odor perception. These include three divergent chemoreceptors [i.e., odorant receptors (ORs), ionotropic receptors (IRs) and gustatory receptors (GRs)] and nonreceptor families [i.e., odorant-binding proteins (OBPs), chemosensory proteins (CSPs) and sensory neuron membrane proteins (SNMPs)]. More specifically, odorant molecules enter the sensillar lymph through pores on the surface of sensilla, are recognized and bound by OBPs or CSPs in the lymph and transported to ORs, GRs or IRs expressed on the dendritic membranes of OSNs (Leal, 2013; Fleischer et al., 2018; Schmidt and Benton, 2020). The chemoreceptors are then activated, the electrical signals generated are conveyed to the antennal lobe, and the neuronal signal is ultimately decoded by the insect brain (Fleischer et al., 2018).

OBPs are small water-soluble proteins with hydrophobic cavity which allow them to recognize and bind hydrophobic chemicals (Vogt and Riddiford, 1981; Leal, 2013). It is generally believed that OBPs are categorized into three different subgroups according to the number of conserved cysteine residues. Classic OBPs contain six highly conserved cysteine residues that form three disulfide bridges (C1–C3, C2–C5, and C4–C6) (Leal et al., 1999). Minus-C OBPs derived from classic OBPs lack C2–C5 disulfide bonds, and this might present a more flexible substrate-binding site and a broader binding spectrum of odorant molecules (Hekmat-Scafe et al., 2002). Plus-C OBPs have additional cysteine residues and proline residues based on classic OBPs (Zhou et al., 2004). In Lepidoptera, OBPs are mainly divided into three subfamilies, including general odorant-binding proteins (GOBPs), pheromone binding proteins (PBPs), and antennal-binding proteins (ABPs) (Vogt and Riddiford, 1981; Vogt et al., 1991; Krieger et al., 1996). Earlier studies revealed that GOBPs primarily bind to common odor molecules (Vogt et al., 2002) while PBPs and ABPs mainly bind to insect sex pheromones (Vogt and Riddiford, 1981; Vogt et al., 1991; Zhou et al., 2009). However, further studies revealed that these OBP subfamilies have more broad spectra of odor-binding activity, i.e., PBPs were reported to bind to some plant odorants and GOBPs also showed high binding affinities to sex pheromone component in some cases (Zhou et al., 2009; Liu et al., 2012; Liu et al., 2015).

The great gray weevil, *Sympiezomias velatus* Chevrolat (Coleoptera, Curculionidae), is a polyphagous agricultural pest that attacks many crop seedlings such as peanut, soybean, cotton and corn, as well as many fruit trees and forest trees such as apple, pear, poplar and elm (Tang et al., 2017; Li et al., 2018). This pest is widely prevalent in China and causes great economic losses. At present, the control of *S. velatus* mainly depends on chemical insecticides. Pesticide residue problems and environmental contamination caused by the abuse of agents are growing. Therefore, development of novel, effective and environmentally friendly strategies to control this pest is urgently needed. The olfactory proteins n insects, such as OBPs and ORs, represent potential molecular targets that can be used to discover novel potent semiochemicals for environmental-friendly approaches in pest management (Venthur and Zhou, 2018). In recent years, an increasing number of olfactory-sensing genes in coleopterans have been identified based on the development of high-throughput sequencing technology including a few weevil species (including but not limited to *Rhynchophorus ferrugineus*, *Dendroctonus ponderosae*, *Cylas formicarius*, *Lissorhoptrus oryzophilus*, *Eucryptorrhynchus scrobiculatus* and *E. brandti*) (Andersson et al., 2013; Antony et al., 2016; Yuan et al., 2016; Bin et al., 2017; Wen et al., 2018; Andersson et al., 2019; Zhang Y. et al., 2019). However, little is known regarding any chemosensory genes in *S. velatus.*

In the present study, olfaction-related genes were identified from *S. velatus* based on antennal transcriptome sequencing. The structures and characteristics of candidate genes were then analyzed using bioinformatics methods. In addition, gene expression profiling was performed to identify differentially expressed genes (DEGs). Based on the above, one ABP, SvelOBP15, was found to be expressed particularly highly at the transcriptional level in the antennae. To explore the binding characteristics of this protein, we attempted to express and purify it, and screen for active odorant compounds associated with the ecological significance of the weevil. We aimed to provide molecular background for future functional characterization in order to better understand the olfactory system of *S. velatus*.

## Materials and Methods

### Insect Rearing and Tissue Collection

Mass adult *S. velatus* were originally collected from a peanut field in Fuyu City, Jilin Province, China (45.11°N, 125.73°E) in the summer of 2016. They were reared on peanut seedlings in rearing cages in our laboratory at 26 ± 1°C and 60 ± 5% relative humidity under a photoperiod of 16 h light: 8 h dark. The insects were collected and separated by sex. Their antennae were then cut at the base of the pedicel using a forcep, and transferred immediately to a precooled Eppendorf tube in liquid nitrogen. The frozen antennae were stored at −80°C. Approximately 300 pairs of antennae for each sex were used for transcriptome sequencing, and three biological replicates were included in this experiment.

### RNA Extraction and Illumina Sequencing

Total RNA was extracted separately from the female and male samples using the RNAiso Plus RNA Extraction Kit (TaKaRa, Tokyo, Japan) according to the manufacturer’s protocol. A 2100 RNA Nano 6000 Assay Kit (Agilent Technologies, Santa Clara, United States) was utilized to assess the concentration and integrity of the RNA samples, and 1% agarose gel electrophoresis was used to detect the degradation and contamination of total RNA.

The cDNA libraries were constructed using reagents provided in the Illumina sequencing kit, according to the manufacturer’s recommendations. Finally, the libraries were sequenced using an Illumina HiSeq 2000 platform (Annoroad Gene Technology, Beijing, China). The raw transcriptome data have been submitted to the NCBI Sequence Read Archive (SRA) with accession numbers SSR17542735-SSR17542740.

### *De Novo* Assembly and Sequence Annotation

After the raw reads were filtered to remove adaptors, low-quality sequences and reads with an N content greater than 5%, the high-quality clean reads were assembled into unigenes using Trinity v2.8.4 (Grabherr et al., 2011). The annotation of unigene sequences was then performed against the NCBI non-redundant protein sequence (Nr), non-redundant nucleotide sequence (Nt) and universal protein resource (UniProt) databases using Trinotate v3.1.1 with a cutoff E-value of 10^−5^. tBlastn was utilized to search for putative *S. velatus* chemosensory genes using the known homologous sequences from other insect species as queries (with a cutoff E-value of 10^−5^). All putative chemosensory genes were in turn checked using NCBI’s Blastx program. Then all these assembled transcripts were verified using reverse transcription PCR and Sanger sequencing. The open reading frames (ORFs) of the candidate chemosensory genes were predicted using the online tool ORF finder (http://www.ncbi.nlm.nih.gov/gorf/gorf.html) and manually corrected by comparing the predicted sequences to the NCBI Nr protein database using Blastp (https://blast.ncbi.nlm.nih.gov/Blast.cgi). All these protein sequences were further checked for the presence of the conserved domains using the Pfam web server (http://pfam.xfam.org) (Mistry et al., 2021). The signal peptides of OBPs and CSPs were predicted using SignalP 4.1 (http://www.cbs.dtu.dk/services/SignalP/) (Petersen et al., 2011). The transmembrane domains (TMDs) of ORs, GRs, IRs and SNMPs were predicted using TMHMM 2.0 (http://www.cbs.dtu.dk/services/TMHMM). The theoretical pI (isoelectric point) and Mw (molecular weight) of SvelOBP15 were predicted using the Compute pI/Mw tool (http://web.expasy.org/compute_pi/).

### Phylogenetic Analysis

Multiple sequence alignment was performed using MAFFT v7.157 (Katoh and Standley, 2013). Ambiguously aligned regions were removed using Gblocks v0.91b (Castresana, 2000; Talavera and Castresana, 2007). Then, maximum-likelihood phylogenetic trees were constructed with FastTree v2.1.7 (Price et al., 2010) under the default settings based on the alignment. The analysis included putative chemosensory-related protein sequences of *S. velatus* (included in Supplementary Material S1), *Tribolium castaneum* (Richards et al., 2008), *D. ponderosae* (Andersson et al., 2019), *R. ferrugineus* (Antony et al., 2016), *Colaphellus bowringi* (Li X.-M. et al., 2015), *Anomala corpulenta* (Li X. et al., 2015), *Ips typographus* (Andersson et al., 2013), *Agrilus planipennis* (Andersson et al., 2019), *C. formicarius* (Bin et al., 2017), *Megacyllene caryae* (Mitchell et al., 2012), *Phyllotreta striolata* (Hull et al., 2016), *D. melanogaster* (Hekmat-Scafe et al., 2002), *Anopheles gambiae* (Vieira and Rozas, 2011) or *Bombyx mori* (Gong et al., 2009). Signal peptide sequences were removed from the OBP and CSP datasets prior to the analyses because these regions have a high substitution rate. Incomplete sequences with a length shorter than 150 amino acids (apart from the OBPs and CSPs) were excluded from the datasets used for phylogenetic analyses. Additionally, a neighbor-joining (NJ) tree of OBPs was also constructed based on the amino acid sequences of four weevil species (*S. velatus*, *R. ferrugineus*, *D. ponderosae* and *Sitophilus zeamais*) (Andersson et al., 2013; Antony et al., 2016; Andersson et al., 2019; Tang et al., 2019) and four lepidopteran species (*B. mori*, *Helicoverpa armigera*, *Spodoptera exigua* and *Athetis lepigone*) (Gong et al., 2009; Dani et al., 2011; Zhang et al., 2011; Zhang et al., 2017; Du et al., 2018). Multiple sequence alignment of OBP sequences without signal peptides was performed using ClustalX 2.0 (Larkin et al., 2007). The tree was constructed using MEGA 7.0 with the Jones-Taylor-Thornton (JTT) substitution model, pairwise deletion of gaps and 1000 bootstrap replicates (Kumar et al., 2016). All phylogenetic trees were finally visualized and colored using FigTree 1.4.3 (Rambaut, 2017).

### Sex-Specific Expression Profiles

Gene expression levels were measured in terms of fragments per kilobase per million mapped reads (FPKM), which were calculated based on the number of mapped sequence fragments corrected for transcript length and sequencing depth (Mortazavi et al., 2008). Analyses of DEGs between female and male antennae were performed using the DESeq2 package with |log_2_ratio| ≥ 1 and FDR <0.05 as the cutoff (Love et al., 2014). The log_10_ (FPKM + 1) values were used to establish a heat plot to represent the expression levels of the candidate chemoreception genes.

### Real-Time Quantitative PCR (RT-qPCR) Validation

RT-qPCR was used to validate the relative expression levels of the chemoreception genes. Total RNA was extracted separately from the female antennae, male antennae and male abdomen using the above-described method. cDNA was then synthesized using a PrimeScript™ RT reagent Kit with gDNA Eraser (Perfect Real Time) (Takara, Dalian, China). Primer Premier 5.0 software (Premier Biosoft, Palo Alto, CA, United States) was used to design the gene-specific primers for RT-qPCR (Supplementary Table S1). A single peak at the melting temperature and a single band in the 1% agarose gel electrophoresis confirmed primer specificity. The standand curve method was used to dertermine the amplification efficiency of each primer pair and those with nearly identical amplification efficiency were selected for gene expression analysis (Pfaffl, 2001).

RT-qPCR was run using SYBR Premix Ex Taq II (Tli RNaseH Plus) (Takara, Dalian, China) on an iQ™5 Multicolor Real-Time PCR Detection System (Bio-Rad Hercules, CA, United States). The reaction procedure was as follows: 95°C for 3 min; 40 cycles of 95°C for 10 s and 60°C for 20 s; and a melting cycle (from 60 to 95°C with an increase of 0.5°C per cycle). The experiment included three biological replicates and three technical replicates. The gene encoding 40 S ribosomal protein S20 (*RPS20*) was selected as the reference gene for normalization of the expression of the target genes (Li et al., 2018). The relative expression level of the target genes was calculated using the 2^−ΔΔCt^ method (Livak and Schmittgen, 2001). The statistical analyses were performed using SPSS 20.0 (SPSS Inc., Chicago, IL, United States), and the statistical significance was evaluated by one-way analysis of variance (ANOVA) followed by Tukey’s multiple comparisons at the 0.05 level.

### Recombinant Protein Expression and Purification

The whole ORF of *SvelOBP15* was successfully validated by RT-PCR and Sanger sequencing and then synthesized (without the signal peptide-encoding region) by Sangon Biotech (Shanghai, China). It was first cloned into the pGEM-T cloning vector (Promega, Madison, United States) and then subcloned into the expression vector pET-30a(+) (Novagen, Darmstadt, Germany). Recombinant SvelOBP15 was expressed in *Escherichia coli* Transetta (DE3) following induction with 0.5 mM isopropyl-b-D-thiogalactopyranoside (IPTG) after the culture reached an OD600 of 0.4–0.6. Incubation was continued at 37°C with shaking at 220 rpm for an additional 6 h. SDS-PAGE analysis revealed recombinant OBP15 as insoluble inclusion bodies. Denaturation and renaturation were performed following previously reported methods (Liu et al., 2012). The protein was purified using a HisTrap HP prepacked column (GE Healthcare, Pittsburgh, United States) according to the manufacturer’s instructions, and recombinant enterokinase (rEK) (GenScript Biology Company, Nanjing, China) was used to remove the His-tag. After running the digested protein back through the HisTrap HP prepacked column, the tag-free protein was obtained in the flow-through fraction. Proteins were visualized at all stages of purification by SDS-PAGE.

### Fluorescence Competitive Binding Assay

Fluorescence competitive binding assays were performed as reported previously (Liu et al., 2015). This assay was conducted on a Hitachi F-7000 fluorescence spectrophotometer with a 1-cm light path quartz cuvette and 10-nm slits for both excitation and emission. The excitation wavelength was 337 nm, and the emission spectrum was recorded between 390 and 460 nm. First, to determine the binding affinities of N-phenyl-1-naphthylamine (1-NPN) to OBP15, the fluorescence intensity values at maximum fluorescence emissions were plotted against the free ligand concentration by titrating increasing concentrations of 1-NPN. The binding curves were linearized using Scatchard plots. Next, competitive binding assays of OBP15 to 33 chemicals (Supplementary Table S2) of different structural characteristics were carried out using 1-NPN as the fluorescent reporter at a concentration of 2 μmol/L, whereas the concentration of each competitor varied from 2 to 20 μmol/L. These chemicals included the volatile components of host plants such as peanut, elm, maize, cotton, etc. (Hammack, 2001; Cardoza et al., 2002; Ngumbi et al., 2009; Cheng et al., 2010) and aggregation pheromone components of curculionids (Gries et al., 1993; Hallett et al., 1993; Eller et al., 1994; Szendrei et al., 2011). Chemicals were purchased from Sigma-Aldrich (St. Louis, United States) (purity >95% or 90%). Based on the assumption that the activity of proteins was 100% with a saturation stoichiometry of 1:1 (protein: ligand), the dissociation constants (Ki) of the competitors were calculated from the IC_50_ values (the concentrations of the ligands halving the fluorescence of 1-NPN) using the following equation:

Ki = [IC_50_]/(1 + [1-NPN]/K_1-NPN_) (Ban et al., 2003).Where [1-NPN] is the free concentration of 1-NPN and K_1-NPN_ is the dissociation constant of the complex protein/1-NPN.

Ki values of various compounds (mean ± SE) were subjected to one-way ANOVA followed by Tukey post hoc test for mean comparison using SPSS Statistics 20.0 (SPSS Inc., Chicago, United States).

## Results

### Transcriptome Assembly, Gene Annotation and Overview of Chemosensory Genes

Illumina sequencing of the *S. velatus* female and male antennal transcriptomes yielded a total of 44,429,256 and 41,778,454 clean reads, respectively. The combined transcriptome assembly using Trinity generated 160,467 transcripts; from these transcripts, 125,055 nonredundant unigenes with an N50 length of 828 bp were predicted, and 37,780 of these unigenes were annotated against the NCBI nr, nt, and UniProt databases using Trinotate software. A homology search of all unigenes against other insect species showed that the highest percentage of unigenes matched *D. ponderosae* (30.6%), followed by *T. castaneum* (21.4%). In total, we identified 138 chemosensory genes belonging to six gene families from the transcriptomic data of the *S. velatus* antennae, including 41 *OBP*s, 11 *CSP*s, 62 *OR*s, 15 *GR*s, six *IR*s and three *SNMP*s. A bioinformatics analysis predicted their ORFs, signal peptides (for OBPs and CSPs), number of conserved cysteine residues (for OBPs and CSPs), transmembrane domain (for ORs, GRs, IRs and SNMPs) and the best hits in the Nr database. All of this information is provided in Supplementary Tables S3, S4. Odorant-binding proteins.

**FIGURE 1:**
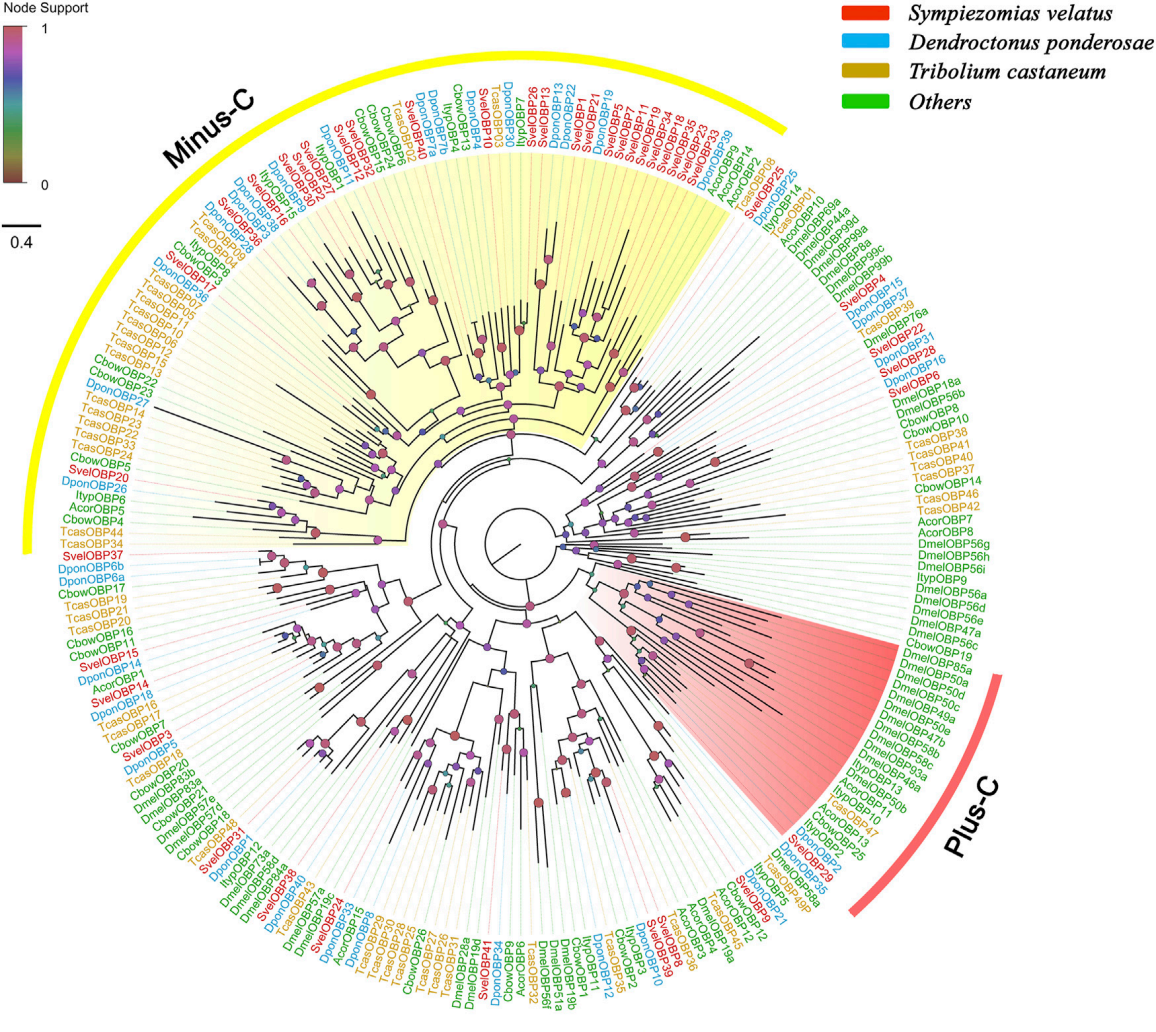
Phylogenetic tree of candidate odorant-binding proteins (OBPs). The OBP sequences included were from *Sympiezomias velatus* (Svel, red), *Dendroctonus ponderosae* (Dpon, blue), *Tribolium castaneum* (Tcas, yellow), and other species (green), including *Drosophila melanogaster* (Dmel), *Colaphellus bowringi* (Cbow), *Anomala corpulenta* (Acor) and *Ips typographus* (Ityp). The unrooted tree was constructed using FastTree based on a MAFFT alignment. The local support values based on the Shimodaira-Hasegawa (SH) test are indicated by the colored circles and increase with the brightness and size of the circles.

A total of 41 unigenes coding for SvelOBPs were identified in the transcriptome assembly, including 30 full-length unigenes and 11 partial unigenes. PBP/GOBP domain (Pfam ID: PF01395) is conserved in all SvelOBPs. Signal peptide prediction showed that among the full-length sequences, 17 SvelOBPs contained N-terminal signal peptide sequences (Supplementary Table S3). The pattern of conserved cysteine residues and the phylogenetic analysis revealed that 16 SvelOBPs belonged to Classic OBPs and 23 SvelOBPs were grouped into Minus-C OBPs. Only one Plus-C member (SvelOBP29) was found in *S. velatus* (Supplementary Table S3, Figure 1 and Supplementary Figures S1A–C), as was previously reported in other beetles, such as *T. castaneum*, *D. ponderosae* and *A. planipennis* (Dippel et al., 2014; Andersson et al., 2019). It is worth noting that we identified an unusually large OBP, SvelOBP10. Its unigene contained an incomplete ORF encoding a protein of 493 amino acids (Supplementary Table S3). Additionally, large species-specific expansion indicated by the branch of SvelOBP5/7/11/18/19/23/34/35 was also observed in *S. velatus* Minus-C OBPs, as reported with *T. castaneum* (Figure 1).

An NJ tree based on OBP sequences from four weevil and four moth species was also constructed, and the results showed that no candidate OBPs of weevil species fell into the well-supported lepidopteran PBP/GOBP clades, suggesting that they are not conserved between weevil species and lepidopteran moths. The ABPX subfamily was not clustered into one clade, and SvelOBP3, 14, 15, and 37 were located in branches with low support values (Supplementary Figure S2).

### Chemosensory Proteins

**FIGURE 2:**
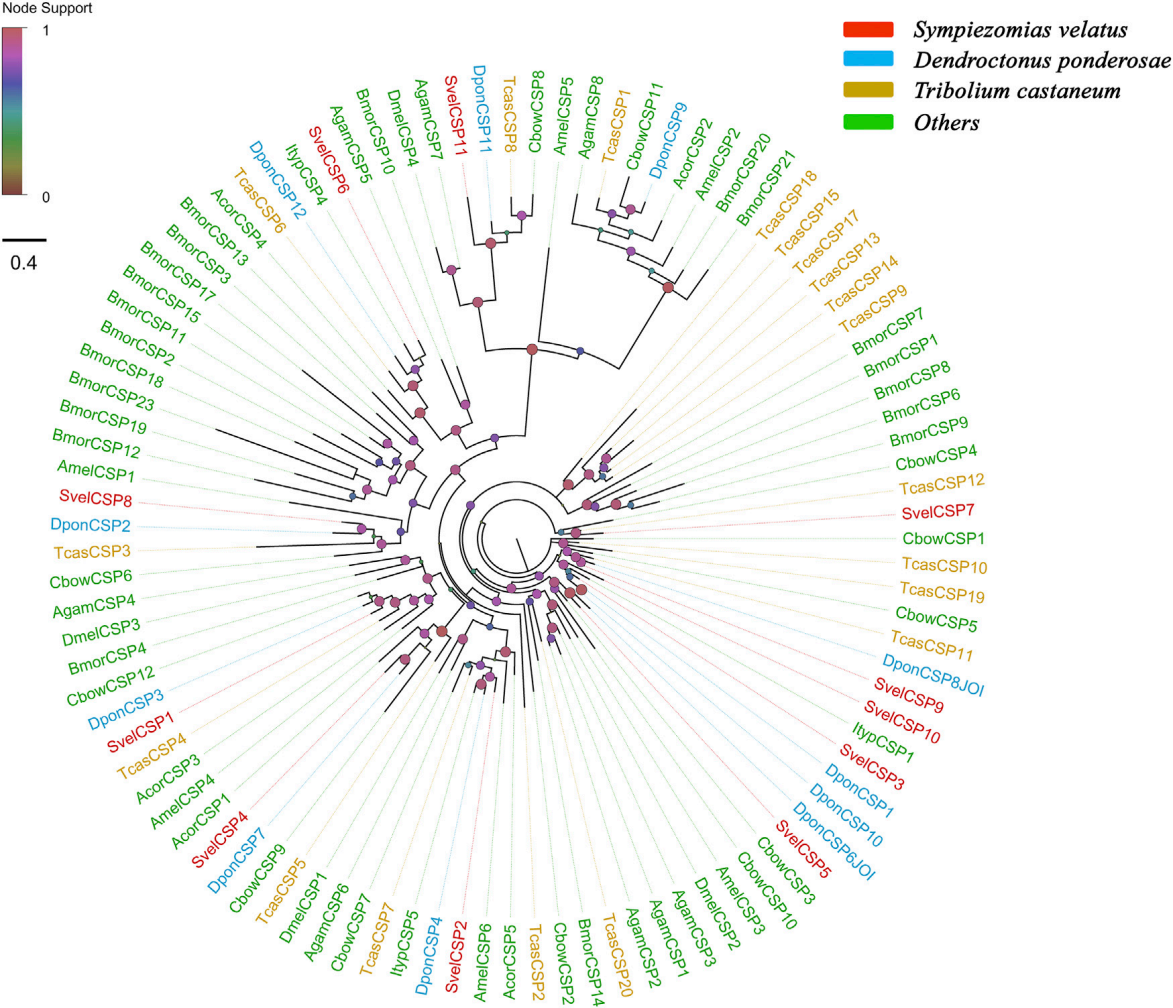
Phylogenetic tree of candidate chemosensory proteins (CSPs). The CSP sequences included were from *Sympiezomias velatus* (Svel, red), *Dendroctonus ponderosae* (Dpon, blue), *Tribolium castaneum* (Tcas, yellow), and other species (green), including *Drosophila melanogaster* (Dmel), *Anopheles gambiae* (Agam), *Bombyx mori* (Bmor), *Colaphellus bowringi* (Cbow), *Anomala corpulenta* (Acor) and *Ips typographus* (Ityp). The unrooted tree was constructed using FastTree based on a MAFFT alignment. The local support values based on the Shimodaira-Hasegawa (SH) test are indicated by the colored circles and increase with the brightness and size of the circles.

Eleven unigenes coding for SvelCSPs were identified in the combined transcriptome assembly, and full-length ORFs were obtained for 10 putative *CSP*s (*SvelCSP1-10*). All SvelCSPs contained OS-D domains (Pfam ID: PF03392). Signal peptide prediction showed that all the full-length sequences with the exception of SvelCSP10 had signal peptide sequences with a length of 17–26 amino acids (Supplementary Table S3). In general, the amino acid sequence of CSPs has four highly conserved cysteine residues and thus forms two interlocking disulfide bonds (C1–C2 and C3–C4) (Angeli et al., 1999). In addition, all SvelCSPs follow the classic cysteine spacing pattern C1-X_6-_C2-X_18-_C3-X_2-_C4 (Supplementary Figure S1D) (Xu et al., 2009). An unusually large *CSP*, *SvelCSP6*, which contained the complete ORF encoding a protein of 367 amino acids with a prolonged C-terminus, was also found. Homology and phylogenetic analyses revealed that SvelCSP6 is the homologue of DponCSP12 (70.1% identity) (Andersson et al., 2019). The majority of the identified SvelCSPs exhibited simple orthologous relationships with their counterparts from other beetle species (Figure 2).

### Odorant Receptors

**FIGURE 3:**
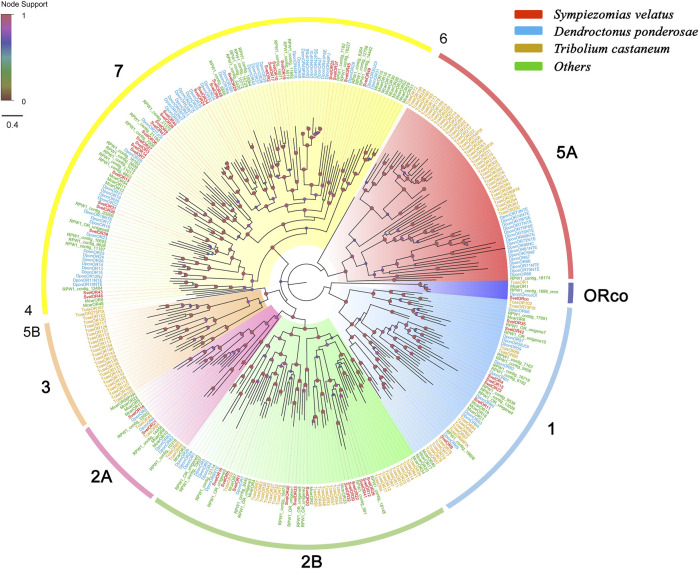
Phylogenetic tree of candidate odorant receptors (ORs) rooted with the conserved odorant receptor coreceptor (ORco). The OR sequences included were from *Sympiezomias velatus* (Svel, red), *Dendroctonus ponderosae* (Dpon, blue), *Tribolium castaneum* (Tcas, yellow) and other species (green), including *Megacyllene caryae* (Mcar) and *Rhynchophorus ferrugineus* (RPW1_X). The tree was constructed using FastTree based on a MAFFT alignment. The local support values based on the Shimodaira-Hasegawa (SH) test are indicated by the colored circles and increase with the brightness and size of the circles.

We identified 62 unigenes coding for SvelORs in the transcriptome assembly. Of these genes, 31 contained a full-length ORF that encoded proteins with more than 350 amino acids, and the remaining were partial sequences. All SvelORs contained 7tm_6 domains (Pfam ID: PF02949) that is a transmembrane protein domain having seven helices. According to TMHMM, the putative SvelORs with full-length protein sequences were predicted to contain four to eight transmembrane domains, and the other SvelORs were predicted to have zero to seven transmembrane domains (Supplementary Table S4). Fifty SvelORs with lengths of more than 200 amino acids were included in the phylogenetic analysis with representative ORs from several other coleopteran species. With the exception of SvelORco, the identified SvelORs were found within subgroups 1, 2A, 2B and 7. The majority of SvelORs were present within coleopteran OR subgroup 7 (28 SvelORs), followed by subgroup 2B (12 SvelORs) and subgroup 1 (seven SvelORs). Additionally, two SvelORs were clustered into subgroup 2A. Species-specific expansions of ORs were also found in *S. velatus*, but the largest expansion contained only four SvelORs (in subgroup 7) (Figure 3).

### Gustatory Receptors

**FIGURE 4:**
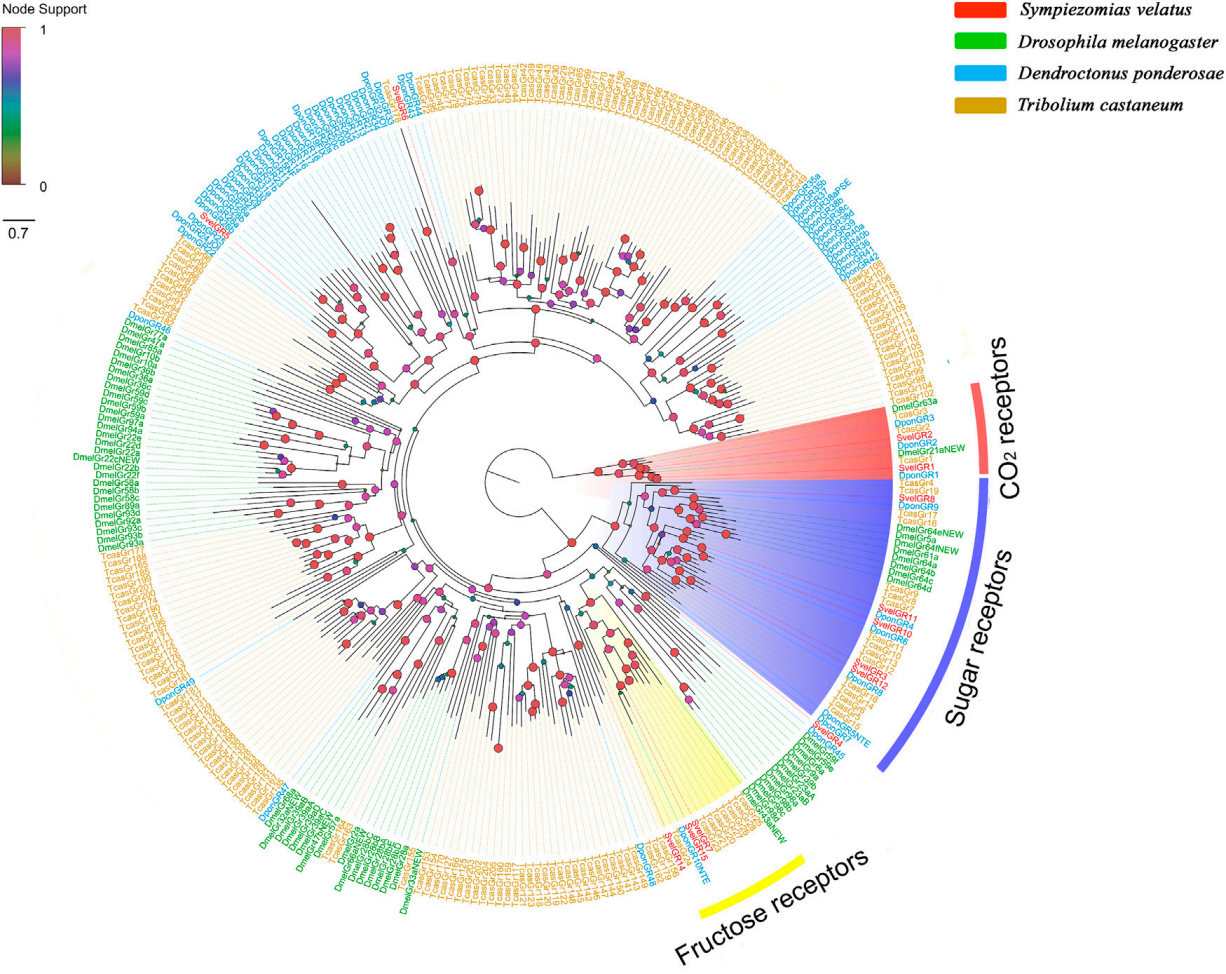
Phylogenetic tree of the candidate gustatory receptors (GRs), rooted with the conserved lineage of putative CO_2_/sugar receptors. The GR sequences included were from *Sympiezomias velatus* (Svel, red), *Dendroctonus ponderosae* (Dpon, blue), *Tribolium castaneum* (Tcas, yellow) and *Drosophila melanogaster* (Dmel, green). The tree was constructed using FastTree based on a MAFFT alignment. The local support values based on the Shimodaira-Hasegawa (SH) test are indicated by the colored circles and increase with the brightness and size of the circles.

Fifteen unigenes encoding putative GRs were identified from the *S. velatus* antennal transcriptome. Among these unigenes, nine represented full-length sequences encoding proteins with more than 264 amino acids, and the remaining *GR*s were partial fragments encoding overlapping but distinct sequences. Nine SvelGRs contained 7tm_7 domains (Pfam ID: PF08395) and the remaining six SvelGRs contained trehalose receptor domains (Pfam ID: PF06151) (Supplementary Table S4). A phylogenetic analysis revealed that SvelGR1 and SvelGR2 were clustered into the carbon dioxide (CO_2_) receptor branch. SvelGR1 was an ortholog of TcasGR1 (69.4% identity), and SvelGR2 was an ortholog of TcasGR2 (74.4% identity). However, no ortholog of the third *T. castaneum* CO_2_ receptor (TcasGR3) was found in *S. velatus*. Five SvelGRs (SvelGR3/8/10/11/12) were grouped into the branch of known sugar receptors, and three SvelGRs (SvelGR7/14/15) were grouped into the branch of known fructose receptors (Figure 4).

### Ionotropic Receptors

**FIGURE 5:**
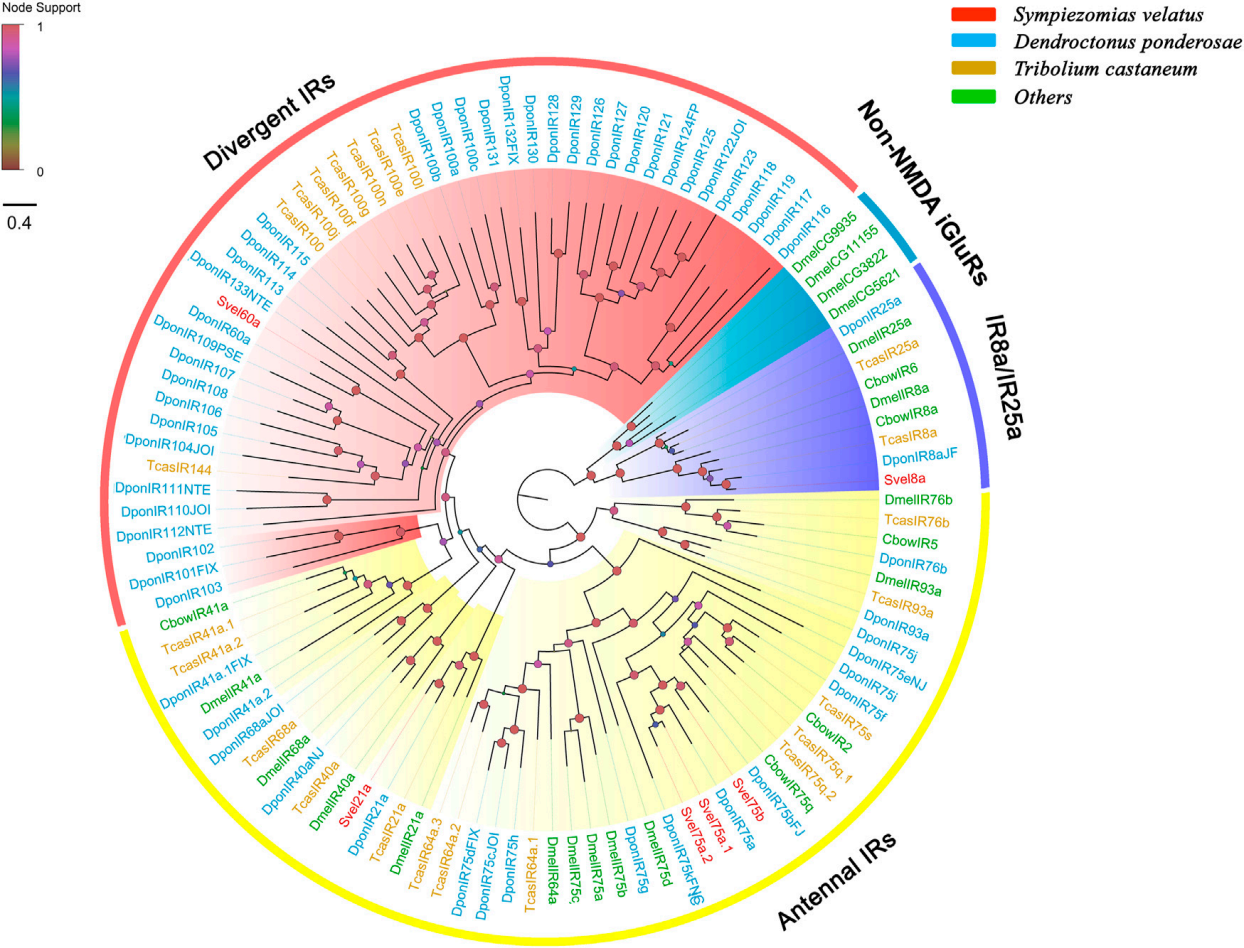
Phylogenetic tree of the candidate ionotropic receptors (IRs) rooted with the conserved lineages of IR8a and IR25a proteins. The IR sequences included were from *Sympiezomias velatus* (Svel, red), *Dendroctonus ponderosae* (Dpon, blue), *Tribolium castaneum* (Tcas, yellow) and other species (green), including *Drosophila melanogaster* (Dmel, green) and *Colaphellus bowringi* (Cbow). The tree was constructed using FastTree based on a MAFFT alignment. The local support values based on the Shimodaira-Hasegawa (SH) test are indicated by the colored circles and increase with the brightness and size of the circles.

We identified six unigenes encoding putative IRs from the *S. velatus* antennal transcriptome. Only one unigene contained a full-length ORF, which encoded a protein sequence with 887 amino acids, and the remaining unigenes were partial fragments. Except for the shortest sequence SvelIR75a.1, all SvelIRs contained ligand-gated ion channel domians (Pfam ID: PF00060) and were predicted to have three transmembrane domains according to TMHMM, which is characteristic of the IR family (Supplementary Table S4). Given IRs were revealed to be a variant subfamily of ionotropic glutamate receptors (iGluRs) and closely related with non-N-methyl-D-aspartic acid (NMDA) iGluRs (Croset et al., 2010), the previously reported non-NMDA iGluR representatives were also included in the phylogenetic analysis. All the included iGluRs/IRs were clustered into several phylogenetic groups, including non-NMDA iGluRs, IR coreceptors (IR8a/25a), antennal IRs and divergent IRs (Figure 5). Four SvelIRs were clustered into the group of antennal IRs. SvelIR8a was assigned to the coreceptor branch and clustered with IR8a orthologs from *C. bowringi*, *D. ponderosae*, *T. castaneum* and *D. melanogaster*. However, no IR25a ortholog was detected in the *S. velatus* antennal transcriptome. SvelIR60a was an homologue of Dpon60a (43.5% identity) and clustered into a branch of divergent IRs. In addition, the more ancient IR8a/25a lineage neighbored the non-NMDA iGluRs group in our phylogenetic analysis, which was in agreement with previous findings (Figure 5) (Croset et al., 2010; Zhang X. et al., 2019).

### Sensory Neuron Membrane Proteins

**FIGURE 6:**
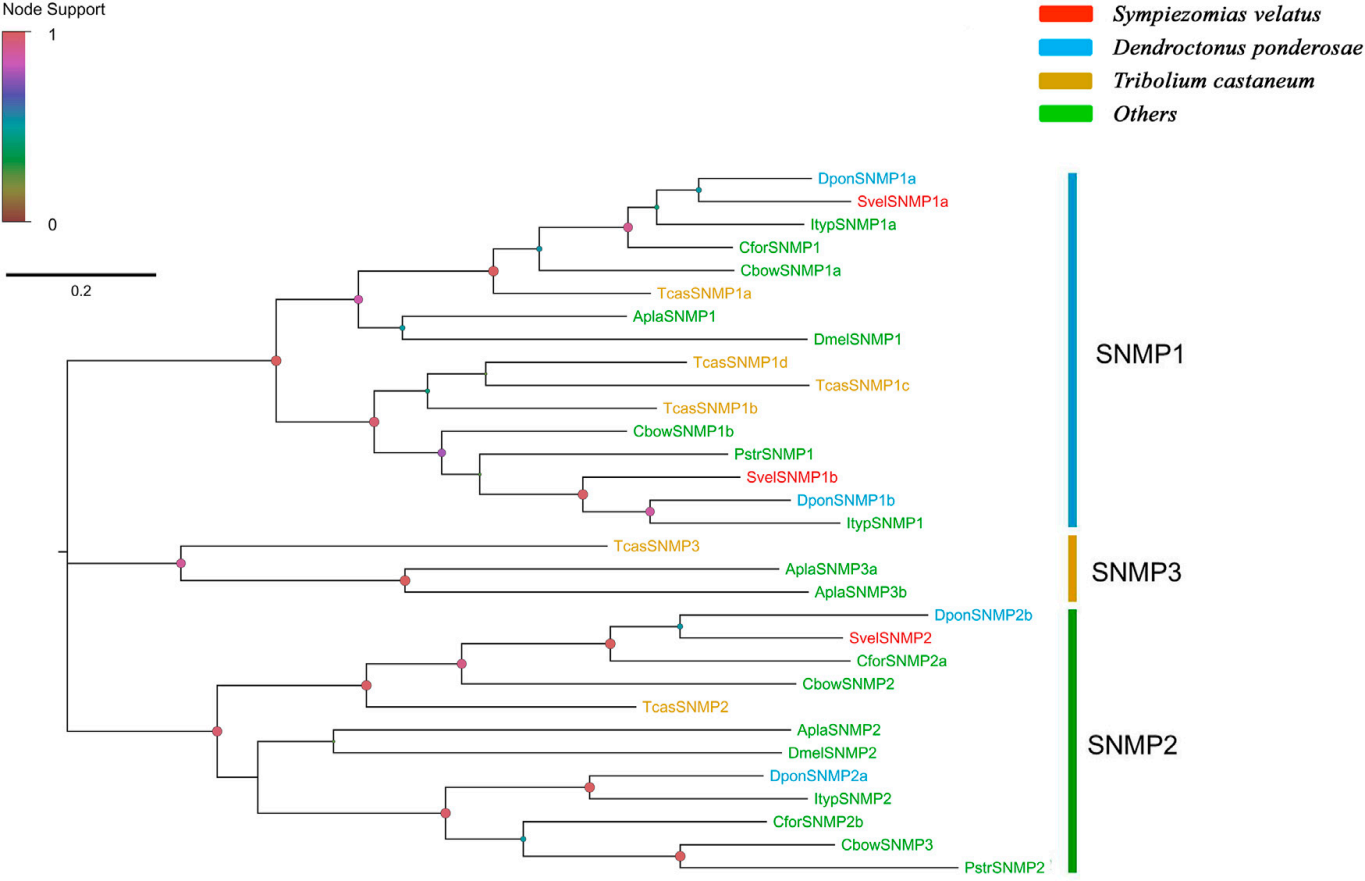
Phylogenetic tree of the candidate sensory neuron membrane proteins (SNMPs). The SNMP sequences included were from *Sympiezomias velatus* (Svel, red), *Dendroctonus ponderosae* (Dpon, blue), *Tribolium castaneum* (Tcas, yellow), and other species (green), including *Drosophila melanogaster* (Dmel), *Agrilus planipennis* (Apla), *Colaphellus bowringi* (Cbow), *Cylas formicarius* (Cfor), *Ips typographus* (Ityp) and *Phyllotreta striolata* (Pstr). The tree was constructed using FastTree based on a MAFFT alignment. The local support values based on the Shimodaira-Hasegawa (SH) test are indicated by the colored circles and increase with the brightness and size of the circles.

Three unigenes encoding putative SNMPs were identified in the *S. velatus* transcriptome assembly, and these unigenes included two members of the *SNMP1* subfamily (*SvelSNMP1a* and *SvelSNMP1b*) and one member of the *SNMP2* subfamily (*SvelSNMP2*). These unigenes had full-length ORFs encoding proteins longer than 530 amino acids in length. All SvelSNMPs contained CD36 domians (Pfam ID: PF01130). The transmembrane domain prediction showed that all three SvelSNMP sequences have a typical structure of two transmembrane domains (Supplementary Table S4). A phylogenetic analysis revealed that SvelSNMP1a and SvelSNMP1b were clustered into the SNMP1 branch, and SvelSNMP2 was clustered into the SNMP2 branch (Figure 6).

### Gene Expression Profiles

**FIGURE 7:**
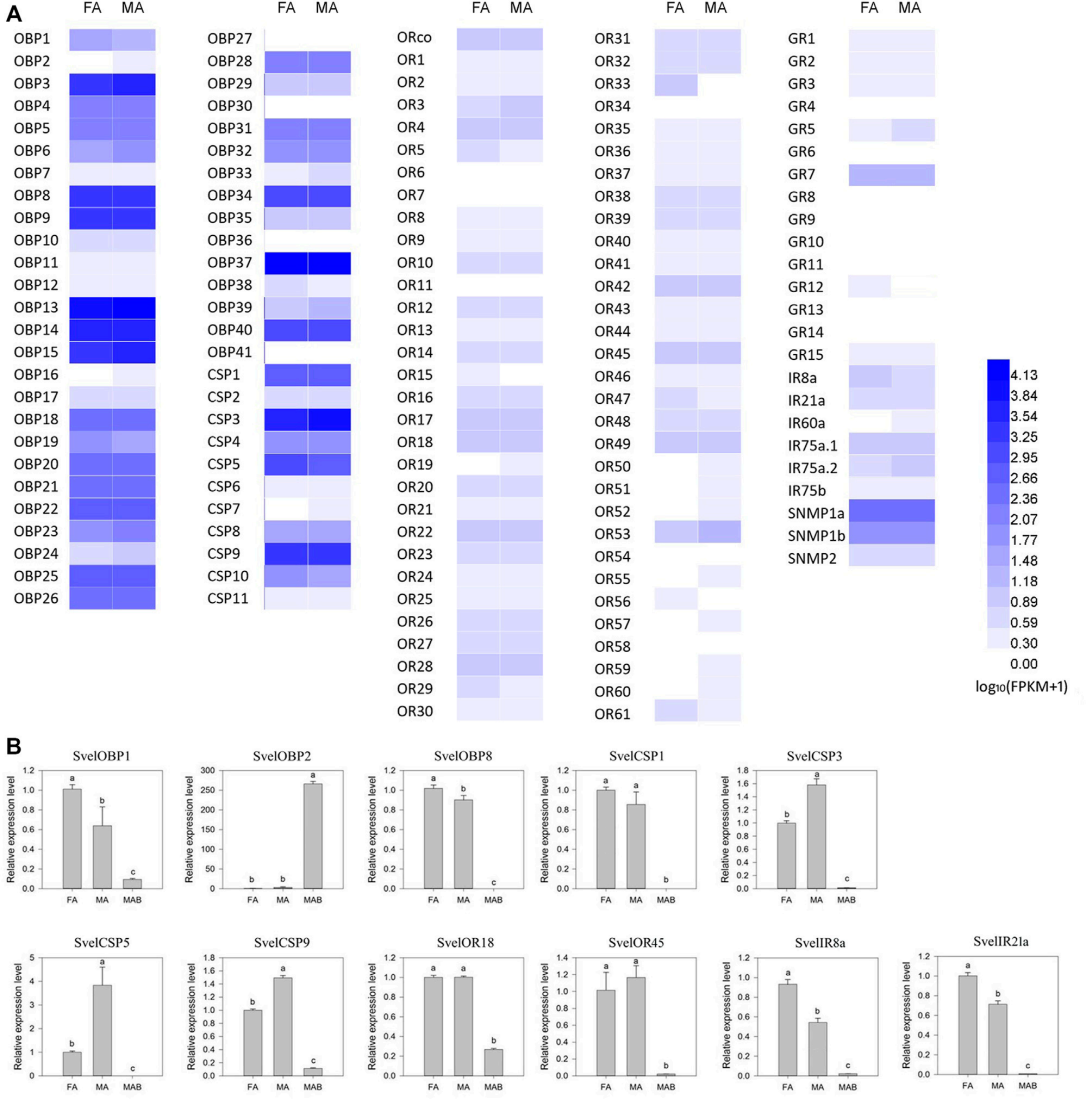
Expression profiles of candidate chemosensory genes in *Sympiezomias velatus* based on average RPKM values **(A)** and determined by RT-qPCR analysis **(B)**. FA, female antennae; MA, male antennae; MAB, male abdomen. The RT-qPCR data for each tissue were first normalized against *RPS20*, and female antennae were selected as the calibrator. The relative expression levels are means ± SE (*n* = 3) and were statistically analyzed by one-way ANOVA followed by Tukey’s test. Different lowercase letters indicate significant differences at the 0.05 level.

The expression profiles of 138 candidate chemosensory genes were analyzed in antennae of female and male *S. velatus* adults based on the average FPKM values of three replicates (Supplementary Table S5 and Figure 7A). The expression patterns of 11 genes were randomly selected and validated by RT-qPCR. RT-qPCR revealed that *SvelOBP1*, *8, SvelIR8a* and *21a* were female-biased and *SvelCSP3*, *5* and *9* were male-biased. However, the expression differences between female and male antennae were less than 2-fold except for *SvelCSP5*. The remaining tested genes displayed no sex-biased expression in the antennae (Figure 7B). These findings were basically consistent with the results indicated by the FPKM value.

Overall, the expression levels of the *S. velatus OBP*s in the antennal transcriptomes were higher (average FPKM value > 1000) than those of the five other chemoreception gene families. *SvelOBPs* (*SvelOBP3/14/15/37*) belonging to the *ABPX* subfamily according to the phylogenetic analysis were highly expressed in both female and male antennae of *S. velatus*. The most highly expressed *OBP* gene was *SvelOBP37* followed by *SvelOBP13*, *14* and *15*. Four *SvelCSP*s (*SvelCSP1/3/5/9*) were richly expressed in both antennae, and among them, *SvelCSP3* exhibited the highest expression level. In the antennal transcriptomes, the genes coding for three receptor families were present at obviously lower abundance (the average FPKM value < 10) than the other olfactory gene families, among which the average expression levels of *GR*s were lowest. *SvelORco*, *SvelOR18*, *28* and *53* exhibited high expression among all *OR*s. *SvelGR7* exhibited a much higher expression level than the other *GR*s, and CO_2_ receptor transcripts (*SvelGR1* and *SvelGR2*) were also relatively abundant. Among all *IRs*, *SvelIR75a.1*, *75a.2* and *8a* were relatively highly expressed. *SvelSNMP2* displayed significantly lower expression than the two *SNMP1* members. In addition, the majority of candidate chemosensory genes did not display sex-biased expression according to the threshold for DEGs. However, it is worth noting that *SvelOR33* displayed female-biased expression (Supplementary Table S5 and Figure 7A).

### Functional Characterization of SvelOBP15

As described above, *SvelOBP37*, *14* and *15* were found to be among the four most highly expressed *OBP* genes in antennae. Meanwhile they were the top three *ABP* genes abundantly expressed in antennae at the adult stage. These suggested that further investigation is necessary regarding their roles in olfactory perception. We attempted to investigate their binding function, but regrettably, we failed to express active proteins for *SvelOBP37* and *SvelOBP14*.

**FIGURE 8:**
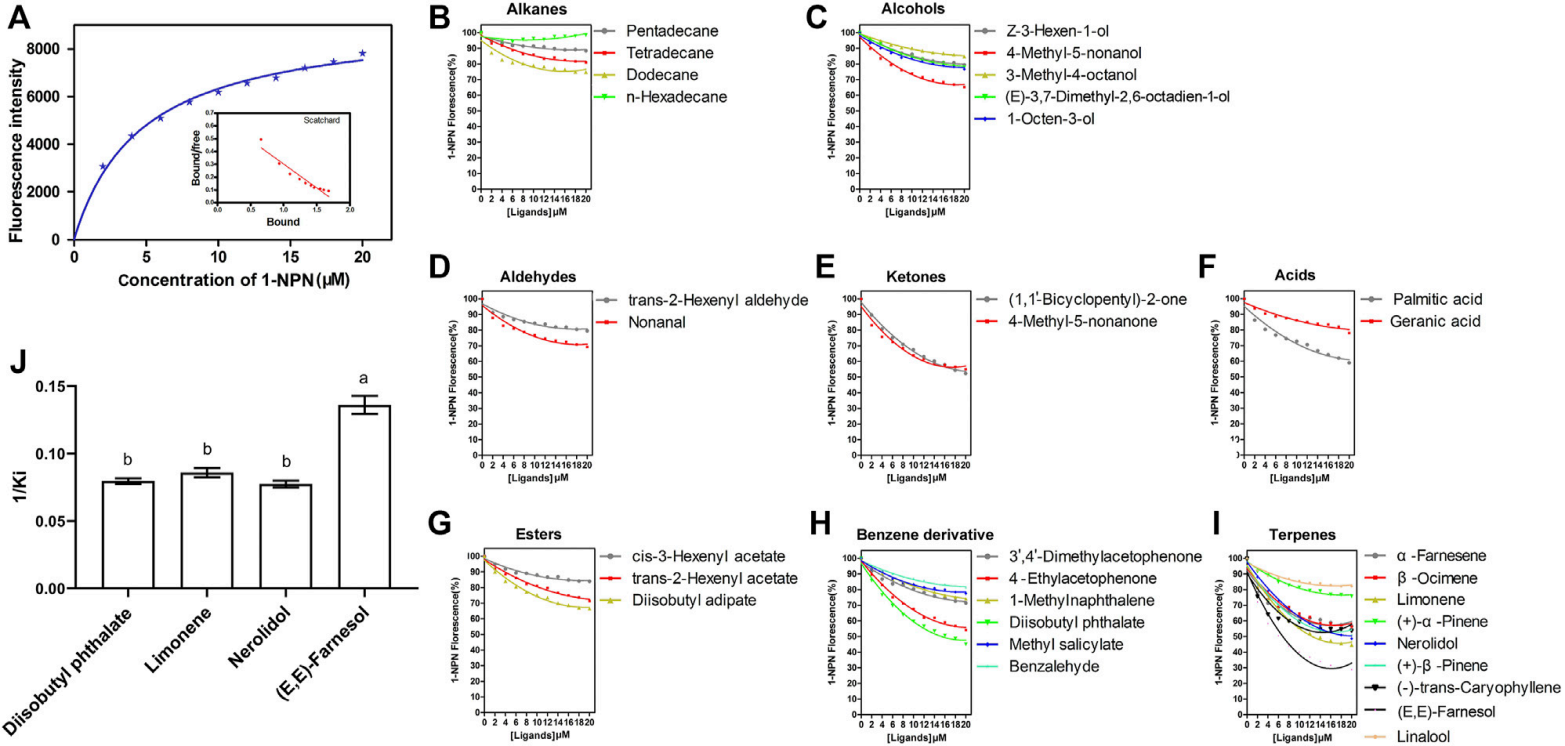
Fluorescence competitive binding assay showed the binding property of SvelOBP15 of *Sympiezomias velatus* to all 33 tested chemicals. **(A)** represents the binding curve and Scatchard plot of the SvelOBP15 protein with 1-NPN; **(B–I)** represent competitive binding curves of SvelOBP15 to aliphatic hydrocarbons, alcohols, aldehydes, ketones, esters, acids, benzene derivatives and terpenes, respectively; **(J)** represents reverse values of dissociation constants (Ki) of SvelOBP15, and different lowercase letters indicate significant differences at the 0.05 level (one-way ANOVA followed by Tukey’s test).

*SvelOBP15* was expressed in a bacterial system. Its ORF encodes 149 amino acids, with a theoretical Mw of 17.05 KD and a pI of 4.64. SvelOBP15 contains six conserved cystine residues and belongs to the classical OBP subfamily. SDS-PAGE analysis revealed that the target protein was mainly present in inclusion bodies. The molecular weight of the target protein containing a His-tag was approximately ∼21kD, in line with expectations (Supplementary Figure S3A). After the His-tag was removed by digestion with rEK and the protein was purified again, a single band of predicted size was observed, which was used for the fluorescence competition binding test (Supplementary Figure S3B). The ligand binding affinity of recombinant SvelOBP15 was determined by a fluorescence competitive binding assay. First, by titrating SvelOBP15 with increasing concentrations of 1-NPN, a saturation and linear Scatchard plot was observed, with a dissociation constant of 4.68 ± 0.16 μmol/L (Figure 8A), indicating that 1-NPN was a suitable fluorescent reporter. Fluorescence competition binding experiments were used to measure the binding affinities of 33 candidate compounds with SvelOBP15. The results showed that SvelOBP15 displayed high binding affinities with nerolidol, diisobutyl phthalate, limonene, and farnesol (Ki = 7.36–12.94 μmol/L) (Supplementary Table S2 and Figures 8B–J). Among them, farnesol exhibited the strongest binding to SvelOBP15 with a dissociation constant of 7.36 ± 0.36 μmol/L. SvelOBP15 also displayed similar strong binding capacities to limonene, diisobutyl phthalate and nerolidol, and the dissociation constants are 11.66 ± 0.47 μmol/L, 12.57 ± 0.34 μmol/L and 12.94 ± 0.43 μmol/L, respectively (Supplementary Table S2 and Figure 8).

## Discussion

In the present study, we identified 138 candidate chemosensory genes from the *S. velatus* antennal transcriptome, including 41 *OBP*s, 11 *CSP*s, 62 *OR*s, 15 *GR*s, six *IR*s and three *SNMP*s. This is the first comprehensive characterization of olfactory genes in this weevil species. The findings will extend our knowledge of the olfactory system in Coleoptera, the largest order of insects, and provide a basis for further research on chemoreception mechanisms in *S. velatus*. However, the genes identified in this study do not necessarily represent the total number of six related chemosensory gene families in *S. velatus.* On the one hand, some genes might be overlooked during RNA-seq because they might not be expressed or are expressed at very low levels in the tested transcriptome. On the other hand, olfactory genes that are too divergent from the known query gene sequences might be missed in the transcriptome analysis based on the homology search methodology.

Previous studies have shown that OBPs and CSPs are present not only in antennae but also in non-chemosensory tissues. For example, both of them have been found to be expressed in pheromone glands and involved in the release of pheromones (Dani et al., 2011; Gu et al., 2013; Jacquin-Joly et al., 2001; Li et al., 2008). They are also present in eggs with putative roles in development (Maleszka et al., 2007; Marinotti et al., 2014). Besides, a CSP in the moth proboscis has been shown to be involved in reducing water surface tension rather than chemoreception (Liu et al., 2014). Similarly, quantitative PCR tissue expression profiling revealed the existence of *S. velatus OBP* that was also expressed in non-chemosensory tissues, such as *SvelOBP2* (Figure 7B). Moreover, some *OBP* or *CSP* genes were predominantly expressed in non-antennary tissues. For example, the majority of the *T. castaneum CSP* and the Minus-C *OBP* transcripts have been reported to exist at low abundances in the antennae or mouthparts (Dippel et al., 2014). Such genes in *S. velatus* were likely missed based on antennal transcriptome sequencing. In this study, 41 candidate *OBP*s and 11 candidate *CSP*s were obtained from the antennae transcriptomic data of *S. velatus*. These numbers were very close to those found in the *R. ferrugineus* antennal transcriptome (Antony et al., 2016) and *D. ponderosae* genome (Andersson et al., 2019). Thus, it can be speculated that the real numbers of *OBP*s and *CSP*s in *S. velatus* might be similar to those in *R. ferrugineus* but more than those in *D. ponderosae*. It is worth noting that an unusually large OBP, SvelOBP10, was identified. It is the homolog of *D. ponderosae* OBP4 which is a Minus-C OBP “tetramer” (Andersson et al., 2019). This novel type of OBP was also identified in three other Curculionidae species: the red palm weevil *R. ferrugineus* (Antony et al., 2016), the round-headed pine beetle *Dendroctonus adjunctus* (Torres-Huerta et al., 2020) and the Yunnan pine shoot beetle *Tomicus yunnanensis* (GFJU01117056)*.*

ORs have been studied more intensively than other olfactory proteins. In this study, we annotated 62 *OR*s from the *S. velatus* antennal transcriptome, which are more than those found in *L. oryzophilus* (41 *OR*s) and *C. formicarius* (54 *OR*s) but less than those found in *R. ferrugineus* (76 *OR*s) and *D. ponderosae* (86 *OR*s) in the same Curculionidae family (it is worth noting that the annotation of the last weevil species was based on genome-scale data). To confirm the subfamily grouping, we built a phylogenetic tree using a total of 333 OR sequences from *S. velatus*, *D. ponderosae* (Andersson et al., 2019), *R. ferrugineus* (Antony et al., 2016), *T. castaneum* (Engsontia et al., 2008), and *M. caryae* (Mitchell et al., 2012). This sampling included representatives from three families in Coleoptera (Svel, Rfer and Dpon: Curculionidae; Tcas: Tenebrionidae; and Mcar: Cerambycidae). According to recent studies, coleopteran ORs were divided into nine monophyletic subgroups (named 1, 2A, 2B, 3, 4, 5A, 5B, 6, and 7) (Andersson et al., 2019; Mitchell et al., 2020; Wu et al., 2020), which are different from the grouping proposed in previous studies (Engsontia et al., 2008; Mitchell et al., 2012; Andersson et al., 2013; Li X. et al., 2015; Li X.-M. et al., 2015). The subfamily grouping presented herein agrees with the former. Furthermore, the majority of *S. velatus* ORs were grouped into subfamilies 1, 2A, 2B, and 7, which are similar to the ORs from other curculionids (Andersson et al., 2019) (Antony et al., 2016). The species-specific expansion of ORs is widespread in coleopteran insects (Engsontia et al., 2008; Mitchell et al., 2012; Li X. et al., 2015; Li X.-M. et al., 2015; Andersson et al., 2019). There might be two reasons for these expansions: paralogous replication and alternative splicing (Andersson et al., 2019). These expansions of ORs were also found in *S. velatus* that may reflect the different niches and might result from adaptation to the environment. In addition, apparent orthologous relationships existed between *S. velatus* and the two other curculionids in the odorant-specific OR branch, which suggest that these ORs might be universal in weevils and play conserved biological roles. Although there is limited information on pheromone receptors in coleopterans, the cerambycid beetle *M. caryae* reportedly has three *OR*s (*McarOR3*/*5*/*20*) involved in the perception of aggregation pheromone molecules (Mitchell et al., 2012). Homologues of these functionally characterized *OR*s were also identified in *S. velatus*. *Sv*el*OR8* is the homologue of RPW contig19606/*DponOR65*, and these *OR*s were clustered into a lineage containing *McarOR20*. *SvelOR18* is the homologue of RPW contig22002/*DponOR9*, and these *ORs* were clustered into a lineage containing *McarOR5*. Whether *SvelOR8* and *SvelOR18* have similar functions to *McarOR20* or *McarOR5* still needs further investigation. It is also worth noting that *SvelOR33* displayed a female-biased expression pattern, which implies that it might be involved in olfactory perception related to egg-laying behavior.

In general, gustatory receptors are mainly expressed in gustatory sensory neurons (GSNs) of taste organs distributed on the labial palp, internal mouthpart organs, legs, margins of the wings and ovipositors in females (Clyne et al., 2000; Scott, 2018). Many *GR*s are also expressed in the antenna. Moreover, the number of *GR*s expressed in mouthparts is proposed to be similar to that in antennae, and taste perception appears to be equally distributed between these two organs (Dippel et al., 2016). In *D. melanogaster*, GR21a and GR63a are reportedly highly expressed in antennae and form a heterodimer to participate in CO_2_ detection (Robertson and Kent, 2009). In contrast, the *T. castaneum* genome contains three CO_2_ receptor-encoding genes. The orthologs of the two *D. melanogaster* gustatory receptors found in *T. castaneum* were GR1 and GR3 (Richards et al., 2008). In *S. velatus*, we found two CO_2_ receptors but did not find the ortholog of TcasGR3/Dmel63a. Whether this finding means that the SvelGR3-encoding gene was truly lost and *S. velatus* thus lost the related ability remains unclear. A recent study on the larvae of a chrysomelid root herbivore, *Diabrotica virgifera*, showed that GR2 is responsible for the perception of plant-derived CO_2_ and plays a crucial role in long-distance host location and assessment of the plant nutritional status (Arce et al., 2020). Whether *S. velatus* GRs have similar physiological or behavioral functions is worth further study. In addition to CO_2_ receptors, five *S. velatus* GRs (SvelGR3/8/10/11/12) were identified as putative sugar-taste receptors because they were grouped with DmelGR5a, which responds to many sugars (Marella et al., 2006; Scott, 2018). Some GR members, such as Gr32a, Gr33a, Gr66a, Gr89a, and Gr93a, mediate bitter taste in *Drosophila* (Chen and Dahanukar, 2020), but we did not find their orthologs in the *S. velatus* antennal transcriptome*.* Three *S. velatus* GRs (SvelGR7/14/15) were speculated to be putative fructose receptors because they were grouped into the branch including DmelGr43a, a highly conserved GR outside the sugar-receptor clade and characterized as an internal fructose-sensing receptor (Freeman et al., 2014; Miyamoto and Amrein, 2014; Fujii et al., 2015).

IR is another crucial chemoreceptor family with functions in the sensing of smell, taste, temperature, and humidity (Benton et al., 2009; Croset et al., 2010; Rytz et al., 2013; Hussain et al., 2016; Knecht et al., 2016). Based on their phylogenetic relationship, sequence characteristics and expression pattern, IRs are generally classified into conserved “antennal IRs” and species-specific “divergent IRs” (Croset et al., 2010). The former are primarily expressed in the antennae and involved in the detection of a limited number of odors, including acids, amines and amino acids; the latter are mainly expressed in the gustatory neurons of non-antennal tissues and implicated in gustation (Croset et al., 2010; Rytz et al., 2013). Our phylogenetic analysis revealed the existence of a set of conserved antennal IRs in *S. velatus* (homologs of IR21a/75a.1/75a.2/75b), which suggest their potential roles in olfaction. IRs commonly perform their functions through the combination of coreceptors (e.g., IR8a, IR25a, and IR76b) with stimulus-specific IRs to form heteromeric ligand-gated ion channels for the recognition of different ligands (Rytz et al., 2013). In this study, we only found one coreceptor ortholog in *S. velatus*, i.e., SvelIR8a. As a coreceptor, IR8a might participate in a wide range of physiological processes in insects; for instance, *Manduca sexta* IR8a has been demonstrated to be essential for oviposition selection through acid-mediated fecal avoidance (Zhang X. et al., 2019). Given that IR25a is the most highly conserved IR across species (Croset et al., 2010) and due to the existence of this coreceptor in closely related species of *S. velatus* (Antony et al., 2016; Bin et al., 2017; Wen et al., 2018; Zhang Y. et al., 2019), IR25 was likely not truly lost from *S. velatus* and was simply missed in the present transcriptome analysis. Likewise, *S. velatus* ortholog of IR76b was either not identified. In *D. melanogaster*, this coreceptor is broadly expressed in proboscis gustatory neurons and olfactory neurons and has been shown to mediate the sensing of low salt levels, polyamines and amino acid taste (Zhang et al., 2013; Hussain et al., 2016; Ganguly et al., 2017). Unlike *S. velatus*, *R. ferrugineus* IR76b has been found in the antennal transcriptome (Antony et al., 2016).

SNMP was first identified in the dendritic membrane of olfactory receptor neurons (ORNs) implicated in sex pheromone perception in the wild silk moth *Antheraea polyphemus* (Rogers et al., 1997). SNMPs appear to function as cofactors for odorant receptors responsible for pheromone detection in *Drosophila* and moths (Benton et al., 2007). There are generally two subgroups (SNMP1 and SNMP2) in most insect species (Vogt et al., 2009). Later, SNMP3 was identified in coleopteran insects such as *T. castaneum* and *A. planipennis* (Dippel et al., 2016; Andersson et al., 2019). Recently, a novel SNMP subgroup, named as SNMP4, was discovered in the family Scarabaeidae based on phylogenetic analyses, sequence characteristics, and gene structure (Zhao et al., 2020). In the present study, we found two orthologs for SNMP1 and one single ortholog for SNMP2 in *S. velatus*. This is consistent with the results reported in *E. scrobiculatus*, *E. brandti* (Wen et al., 2018) and *I. typographus* (Andersson et al., 2013). Similarly, only orthologs for SNMP1 and SNMP2 have been identified from the palm weevil *R. ferrugineus* (three orthologs for each subgroup) (Antony et al., 2016), the sweet potato weevil *C. formicarius* (one ortholog for SNMP1 and two orthologs for SNMP2) (Bin et al., 2017) and the bark beetle *D. ponderosae* (two orthologs for each subgroup) (Andersson et al., 2019).

After the identification of the chemosensory genes in this weevil species, we focused on the function of one abundantly expressed OBP in adult antennae, SvelOBP15, and investigated its binding specificity using competitive binding experiments. Thirty-three compounds were tested, and only four compounds exhibited binding to this protein. SvelOBP15 showed certain binding activity with diisobutyl phthalate which is a volatile component emitted from elm seedlings (Cheng et al., 2010; Wang et al., 2017). This benzene derivative has been previously reported to be weakly bound by AquaOBP4 of the leaf beetle *Ambrostoma quadriimpressum*, the most abundant antennal-specific OBP in this closely related coleopteran species (Wang et al., 2017). The binding activity of AquaOBP4 is also narrow-spectrum, shown by only five protein-ligand combinations among 40 tested compounds related to the habitat. SvelOBP15 also has a specific binding preference for certain terpenoids, showing different affinities to farnesol, nerolidol and limonene. The binding affinity of SvelOBP15 to farnesol was the strongest, and this compound is both a host plant-derived volatile and a compound identified from female sex pheromone glands of click beetles (Yatsynin et al., 1996; Schnee et al., 2002). The contents of nerolidol and limonene are low in the volatiles of healthy peanut plants, but their emissions are significantly induced by herbivory damages (Cardoza et al., 2002). These results suggest that SvelOBP15 is involved in the perception of plant odorants. Meanwhile, SvelOBP15 cannot effectively bind with other tested compounds. For instance, dodecane, tetradecane, pentadecane and n-hexadecylthydride cannot be bound with SvelOBP15, suggesting that aliphatic hydrocarbons are probably not ligands of this OBP. Thus, we speculate that SvelOBP15 has obvious selective binding characteristics with host plant volatiles. Unfortunately, we cannot figure out whether SvelOBP15 has binding affinities with sex pheromone components since these components of *S. velatus* have not been identified so far. Moreover, only a small number of compounds showed binding to SvelOBP15 in the present study, which made it difficult to find particularly obvious rules in association with the chemical structural characteristics. Additionally, phylogenetic analysis revealed that SvelOBP15 is homologous with OBP28 of the maize weevil *S. zeamais*. SzeaOBP28 also has a narrow binding spectrum of odorant molecules and exhibited a binding affinity with bis (2-methoxyethyl) phthalate that is a structure analogue of diisobutyl phthalate (Zhang Y. et al., 2019), suggesting that the homologous OBP proteins are evolutionarily conserved and have similar biological functions. Taken together, it is speculated that OBP15 may play an important role in the recognition of olfactory odorants, especially in host localization. Given that its ability to bind most of the tested odorous ligands is weak, its main role in the process of locating host plants is speculated to recognize and bind some specific volatile components. Its specific functions still need further study. At the same time, this result also laid the foundation for the development of behavior modifiers such as *S. velatus* attractants.

## Data Availability

The data presented in the study are deposited in the NCBI SRA, accession numbers SSR17542735-SSR17542740.
