# Supplementary figures and images for: Candidate Chemosensory Genes Identified in the Adult Antennae of Sympiezomias velatus and Binding Property of Odorant-Binding Protein 15
Source: Front Physiol. 2022 May 31;13:907667. doi: 10.3389/fphys.2022.907667 (PMC9193972; doi:10.3389/fphys.2022.907667)

A


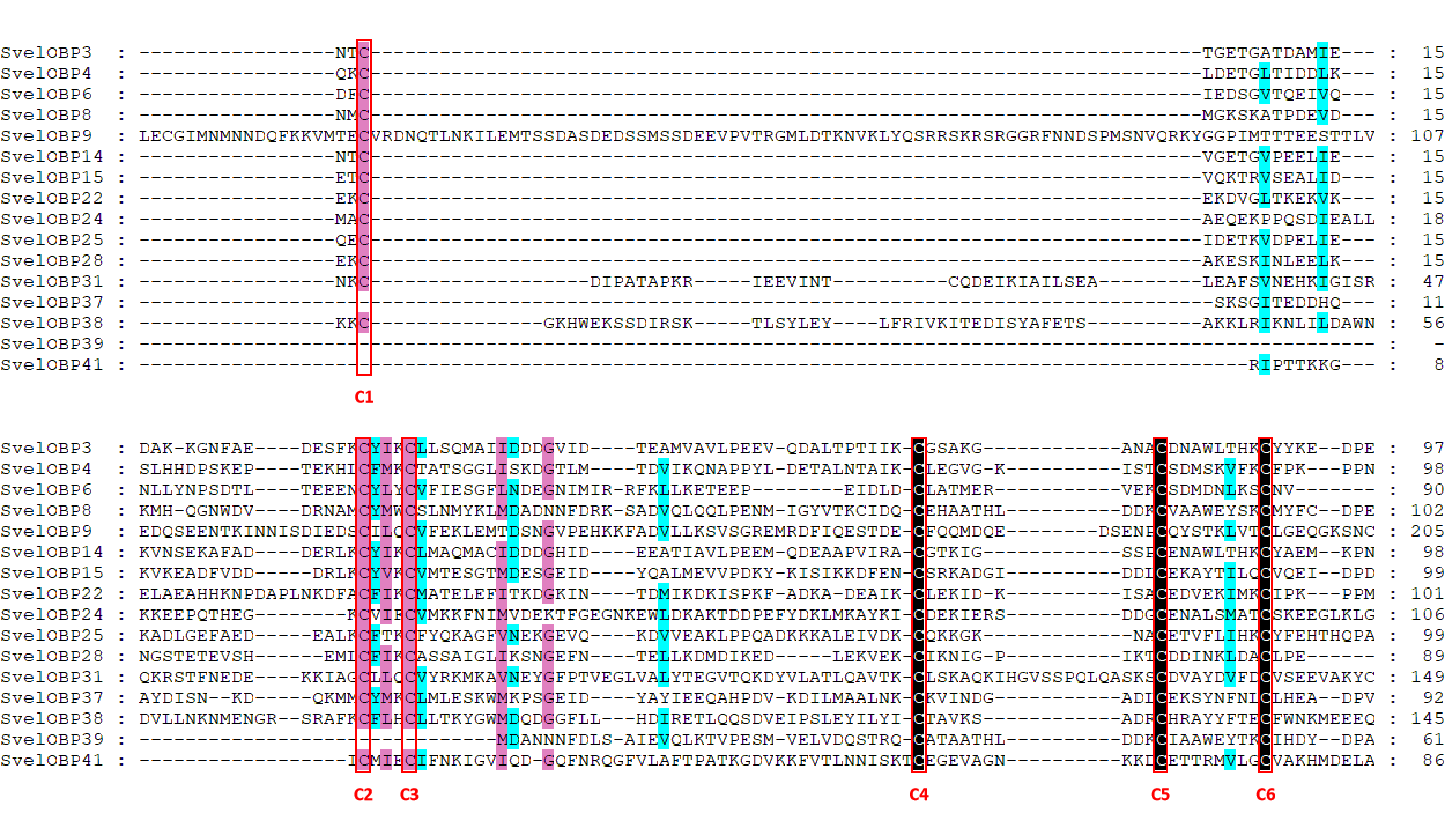


B


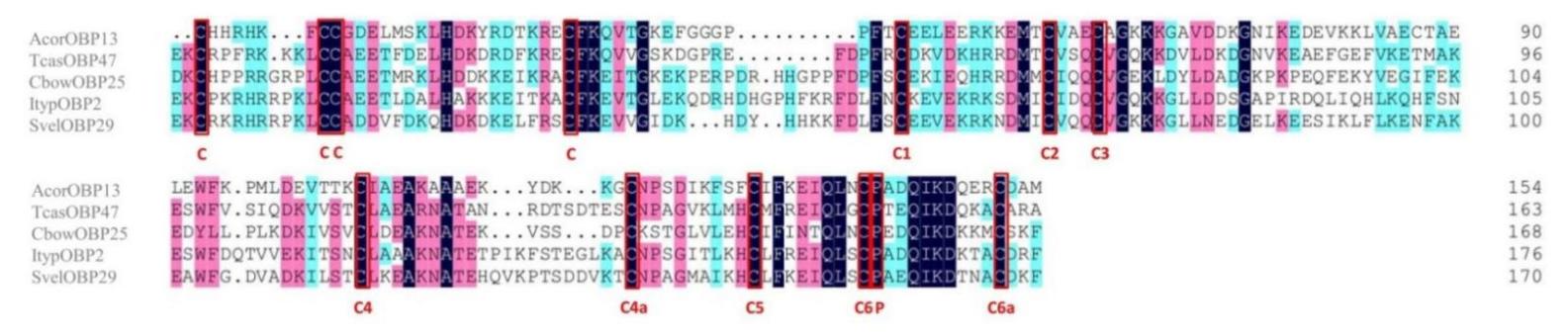


C


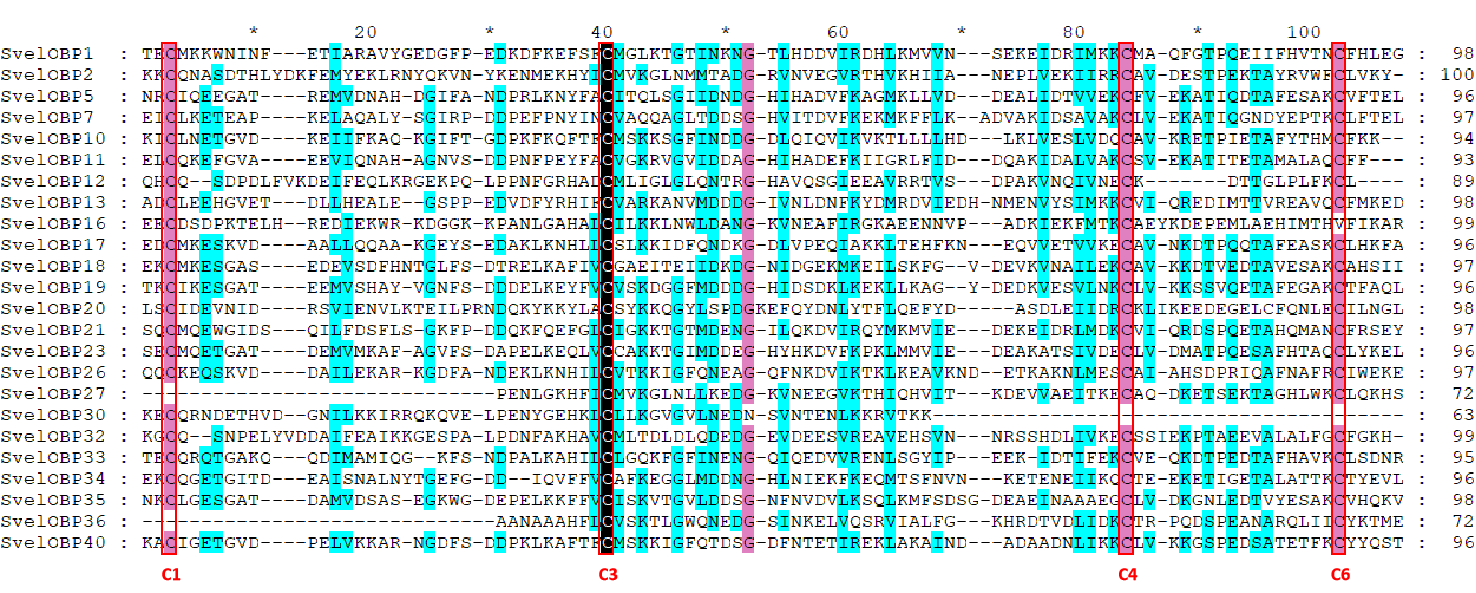


D


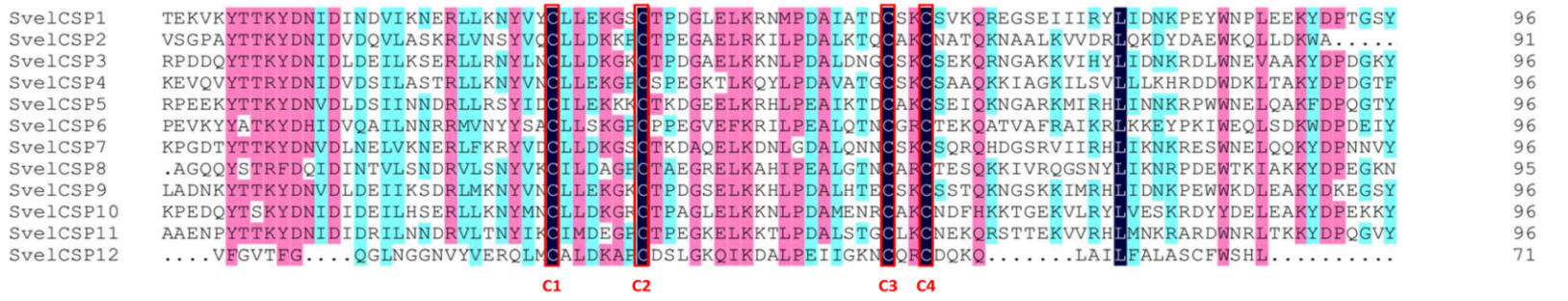


**Figure S1.** Amino acid alignments of Classic OBPs (A), Plus-C OBPs (B), Minus-C OBPs (C) and

CSPs (D).

Supplement: Supplementary file 1 [file Table1.DOCX]
