# Supplementary material for: Candidate Chemosensory Genes Identified in the Adult Antennae of Sympiezomias velatus and Binding Property of Odorant-Binding Protein 15
Source: Front Physiol. 2022 May 31;13:907667. doi: 10.3389/fphys.2022.907667 (PMC9193972; doi:10.3389/fphys.2022.907667)
Supplement: Supplementary file 2 [file Table7.docx]

**Table S4**. The Blastp match of *S. velatus* candidate ORs, GRs, IRs and SNMPs.

| **Gene name** | **ORF**  **(aa)** | **Complete ORF** | **TMD^a^ (No.)** | **Pfam ID** | **Best Blastp Match** | | | |
| --- | --- | --- | --- | --- | --- | --- | --- | --- |
|  |  |  |  |  | **Acc. number** | **Species** | **E value** | **Identity (%)** |
| SvelORco | 483 | Y | 7 | PF02949 | AOO35283.1 | *Rhynchophorus ferrugineus* | 0 | 90.0 |
| SvelOR1 | 385 | Y | 6 | PF02949 | [XP_019762033.1](https://www.ncbi.nlm.nih.gov/protein/1130235820?report=genbank&log$=protalign&blast_rank=1&RID=YXX1GHUR014) | *Dendroctonus ponderosae* | 7e-136 | 57.3 |
| SvelOR2 | 251 | N | 3 | PF02949 | [XP_030750008.1](https://www.ncbi.nlm.nih.gov/protein/XP_030750008.1?report=genbank&log$=prottop&blast_rank=2&RID=NK0YGFAN016) | *Sitophilus oryzae* | 1e-48 | 40.2 |
| SvelOR3 | 399 | Y | 4 | PF02949 | [XP_030746041.1](https://www.ncbi.nlm.nih.gov/protein/XP_030746041.1?report=genbank&log$=prottop&blast_rank=3&RID=NK16435001R) | *Sitophilus oryzae* | 3e-69 | 35.9 |
| SvelOR4 | 391 | Y | 4 | PF02949 | [XP_018579026.2](https://www.ncbi.nlm.nih.gov/protein/XP_018579026.2?report=genbank&log$=prottop&blast_rank=5&RID=NK1B6D9A014) | *Anoplophora glabripennis* | 1e-85 | 35.8 |
| SvelOR5 | 383 | Y | 7 | PF02949 | [XP_030753545.1](https://www.ncbi.nlm.nih.gov/protein/XP_030753545.1?report=genbank&log$=prottop&blast_rank=1&RID=NK1FUTE1014) | *Sitophilus oryzae* | 6e-10 | 45.4 |
| SvelOR6 | 278 | N | 4 | PF02949 | [XP_030764608.1](https://www.ncbi.nlm.nih.gov/protein/XP_030764608.1?report=genbank&log$=prottop&blast_rank=1&RID=NK1MNP5X01R) | *Sitophilus oryzae* | 3e-68 | 40.3 |
| SvelOR7 | 234 | N | 4 | PF02949 | [KAF7269990.1](https://www.ncbi.nlm.nih.gov/protein/KAF7269990.1?report=genbank&log$=prottop&blast_rank=2&RID=NK1TDNZW016) | *Sitophilus oryzae* | 9e-07 | 30.7 |
| SvelOR8 | 320 | N | 3 | PF02949 | [XP_018567969.1](https://www.ncbi.nlm.nih.gov/protein/XP_018567969.1?report=genbank&log$=prottop&blast_rank=2&RID=NK1ZF9PD016) | *Anoplophora glabripennis* | 4e-55 | 37.2 |
| SvelOR9 | 377 | N | 5 | PF02949 | [XP_019759347.1](https://www.ncbi.nlm.nih.gov/protein/XP_019759347.1?report=genbank&log$=prottop&blast_rank=1&RID=NK22N2EV014) | *Dendroctonus ponderosae* | 2e-31 | 30.5 |
| SvelOR10 | 396 | Y | 5 | PF02949 | [XP_019765587.1](https://www.ncbi.nlm.nih.gov/protein/XP_019765587.1?report=genbank&log$=prottop&blast_rank=2&RID=NK2A2Y7R016) | *Dendroctonus ponderosae* | 4e-68 | 31.2 |
| SvelOR11 | 377 | N | 7 | PF02949 | [XP_019755672.1](https://www.ncbi.nlm.nih.gov/protein/1130214800?report=genbank&log$=protalign&blast_rank=1&RID=YX6ZTK7A014) | *Dendroctonus ponderosae* | 7e-143 | 65.0 |
| SvelOR12 | 393 | N | 6 | PF02949 | [QCS37751.1](https://www.ncbi.nlm.nih.gov/protein/QCS37751.1?report=genbank&log$=prottop&blast_rank=1&RID=NKVKU6DX014) | *Rhynchophorus ferrugineus* | 3e-119 | 47.5 |
| SvelOR13 | 368 | N | 7 | PF02949 | [XP_030746041.1](https://www.ncbi.nlm.nih.gov/protein/XP_030746041.1?report=genbank&log$=prottop&blast_rank=7&RID=NK2HDS09014) | *Sitophilus oryzae* | 5e-32 | 28.1 |
| SvelOR14 | 397 | Y | 5 | PF02949 | [XP_030746041.1](https://www.ncbi.nlm.nih.gov/protein/XP_030746041.1?report=genbank&log$=prottop&blast_rank=2&RID=NK2VPZNB014) | *Sitophilus oryzae* | 1e-90 | 39.7 |
| SvelOR15 | 263 | N | 4 | PF02949 | XP_018579026.2 | *Anoplophora glabripennis* | 2e-47 | 34.1 |
| SvelOR16 | 392 | Y | 6 | PF02949 | [XP_018564120.1](https://www.ncbi.nlm.nih.gov/protein/XP_018564120.1?report=genbank&log$=prottop&blast_rank=2&RID=NK336SZJ014) | *Anoplophora glabripennis* | 2e-54 | 29.7 |
| SvelOR17 | 396 | Y | 4 | PF02949 | [XP_019765587.1](https://www.ncbi.nlm.nih.gov/protein/XP_019765587.1?report=genbank&log$=prottop&blast_rank=2&RID=NK38BJCA014) | *Dendroctonus ponderosae* | 8e-66 | 31.9 |
| SvelOR18 | 430 | Y | 7 | PF02949 | [XP_019768310.1](https://www.ncbi.nlm.nih.gov/protein/1130256797?report=genbank&log$=protalign&blast_rank=1&RID=YX8G9MR7015) | *Dendroctonus ponderosae* | 0 | 63,0 |
| SvelOR19 | 346 | N | 3 | PF02949 | QCS37752.1 | *Rhynchophorus ferrugineus* | 4e-64 | 32.1 |
| SvelOR20 | 399 | N | 7 | PF02949 | [XP_030755395.1](https://www.ncbi.nlm.nih.gov/protein/XP_030755395.1?report=genbank&log$=prottop&blast_rank=2&RID=NK3JD6WJ014) | *Sitophilus oryzae* | 3e-115 | 46.8 |
| SvelOR21 | 430 | Y | 6 | PF02949 | [XP_019768012.1](https://www.ncbi.nlm.nih.gov/protein/1130255827?report=genbank&log$=protalign&blast_rank=1&RID=YX8HMSDK014) | *Dendroctonus ponderosae* | 0 | 62.0 |
| SvelOR22 | 385 | N | 4 | PF02949 | [QCS37751.1](https://www.ncbi.nlm.nih.gov/protein/QCS37751.1?report=genbank&log$=prottop&blast_rank=1&RID=NK4MUPBE016) | *Rhynchophorus ferrugineus* | 1e-70 | 34.8 |
| SvelOR23 | 402 | N | 4 | PF02949 | [XP_018579026.2](https://www.ncbi.nlm.nih.gov/protein/XP_018579026.2?report=genbank&log$=prottop&blast_rank=4&RID=NK4RZ3S1014) | *Anoplophora glabripennis* | 6e-47 | 29.1 |
| SvelOR24 | 393 | Y | 6 | PF02949 | [XP_019765879.1](https://www.ncbi.nlm.nih.gov/protein/1130248952?report=genbank&log$=protalign&blast_rank=1&RID=YXANZZHS015) | *Dendroctonus ponderosae* | 3e-115 | 47.3 |
| SvelOR25 | 387 | Y | 6 | PF02949 | [XP_030764608.1](https://www.ncbi.nlm.nih.gov/protein/XP_030764608.1?report=genbank&log$=prottop&blast_rank=2&RID=NK70G9B0014) | *Sitophilus oryzae* | 2e-41 | 30.5 |
| SvelOR26 | 388 | Y | 6 | PF02949 | [QCS37751.1](https://www.ncbi.nlm.nih.gov/protein/QCS37751.1?report=genbank&log$=prottop&blast_rank=1&RID=NK768G0Y016) | *Rhynchophorus ferrugineus* | 2e-141 | 53.6 |
| SvelOR27 | 409 | N | 4 | PF02949 | XP_030746041.1 | *Sitophilus oryzae* | 1e-61 | 32.1 |
| SvelOR28 | 395 | Y | 7 | PF02949 | [XP_030746041.1](https://www.ncbi.nlm.nih.gov/protein/XP_030746041.1?report=genbank&log$=prottop&blast_rank=4&RID=NKSD1U0R014) | *Sitophilus oryzae* | 3e-58 | 33.9 |
| SvelOR29 | 407 | Y | 6 | PF02949 | [XP_030764608.1](https://www.ncbi.nlm.nih.gov/protein/XP_030764608.1?report=genbank&log$=prottop&blast_rank=3&RID=NKJYS464016) | *Sitophilus oryzae* | 6e-74 | 38.9 |
| SvelOR30 | 379 | Y | 5 | PF02949 | [QCS37751.1](https://www.ncbi.nlm.nih.gov/protein/QCS37751.1?report=genbank&log$=prottop&blast_rank=1&RID=NKK30GRP014) | *Rhynchophorus ferrugineus* | 3e-67 | 34.9 |
| SvelOR31 | 298 | N | 4 | PF02949 | [XP_030767718.1](https://www.ncbi.nlm.nih.gov/protein/XP_030767718.1?report=genbank&log$=prottop&blast_rank=2&RID=NKK8EPYX014) | *Sitophilus oryzae* | 3e-86 | 63.3 |
| SvelOR32 | 384 | Y | 5 | PF02949 | [QCS37751.1](https://www.ncbi.nlm.nih.gov/protein/QCS37751.1?report=genbank&log$=prottop&blast_rank=1&RID=NKS70CDB014) | *Rhynchophorus ferrugineus* | 3e-103 | 44.2 |
| SvelOR33 | 401 | Y | 6 | PF02949 | [AUF73039.1](https://www.ncbi.nlm.nih.gov/protein/AUF73039.1?report=genbank&log$=prottop&blast_rank=2&RID=NKS7N43P016) | *Anoplophora chinensis* | 1e-81 | 38.4 |
| SvelOR34 | 359 | Y | 4 | PF02949 | [XP_019765879.1](https://www.ncbi.nlm.nih.gov/protein/1130248952?report=genbank&log$=protalign&blast_rank=1&RID=YXCBFXMK015) | *Dendroctonus ponderosae* | 3e-109 | 46.6 |
| SvelOR35 | 228 | N | 2 | PF02949 | [XP_028146887.1](https://www.ncbi.nlm.nih.gov/protein/XP_028146887.1?report=genbank&log$=prottop&blast_rank=3&RID=NKSKP7S3014) | *Diabrotica virgifera virgifera* | 3e-48 | 39.2 |
| SvelOR36 | 408 | Y | 5 | PF02949 | XP_030746041.1 | *Sitophilus oryzae* | 6e-98 | 43.2 |
| SvelOR37 | 388 | Y | 6 | PF02949 | XP_019755730.1 | *Dendroctonus ponderosae* | 1e-44 | 32.7 |
| SvelOR38 | 410 | Y | 5 | PF02949 | [XP_030767718.1](https://www.ncbi.nlm.nih.gov/protein/XP_030767718.1?report=genbank&log$=prottop&blast_rank=2&RID=NKSY96T3016) | *Sitophilus oryzae* | 5e-120 | 55.7 |
| SvelOR39 | 403 | Y | 6 | PF02949 | [XP_019771464.1](https://www.ncbi.nlm.nih.gov/protein/1130266964?report=genbank&log$=protalign&blast_rank=1&RID=YXCD8D55015) | *Dendroctonus ponderosae* | 0 | 67.0 |
| SvelOR40 | 413 | Y | 6 | PF02949 | XP_023310034.1 | *Anoplophora glabripennis* | 2e-96 | 38.8 |
| SvelOR41 | 391 | Y | 6 | PF02949 | [QCS37751.1](https://www.ncbi.nlm.nih.gov/protein/QCS37751.1?report=genbank&log$=prottop&blast_rank=1&RID=NKTBR7FF01R) | *Rhynchophorus ferrugineus* | 2e-115 | 46.1 |
| SvelOR42 | 425 | Y | 8 | PF02949 | [KAF7274160.1](https://www.ncbi.nlm.nih.gov/protein/KAF7274160.1?report=genbank&log$=prottop&blast_rank=1&RID=NKTHE8KB01R) | *Dendroctonus ponderosae* | 1e-170 | 61.1 |
| SvelOR43 | 409 | Y | 6 | PF02949 | [XP_030759997.1](https://www.ncbi.nlm.nih.gov/protein/XP_030759997.1?report=genbank&log$=prottop&blast_rank=1&RID=NKTNP21H01R) | *Sitophilus oryzae* | 2e-170 | 59.0 |
| SvelOR44 | 204 | N | 0 | PF02949 | [XP_030746024.1](https://www.ncbi.nlm.nih.gov/protein/XP_030746024.1?report=genbank&log$=prottop&blast_rank=4&RID=NKVVJ8TF016) | *Sitophilus oryzae* | 2e-38 | 39.7 |
| SvelOR45 | 389 | Y | 5 | PF02949 | [XP_030764608.1](https://www.ncbi.nlm.nih.gov/protein/XP_030764608.1?report=genbank&log$=prottop&blast_rank=1&RID=NKVGEWGA014) | *Sitophilus oryzae* | 4e-83 | 39.0 |
| SvelOR46 | 304 | N | 4 | PF02949 | [XP_030759997.1](https://www.ncbi.nlm.nih.gov/protein/XP_030759997.1?report=genbank&log$=prottop&blast_rank=2&RID=NKUEVHR4014) | *Sitophilus oryzae* | 1e-56 | 35.0 |
| SvelOR47 | 399 | N | 6 | PF02949 | [XP_030746041.1](https://www.ncbi.nlm.nih.gov/protein/XP_030746041.1?report=genbank&log$=prottop&blast_rank=1&RID=NKUMBS4B014) | *Sitophilus oryzae* | 5e-93 | 38.9 |
| SvelOR48 | 409 | Y | 5 | PF02949 | [XP_030764608.1](https://www.ncbi.nlm.nih.gov/protein/XP_030764608.1?report=genbank&log$=prottop&blast_rank=1&RID=NKUX9MC301R) | *Sitophilus oryzae* | 2e-120 | 45.7 |
| SvelOR49 | 403 | Y | 5 | PF02949 | [XP_019759347.1](https://www.ncbi.nlm.nih.gov/protein/1130227439?report=genbank&log$=protalign&blast_rank=1&RID=YXX16HC8015) | *Dendroctonus ponderosae* | 7e-75 | 35.5 |
| SvelOR50 | 88 | N | 1 | PF02949 | [XP_030763857.1](https://www.ncbi.nlm.nih.gov/protein/XP_030763857.1?report=genbank&log$=prottop&blast_rank=1&RID=NPDGVXZ9014) | *Sitophilus oryzae* | 1e-31 | 60.9 |
| SvelOR51 | 74 | N | 0 | PF02949 | XP_019875388.1 | *Aethina tumida* | 1e-15 | 47.0 |
| SvelOR52 | 74 | N | 1 | PF02949 | [QDD67777.1](https://www.ncbi.nlm.nih.gov/protein/QDD67777.1?report=genbank&log$=prottop&blast_rank=1&RID=NPFE8APX014) | *Galeruca daurica* | 6e-45 | 97.3 |
| SvelOR53 | 100 | N | 1 | PF02949 | [AKK25156.1](https://www.ncbi.nlm.nih.gov/protein/AKK25156.1?report=genbank&log$=prottop&blast_rank=3&RID=NPFPCVVB016) | *Dendroctonus ponderosae* | 2e-28 | 53.1 |
| SvelOR54 | 67 | N | 1 | PF02949 | [XP_019771140.1](https://www.ncbi.nlm.nih.gov/protein/1130201525?report=genbank&log$=protalign&blast_rank=1&RID=YYB5RCNF015) | *Dendroctonus ponderosae* | 2e-19 | 57.6 |
| SvelOR55 | 124 | N | 2 | PF02949 | [XP_030750615.1](https://www.ncbi.nlm.nih.gov/protein/XP_030750615.1?report=genbank&log$=prottop&blast_rank=2&RID=NPBPA5VV014) | *Sitophilus oryzae* | 5e-34 | 47.0 |
| SvelOR56 | 133 | N | 3 | PF02949 | [QCS37751.1](https://www.ncbi.nlm.nih.gov/protein/QCS37751.1?report=genbank&log$=prottop&blast_rank=2&RID=NPBSB34J014) | *Rhynchophoru ferrugineus* | 4e-47 | 56.7 |
| SvelOR57 | 129 | N | 2 | PF02949 | [XP_023024059.1](https://www.ncbi.nlm.nih.gov/protein/XP_023024059.1?report=genbank&log$=prottop&blast_rank=3&RID=NPCKZU47014) | *Leptinotarsa decemlineata* | 6e-27 | 43.1 |
| SvelOR58 | 148 | N | 2 | PF02949 | EFA01420.1 | *Tribolium castaneum* | 4e-06 | 30.1 |
| SvelOR59 | 124 | N | 3 | PF02949 | [QCS37751.1](https://www.ncbi.nlm.nih.gov/protein/QCS37751.1?report=genbank&log$=prottop&blast_rank=3&RID=NPFVNSG201R) | *Rhynchophorus ferrugineus* | 4e-19 | 38.5 |
| SvelOR60 | 103 | N | 1 | PF02949 | [XP_030748219.1](https://www.ncbi.nlm.nih.gov/protein/XP_030748219.1?report=genbank&log$=prottop&blast_rank=1&RID=NPD6X9FF014) | *Sitophilus oryzae* | 2e-30 | 49.0 |
| SvelOR61 | 114 | N | 1 | PF02949 | [XP_030754409.1](https://www.ncbi.nlm.nih.gov/protein/XP_030754409.1?report=genbank&log$=prottop&blast_rank=2&RID=NPD6XNZX014) | *Sitophilus oryzae* | 3e-32 | 41.2 |
| SvelGR1 | 361 | N | 6 | PF08395 | [XP_019764596.1](https://www.ncbi.nlm.nih.gov/protein/XP_019764596.1?report=genbank&log$=prottop&blast_rank=1&RID=NEBTPA46014) | *Dendroctonus ponderosae* | 0 | 81.8 |
| SvelGR2 | 344 | N | 6 | PF08395 | XP_008191523.1 | *Tribolium castaneum* | 0 | 74.0 |
| SvelGR3 | 412 | Y | 8 | PF06151 | XP_023022940.1 | *Leptinotarsa decemlineata* | 4e-81 | 36.9 |
| SvelGR4 | 333 | N | 5 | PF08395 | [XP_023021809.1](https://www.ncbi.nlm.nih.gov/protein/XP_023021809.1?report=genbank&log$=prottop&blast_rank=4&RID=NDX25BG9016) | *Leptinotarsa decemlineata* | 2e-39 | 34.0 |
| SvelGR5 | 403 | Y | 7 | PF08395 | [XP_030759073.1](https://www.ncbi.nlm.nih.gov/protein/XP_030759073.1?report=genbank&log$=prottop&blast_rank=9&RID=NDX5Y4H0014) | *Sitophilus oryzae* | 2e-30 | 28.6 |
| SvelGR6 | 388 | Y | 5 | PF08395 | [XP_018567270.1](https://www.ncbi.nlm.nih.gov/protein/XP_018567270.1?report=genbank&log$=prottop&blast_rank=3&RID=NDXBFJ70014) | *Anoplophora glabripennis* | 6e-50 | 34.1 |
| SvelGR7 | 427 | Y | 7 | PF08395 | XP_008190649.2 | *Tribolium castaneum* | 8e-49 | 31.0 |
| SvelGR8 | 416 | Y | 7 | PF06151 | [XP_030761254.1](https://www.ncbi.nlm.nih.gov/protein/XP_030761254.1?report=genbank&log$=prottop&blast_rank=2&RID=NNM182CK016) | *Sitophilus oryzae* | 8e-132 | 52.4 |
| SvelGR9 | 140 | N | 3 | PF06151 | [XP_030761254.1](https://www.ncbi.nlm.nih.gov/protein/XP_030761254.1?report=genbank&log$=prottop&blast_rank=1&RID=NNM40M5U016) | *Sitophilus oryzae* | 4e-67 | 76.5 |
| SvelGR10 | 405 | Y | 6 | PF06151 | [XP_019763584.1](https://www.ncbi.nlm.nih.gov/protein/1130241002?report=genbank&log$=protalign&blast_rank=1&RID=YYHF7VAV015) | *Dendroctonus ponderosae* | 0 | 66.0 |
| SvelGR11 | 172 | N | 3 | PF06151 | [XP_030761238.1](https://www.ncbi.nlm.nih.gov/protein/XP_030761238.1?report=genbank&log$=prottop&blast_rank=1&RID=NEAEZF1A014) | *Sitophilus oryzae* | 3e-100 | 87.2 |
| SvelGR12 | 398 | N | 7 | PF06151 | [XP_019760210.1](https://www.ncbi.nlm.nih.gov/protein/XP_019760210.1?report=genbank&log$=prottop&blast_rank=2&RID=NDWMA15C016) | *Dendroctonus ponderosae* | 2e-114 | 54.3 |
| SvelGR13 | 138 | N | 2 | PF08395 | [XP_019760833.1](https://www.ncbi.nlm.nih.gov/protein/XP_019760833.1?report=genbank&log$=prottop&blast_rank=1&RID=NNMDD4T1014) | *Dendroctonus ponderosae* | 7e-60 | 67.2 |
| SvelGR14 | 417 | Y | 7 | PF08395 | [XP_030759602.1](https://www.ncbi.nlm.nih.gov/protein/XP_030759602.1?report=genbank&log$=prottop&blast_rank=1&RID=NDWR7VG101R) | *Sitophilus oryzae* | 1e-112 | 54.3 |
| SvelGR15 | 264 | Y | 4 | PF08395 | XP_008190649.2 | *Tribolium castaneum* | 3e-30 | 34.2 |
| SvelIR8a | 887 | Y | 3 | PF00060 | XP_019770830.1 | *Dendroctonus ponderosae* | 0 | 73.0 |
| SvelIR21a | 793 | N | 3 | PF00060 | [XP_019753472.1](https://www.ncbi.nlm.nih.gov/protein/1130207172?report=genbank&log$=protalign&blast_rank=1&RID=YYD3U455014) | *Dendroctonus ponderosae* | 0 | 68.0 |
| SvelIR60a | 295 | N | 3 | PF00060 | [APY22698.1](https://www.ncbi.nlm.nih.gov/protein/APY22698.1?report=genbank&log$=prottop&blast_rank=14&RID=NDVRW67G014) | *Cnaphalocrocis medinalis* | 1e-12 | 24.3 |
| SvelIR75a.1 | 207 | N | 1 | - | [AVH87301.1](https://www.ncbi.nlm.nih.gov/protein/AVH87301.1?report=genbank&log$=prottop&blast_rank=8&RID=NDVNG6U6016) | *Holotrichia parallela* | 8e-45 | 41.3 |
| SvelIR75a.2 | 426 | N | 3 | PF00060 | [ALR72537.1](https://www.ncbi.nlm.nih.gov/protein/ALR72537.1?report=genbank&log$=prottop&blast_rank=10&RID=NDUWY9XZ014) | *Colaphellus bowringi* | 8e-61 | 33.3 |
| SvelIR75b | 532 | N | 3 | PF00060 | [AUF73085.1](https://www.ncbi.nlm.nih.gov/protein/AUF73085.1?report=genbank&log$=prottop&blast_rank=10&RID=X0G7A50A013) | *Anoplophora chinensis* | 2e-94 | 33.3 |
| SvelSNMP1a | 567 | Y | 2 | PF01130 | [AGI05171.1](https://www.ncbi.nlm.nih.gov/protein/AGI05171.1?report=genbank&log$=prottop&blast_rank=2&RID=NJZZWCB1014) | *Dendroctonus ponderosae* | 0 | 68.8 |
| SvelSNMP1b | 534 | Y | 2 | PF01130 | [XP_019756753.1](https://www.ncbi.nlm.nih.gov/protein/XP_019756753.1?report=genbank&log$=prottop&blast_rank=2&RID=NJZNU9EK014) | *Dendroctonus ponderosae* | 0 | 60.8 |
| SvelSNMP2 | 530 | Y | 2 | PF01130 | [XP_019770844.1](https://www.ncbi.nlm.nih.gov/protein/XP_019770844.1?report=genbank&log$=prottop&blast_rank=1&RID=NK0SWKYR016) | *Dendroctonus ponderosae* | 0 | 55.5 |

^a^transmembrane domain.
