# Supplementary material for: Candidate Chemosensory Genes Identified in the Adult Antennae of Sympiezomias velatus and Binding Property of Odorant-Binding Protein 15
Source: Front Physiol. 2022 May 31;13:907667. doi: 10.3389/fphys.2022.907667 (PMC9193972; doi:10.3389/fphys.2022.907667)
Supplement: Supplementary file 3 [file Table9.docx]

**Supplementary Material S1.** Predicted protein sequences for the candidate chemosensory genes in *S. velatus*

>SvelOBP1

MLLNSVLFVCALLFASVYNEIPEDYQIKSLQYLTECMKKWNINFETIARAVYGEDGFPED

KDFKEFSFCMGLKTGTINKNGTLHDDVIRDHLKMVVNSEKEIDRIMKKCMAQFGTPQEII

FHVTNCFHLEGVSL*

>SvelOBP2

MKILIVLFALFVAILAQDPNDPHGIVEVHKKCQNASDTHLYDKFEMYEKLRNYQKVNYKE

NMEKHYICMVKGLNMMTADGRVNVEGVRTHVKHIIANEPLVEKIIRRCAVDESTPEKTAY

RVWFCLVKYHIIDHSLHHQSSESSESYSKSNEHFWGKWHH*

>SvelOBP3

MYSALIFGIILLVSNALCLSEEMQELANQLHNTCTGETGATDAMIEDAKKGNFAEDESFK

CYIKCLLSQMAIIDDDGVIDTEAMVAVLPEEVQDALTPTIIKCGSAKGANACDNAWLTHK

CYYKEDPEHYFLI*

>SvelOBP4

MHSCQNSYFVVVLFVLNVLISVNCQSDQAGGPPGKHPFHAASKEDFQKCLDETGLTIDDL

KSLHHDPSKEPTEKHLCFMKCTATSGGLISKDGTLMTDVIKQNAPPYLDETALNTAIKCL

EGVGKISTCSDMSKVFKCFPKPPNRPQ*

>SvelOBP5

MNYLLSLMVTSFLNVIVICELSPEIAEKMVAM

ENRCIQEEGATREMVDNAHDGIFANDPRLKNYFACITQLSGIIDNDGHIHADVFKAGMKL

LVDDEALIDTVVEKCFVEKATIQDTAFESAKCVFTELHN*

>SvelOBP6

MLRIILCCLIIMPFLSEAYVPNANYTIRDFCIEDSGVTQEIVQNLLYNPSDTLTEEENCY

LYCVFIESGFLNDEGNIMIRRFKLLKETEEPEIDLDCLATMERVEKCSDMDNLKSCNV*

>SvelOBP7

MVYKCVSICSFITIVACRFTPEDHKNIQRIEEICLKETEAPKELAQALYSGIRPDDPEFP

NYINCVAQQAGLTDDSGHVITDVFKEKMKFFLKADVAKIDSAVAKCLVEKATIQGNDYEP

TKCLFTELYI*

>SvelOBP8

MKMIKKLVFLFVIFFFGGILIKNASSRMTEKQVVAAVKLVRNMCMGKSKATPDEVDKMHQ

GNWDVDRNAMCYMWCSLNMYKLMDADNNFDRKSADVQLQQLPENMIGYVTKCIDQCEHAA

THLDDKCVAAWEYSKCMYFCDPEKYFLP*

>SvelOBP9

MRRALILLVFGFATCSKALECGIMNMNNDQFKKVMTECVRDNQTLNKILEMTSSDASDED

SSMSSDEEVPVTRGMLDTKNVKLYQSRRSKRSRGGRFNNDSPMSNVQRKYGGPIMTTTEE

STTLVEDQSEENTKINNISDIEDSCILQCVFEKLEMTDSNGVPEHKKFADVLLKSVSGRE

MRDFIQESTDECFQQMDQEDSENPCQYSTKLVTCLGEQGKSNCEDWPAGNIPLF*

>SvelOBP10

FVNEKGEGAPLTNEEKEKVETYHKICLKQSNVNEVLIEQSHKGIYNDDPNFKNYLFCFSQ

RIGFQDTNGAVRKDIIEKKLSNLIEDQRVISKLIAQCAVLKESPEDTAFNIAKCLRECSP

DIEFFKVAPEIVYLSNEQTQKYQAFHQECADQTNINIEDIDKVRYTAKLYEGVRYKDYIY

CMVRKQEILRENGTIDKEKCHDCMKQIIQDTNVVEKLCQKCLHEQDTPKNTAFHIIKCFY

ENSPVHAEIFRREIPVPDEIAKKIIENTKICLNETGVDKEIIFKAQKGIFTGDPKFKQFT

FCMSKKSGFINDDGDLQIQVIKVKTLLLLHDLKLVESLVDQCAVKRETPIETAFYTHMCF

KKKSPGHLSIPGFEKALSDNGQREKRNQVIKECSMETGVSNQAVLRARKGDFQDDPLLKE

YFFCVNKKTGAVNEKGEFNHDFIRNSLAEMFGEKQAEKIMEKCLKIRDTPINSSFEAFRC

YYTSNPEAANAYS*

>SvelOBP11

MINVLILLLCTYVALTSCELPPEERDKILGIKELCQKEFGVAEEVIQNAHAGNVSDDPNF

PEYFACVGKRVGVIDDAGHIHADEFKIIGRLFIDDQAKIDALVAKCSVEKATITETAMAL

AQCFF

>SvelOBP12

MESANETTSPLIPGKSQRRYKTKFLAIKLWCTEKRVSSLKDKANPIQDKWNRVH

QHCQSDPDLFVKDEIFEQLKRGEKPQLPPNFGRHADCMLIGLGLQNTRGHAVQSGIEEAV

RRTVSDPAKVNQIVNECKDTTGLPLFKCL

>SvelOBP13

MAVFDDSISVILFLLVSAVGITLADQRQKVIDFHADCLEEHGVETDLLHEALEGSPPEDV

DFYRHIFCVARKANVMDDDGIVNLDNFKYDMRDVIEDHNMENVYSIMKKCVIQREDIMTT

VREAVQCFMKEDHGL*

>SvelOBP14

MSKWMVLIGGIFFSACCFPKARALSDEMKELANMLHNTCVGETGVPEELIEKVNSEKAFA

DDERLKCYIKCLMAQMACIDDDGHIDEEATIAVLPEEMQDEAAPVIRACGTKIGSSPCEN

AWLTHKCYAEMKPNAYMLI*

>SvelOBP15

MVTKKSKMCQVYCCIALVFCATSVLAKVDESIFTDDLKKLMDNLHETCVQKTRVSEALID

KVKEADFVDDDRLKCYVKCVMTESGTMDESGEIDYQALMEVVPDKYKISIKKDFENCSRK

ADGIDDLCEKAYTILQCVQEIDPDNYYFV*

>SvelOBP16

MKSFAFLVVFGLAIASVLALTKEEKEAAAAKFKTAKEECDSDPKTELHRE

DIEKWRKDGGKKPANLGAHALCILKKLNWLDANGKVNEAFIRGKAEENNVPADKIEKFMT

KCAEYKDEPEMLAEHIMTHVFIKARQHE

>SvelOBP17

MIKYVMLFAVIFVATRGDEFLTKEEIEKMKQVHEDCMKESKVDAALLQQAAKGEYSEDAK

LKNHLLCSLKKIDFQNDKGDLVPEQIAKKLTEHFKNEQVVETVVKECAVNKDTPQQTAFE

ASKCLHKFAPPDLDLTKYLF*

>SvelOBP18

MNNKVTVFVTCAFLVIVFVNGDLHSEIKEKMSEANEKCMKESGASEDEVSDFHNTGLFSD

TRELKAFIVCGAEITEIIDKDGNIDGEKMKEILSKFGVDEVKVNAILEKCAVKKDTVEDT

AVESAKCAHSIIHA*

>SvelOBP19

MNGLAVLFVCALFTVILCDLPEETKNVRTKCIKESGATEEMVSHAYVGNFSDDDELKEYF

VCVSKDGGFMDDDGHIDSDKLKEKLLKAGYDEDKVESVLNKCLVKKSSVQETAFEGAKCT

FAQLKK*

>SvelOBP20

MEKITFLFLISFCIFEIVQLAPVQYKKLSKKELNQIALSCIDEVNIDRSVIENVLKTEIL

PRNDQKYKKYLACSYKKQGYLSPDGKEFQYDNLYTFLQEFYDASDLEIIDRCKLIKEEDE

GELCFQNLECILNGLHKLEGVTEIETNDIPLNDE*

>SvelOBP21

MLKVTVFLICALTFTSVSTRFTPQDVTKFMTLASQCMQEWGIDSQILFDSFLSGKFPDDQ

KFQEFGLCIGKKTGTMDENGILQKDVIRQYMKMVIEDEKEIDRLMDKCVIQRDSPQETAH

QMANCFRSEYNFSF*

>SvelOBP22

MENLKIFSAIFFVLSLTAVQVLSDDGHSPFPMGEKHMEIAEKCEKDVGLTKEKVKELAEA

HHKNPDAPLNKDFACFIKCMATELEFITKDGKINTDMIKDKISPKFADKADEAIKCLEKI

DKISACEDVEKIMKCIPKPPM*

>SvelOBP23

MNKFSLVFVCAFVAIVSTNLTPEIREKLVRIQSECMQETGATDEMVMKAFAGVFSDAPEL

KEQLVCCAKKTGIMDDEGHYHKDVFKPKLMMVIEDEAKATSIVDECLVDMATPQESAFHT

AQCLYKELMQ*

>SvelOBP24

MKIFVVLSCLFVFANALDNALVDQFMQKVQEYGMACAEQEKPPQSDIEALLKKEEPQTHE

GKCVIFCVMKKFNIMVDEKTFGEGNKEWLDKAKTDDPEFYDKLMKAYKICDEKIERSDDG

CENALSMATCSKEEGLKLGMDKYLQQF*

>SvelOBP25

MNNLLKLSIVLVVISAVSCQDFTEEQRKRIIKNRQECIDETKVDPELIEKADLGEFAEDE

ALKCFTKCFYQKAGFVNEKGEVQKDVVEAKLPPQADKKKALEIVDKCQKKGKNACETVFL

IHKCYFEHTHQPAEPAAKSNEKTSSEEKKA*

>SvelOBP26

MKVLILAFFAVIALTQVSALTDKQKELLKQHYQQCKEQSKVDDAILEKARKGDFANDEKL

KNHILCVTKKIGFQNEAGQFNKDVIKTKLKEAVKNDETKAKNLMESCAIAHSDPRIQAFN

AFRCIWEKEKINLL*

>SvelOBP27

PENLGKHFICMVKGLNLLKEDGKVNEEGVKTHIQHVITKDEVVAEITKECAQDKETSEKT

AGHLWKCLQKHSVFQGSHGHDHGHDHGHGHSHESSEESDEHHHQHKHEHH*

>SvelOBP28

MHFSTHFVLACLCAFSLFKLVTPIHPVKDFFDNLPKADFEKCAKESKINLEELKNGSTET

EVSHEMLCFIKCASSAIGLIKSNGEFNTELLKDMDIKEDLEKVEKCIKNIGPIKTCDDIN

KLDACLPE*

>SvelOBP29

MNNLVTLCAVLATAVYFVKAYDFQDSEFNQILSLDFEDAVDSLGVIPLHPRSRRDE

EAAMGMDEKCRKRHRRPKLCCADDVFDKQHDKDKELFRSCFKEVVGIDKHDYHHKKFDLF

SCEEVEKRKNDMICVQQCVGKKKGLLNEDGELKEESIKLFLKENFAKEAWFGDVADKILS

TCLKEAKNATEHQVKPTSDDVKTCNPAGMAIKHCLFKEIQLSCPAEQIKDTNACDKFKDR

IKKGLDVFGHPPPFEGPKDK*

>SvelOBP30

THSNFKYVLNKIITMKCIVFLCVILLVFSVVVGKKHKNNNNEEKEKSMKKVFKECQRNDE

THVDGNILKKIRRQKQVELPENYGEHKLCLLKGVGVLNEDNSVNTENLKKRVTKK

>SvelOBP31

MFGVGKFLIVVLFVFEIEALQKVSNKCDIPATAPKRIEEVINTCQDEIKIAILSEALEAF

SVNEHKIGISRQKRSTFNEDEKKIAGCLLQCVYRKMKAVNEYGFPTVEGLVALYTEGVTQ

KDYVLATLQAVTKCLSKAQKIHGVSSPQLQASKSCDVAYDVFDCVSEEVAKYCGQTP*

>SvelOBP32

MKAVSLFLCALVVVALADPVQDRWNEVHKGCQSNPELYVDDAIFEAIKKGESPALPDNFA

KHAVCMLTDLDLQDEDGEVDEESVREAVEHSVNNRSSHDLIVKECSSIEKPTAEEVALAL

FGCFGKHHVNIGQF*

>SvelOBP33

MKIVILFCIVACVTCQFGGFKIDPAQMQKLITSNTECQRQTGAKQQDIMAMIQGKFSNDP

ALKAHILCLGQKFGFINENGQIQEDVVRENLSGYIPEEKIDTIFEKCVEQKDTPEDTAFH

AVKCLSDNRNMILN*

>SvelOBP34

MNRLLFLFPFCAVLSLATADAHMPLDIDAIKEKCQGETGITDEAISNALNYTGEFGDDIQ

VFFVCAFKEGGLMDDNGHLNIEKFKEQMTSFNVNKETENEIIKQCTEEKETIGETALATT

KCTYEVLKQGSK*

>SvelOBP35

MKQVLVFLVGVLFACVSCRTIPPELQEQYMAVRNKCLGESGATDAMVDSASEGKWGDEPE

LKKFFVCISKVTGVLDDSGNFNVDVLKSQLKMFSDSGDEAEINAAAEGCLVDKGNLEDTV

YESAKCVHQKVGF*

>SvelOBP36

AANAAAHFLCVSKTLGWQNEDGSINKELVQSRVIALFGKHRDTVDLIDKCTRPQDSPEAN

ARQLIICYKTMEGLL

>SvelOBP37

SKSGITEDDHQAYDISNKDQKMMCYMKCLMLESKWMKPSGEIDYAYIEEQAHPDVKDILM

AALNKCKVINDGADLCEKSYNFNLCLHEADPVNWFFV*

>SvelOBP38

IKTILIIIIIYTQSNKNASINTRYTIQNNCKKCGKHWEKSSDIRSKTLSYLEYLFRIVKI

TEDISYAFETSAKKLRIKNLILDAWNDVLLNKNMENGRSRAFKCFLHCLLTKYGWMDQDG

GFLLHDIRETLQQSDVEIPSLEYILYICTAVKSADRCHRAYYFTECFWNKMEEEQPSEDE

>SvelOBP39

MDANNNFDLSAIEVQLKTVPESMVEL

VDQSTRQCATAATHLDDKCIAAWEYTKCIHDYDPARYYLP*

>SvelOBP40

MKTFIAAAVIIGVSIFVNADLTDEQKQHLQADGKACIGETGVDPELVKKARNGDFSD

DPKLKAFTFCMSKKIGFQTDSGDFNTETIREKLAKAINDADAADNLIKKCLVKKGSPEDS

ATETFKCYYQSTPTHLTVF*

>SvelOBP41

RIPTTKKGICMIECIFNKIGVIQDGQFNRQGFVLAFTPATKGDVKKFVTLNNISKTCEGE

VAGNKKDCETTRMVLGCVAKHMDELA

>SvelCSP1

MWKVAATLVLFLGIGTLTKGEVTEKVKYTTKYDNIDINDVIKNERLLKNYVYCLLEKGSC

TPDGLELKRNMPDAIATDCSKCSVKQREGSEIIIRYLIDNKPEYWNPLEEKYDPTGSYKK

RYLDGKKEEVKVEPVEQPDE*

>SvelCSP2

MNLFGALFLLSILGFVLAAENPYTTKYDNIDIDRILNNDRVLTNYIKCIMDEGPCTPEGK

ELKKTLPDALSTGCLKCNEKQRSTTEKVVRHLMNKRARDWNRLTKKYDPQGVYKQKYEQE

IAKKTA*

>SvelCSP3

MKTIVVSIIVVVAIIGLTNARPDDQYTTKYDNIDLDEILKSERLLRNYLNCLLDKGKCTP

DGAELKKNLPDALDNGCSKCSEKQRNGAKKVIHYLIDNKRDLWNEVAAKYDPDGKYLKKY

EEEAKKENINL*

>SvelCSP4

MHQTKVIFCLVGVFITILSCQNGVVANSLRRVKKEVQVYTTRYDNIDVDSILASTRLLKN

YVNCLLEKGPCSPEGKTLKQYLPDAVATGCSKCSAAQKKIAGKILSVLLLKHRDDWDKLT

AKYDPDGTFRKMYFDEEDDYADLEEA*

>SvelCSP5

MKGVAIFVLFVLFGVVVCRPEEKYTTKYDNVDLDSIINNDRLLRSYIDCILEKKKCTKDG

EELKRHLPEAIKTDCAKCSEIQKNGARKMIRHLINNKRPWWNELQAKFDPQGTYVARHRE

EFKKEGITV*

>SvelCSP6

MVLLFTVLIGITLEFVGAMPADPEVKYYATKYDHIDVQAILNNRRMVNYYSACLLSKGPC

PPEGVEFKRILPEALQTNCGRCTEKQATVAFRAIKRLKKEYPKIWEQLSDKWDPDEIYVN

KFESTFGQNNGPMKVTSTKPTPTESSVDEQPLILNRFGGDDDEPTSNNIGGSSMLSSTTS

HQDPTTNKVITTTNSTPPSSTISTSTTTDYPYPPYEPSSLVSKTKTVTNPGLSTTINVSP

SDSSSTTFSSTTSYSTTSTATSPSTSPTPSATTSTTTNAPSTTESRTSKTTGSKSSTIQP

TIPGLIPINTFFTNPPIPIRPIINLNIGATVGGLVRGLGALGTRVMETGADIAEVVIKNF

ARPLRI*

>SvelCSP7

MKGLAVLVFVGLCGLVYCKPGDTYTTKYDNVDLNELVKNERLFKRYVDCLLDKGSCTKDA

QELKDNLGDALQNNCSKCSQRQHDGSRVIIRHLIKNKRESWNELQQKYDPNNVYINKYAD

ELKKEGITL*

>SvelCSP8

MLKVVFFLFAILFCVCAGQQYSTRFDQIDINTVLSNDRVLSNYVKCILDAGPCTAEGREL

KAHIPEALGTNCARCTESQKKIVRQGSNYLIKNRPDEWTKIAKKYDPEGKNAERFHEFLK

G*

>SvelCSP9

MKLCIFFLVTVIAIVLADNKYTTKYDNVDLDEIIKSDRLMKNYVNCLLEKGKCTPDGSEL

KKHLPDALHTECSKCSSTQKNGSKKIMRHLIDNKPEWWKDLEAKYDKEGSYKKKYREELK

KDGINLN*

>SvelCSP10 YGQSTENYRTTPMKALAILIFIVVTTVYCNPKPEDQYTSKYDNIDIDEILHSERLLKNYM

NCLLDKGRCTPAGLELKKNLPDAMENRCAKCNDFHKKTGEKVLRYLVESKRDYYDELEAK

YDPEKKYRKMYEEDLKKIGIKI*

>SvelCSP11

ILNMASSLLYFCVFFVVFGVTFGQGLNGGNVYVERQLMCALDKAPCDSLGKQIKDALPEI

IGKNCQRCDQKQLAILFALASCFWSHL

>SvelORco

MMNNFKVTGLVADLMPNIRLIQASGHFMLNYHTDSSGPVHLLRVAYCCMQLFFILIQFGA

IFGNLVAEKDNVNDLAANTITILFFTHCVTKFIYFALRSKLFYRTLGIWNQANSHPIFIE

SNNRYHALALKKMRTLLYIIIAVTLFSVSAWTGITFVGDSVHYIKDPDNENETIAEEIPR

LLVKSWYPFNAMCGMPYYAALVFQIYYVFFSLFHANLLDSLFCSWLIFACEQLQHLKEIM

KPLMELSASLDTYVPKSADLFKAPGSASLHDNLIENDYNTKNDELNLKGVYSTRQELGSL

NYRSGALQTFGQGGGGVGPNGLTKKQELMVRSAIKYWVERHKHVVRLVTAIGDAYGVALL

LHMLISTIMLTLLAYEATKIDGVNTYAASTLGYLLYALGQVFHFCIFGNRLIEESSSVME

AAYSCHWYDGSEEAKTFVQIVCQQCQKALSISGAKFFTISLDLFASVLGAVVTYFMVLVQ

LK*

>SvelOR1

MKRIYDICTFLEGEKFRLGVAGFYPSRIKRTFLVNLVTIMVYFITFSQMAAVTAYAVHST

DITKISEILLFLMTQIGFTNKLINFHLKTENVAGLDNIISEEIFIVVTKNELNIFEKYFK

RCQRVIHVFLVLCVATVSLYGIFPALDNTEPERKKYLFPGIFPFNPDNYYWAIYSAEVFS

VAVSAWNNAAMDALFTKHTVIATAFFSILVQKIEFLLDNDEGHLNDVVVEKRIRHSVIYY

NKIIRYISMIEHIFSYGIFIQFLCSAVVICLTGFQMMLVVSFKTVQFGLMVTYFSCMMFQ

VTLYCWYGHLLMEKSDSITKAWYTIKWDKMSIKNQKMLILIMERAKKPVAIRAAGVFKLN

LTTLMTILKNSYSFFAVLRQVYSK*

>SvelOR2

FAYNITGLIGEYQLKEDNRKNNASVESHYMYQLWLNGINKLEHLALVNTLSFTTSISSLI

YNITSTILLVMLLSCSASFLEVLQYRLKNYLGDNLEEDQMDKKLVDLKKMINEHIFLISF

TEHINECCKNIILMEFILTSVDVATGVVTLLKFHSNVGWFFFYEVMITMQLFALAWTANE

VNYQSQAIGDAAYESNWYLLNKEGRVLTQLMIQRAHQRPLLMTIGPFGPFTLQTFLMVMK

ISYTYMSVMK*

>SvelOR3

MSTTGENKLFKYWKILSIICGSWTEGFPGKSILWHRLYQLYAILNQVIFCLMVPSFWMKL

YELIQENRLSEAMENISKMIFVVLVQVKTIIQHSKKIKNLLKLAKEEESKIYARAEKQVT

RIYEEHVAYCKTVVLGLTYLTYISGSALVLVNMYEAYEYFKTVPPELRNVTDKPLPAPMW

YPFDTNKHYALPVLNQWFNVLQTLMYNSSIQALIHSVMIFIKAEFKILQYDLRNFGYHSA

DDQEKENGDNNLVILKSLLRKHQQLIEWVKDFDNSIKYLLLLEYSVTSMMLASTLIQLIL

RVKIFFNVPFLFLCTLQIYVLGWNANEIIVQSSTELCNSLYKSKWYGQNRDSKKIIHIMM

TRSQRPLHLSIGPFGDMNLQAAVSTTKLAYTILTVFIR*

>SvelOR4

MYQPKKNDAFYYSLTFMHYLHYYPSAEDEKSLRFYHVVSFLIRVFSCFVWVECAVHCTMS

IKNQVPVDISEDVVGLTGVGNSMLLCIFFELNVKNWSRLFCDITDTKRFGTPKNIVPVVK

KANLYALIYFVYCNIGVCIYGLVSILDTERCQRLNKEKGLHEICGTVTPLWWPNEEISSY

AKIITILCQVFSAVFYIPPSAILTFVPWEAAEVIIAKIHHLKELFANAFDTTEKDQRIKR

LKFCIRYHQEIIRMTQELNKATKKVCGQLSFVAAIILSCIGTQMLKEYSIGAMIHLMGYG

VAVFLVCQTGQKIRDETYDIQDAVYASKWYEVDPKLAKDTQLILLRCQKPLFMKAMPFGI

FNYNMFIIIIKTTYSYLTLINKTSEESMDI*

>SvelOR5

MQDSYLRSSTQTEKRILLFAGVYPEPQANLFTKFVRGFVFILLSCIMEFSLLV

LIFLNIKDLGTVMGALMLLVQQTAFLSKLSIFICKGKELELLDLRLKKFSIHKLQISDII

LLDYNLKHISKVGLIYRVSCILCCTFFGVGAFIQMCLKGSFDQLPMPGYFPYEITNIKRY

SATFVIQMASIYVAAALNSSMDVLNCRLITIATSLFQILTKRLNEIGCGDVRKVQAEFKQ

CVYFHTNILGYTQAIEDIYSYIVFSQFAASVIVICSTAFQLVILPLGSGEYIGSLLYLVN

MIVEVALYCYYGHNIQTMSESLGTACYMSRWYKTEPKVRKMIAIFMERTKRPVKLTAAKL

FPLTLTTLTMILRSSYSYFAVLQRVYSEK*

>SvelOR6

LIKDKKGLVRIAPSIALEFVVFQLVFKVFTYQKNKLPEICLAFMDMEKDIWKSSDQEVIT

TYKTVLQYNKYLNRGIIVSSIYTVASFAAISFSDLYATGMREWNFDTKPFMYDMYLPFNK

TNHFYFIIFCNVYAGFLGIILNIACQSTFYSLVIFAVSRLILLQIRAKKLDEFAKERNLD

IVMAVKQFVREHQDTIRFVKQLNESTKYVMLLEFIFTSVNMASLSFQIITVKSILELLYP

LCHFTLVFVQVFTLGWTANEISIQSMAIADAIYESPWY

>SvelOR7

PYKIYEFYIRFSYLCCTVAVLANFLVNFGVDNDRAIESMSMAINNLCTTLKIFLCINKNM

TNLLQKAIEDSKTIINGDPKIGKLVIGYVKYLNLLNIITTCYSFGITIHIATVGGPVEYY

KYVKMHLNSTEKPEHVLIMWFPFDMQKYFDLALICQIFLLVNSCVANYSSQALFNSLMIY

VIVKLKILQHYFRSFTTYLLLCGKTISKNQRATTNLKLFIKQHQDLISFVQKLD

>SvelOR8

YKNRHKFIELTRSISDFNLFEKPPGFEEFNKKMNFYSKIHLIYVLSGALSYFFFFAPLHT

RRCKILNLKENLSEVCELMTSIYIPYYQSTIFSSSTVLYSIVFLQFLIYLNIYTACGEII

WLNVECVEYIRFRIECIEYIRFRIGHLKSMLLNAFREENYVKRQKMFVHAVKYHNELLRM

GKLAEKFFGRELFLHVILTAAILGCCAFAMLETRSLDTLMVFVGWLNAVIMGCLSGQRLI

DESLTIPDVLYEVNWYNFDLKLKKEIVLFLLRCRKPMYIRAGNVVMTSNRITEILQTAYS

YFTLLSTLNKGNPDNSYEI*

>SvelOR9

MADQSYNLKLNFIPKLILIVTGAYSENFSHSKLFHIFFKAYSRCRLFLCYFIVFFHFFVC

IPTVAKSGSIQMLAMSITNGLALMTTAYRVSICLTEKSIKLVTKCFSQHQQTYGLHLDDN

IKNESKNNSKFFESFFNGITAITLAEGMVLNVCVTLFYFMHLDVKGSAYDMMPELFPFTS

YRGLGLIYKITAVLVSGPYIDLVFLCFFAPTMYVCGQLKTLQILIRNLESNGDSLKQIKS

IIWWHQDIIGFIEELNNLVRFPILLEYIATVLMLAAPMVILLLPNNTLYFVMDSVGLFCL

HTSKLFLISWQANEVQVQSTAMLDALAGSKWYEQNKEIKQIIHIMMLRTRRPLTINVGPF

FPMTLNSAVQTMKLAYS

>SvelOR10

MEIKNRDEVVSIAKVLLILSGMWPRKLENPYLHKIYKIYEKFVRLDYFIFYVLLVGELLR

LIVQQYTLTEIISSFTVACTCTKISFKIIIYFKNHVMDLLAEVTQQEEDIWQENDEECIK

LFMGNINMGKLFRNFISVSTVFTVFAYNITGLYGEKSLRKFNLENNASLESHYMYQLWIN

GMNRLEHPTFQHVLSFGTSMSALIYNVISTVLLLTFLVYSASHLEVLQYRLKHYIPDDFA

DEQMDEKLAILVKLINQHRYLIRFTEHVNECCKNIIMLEFVLTSVDLATGAVTLIKGHSN

AGWTFFYEFMIAAQLLALAWSANEVNYQSQAVGDAAFESNWHLLNKQGKFLTRMMIQRSH

HRPLVMTIGPFGPFTLQTFLMVIKASYTYISVMKD*

>SvelOR11

FYTTLSLLTLYFWYPGQNGKNYKLFFFTVSGILRTISLLSGFGTLMHAILSVKNNTDVDI

SEDIGDLCGFAGCMTVGMSFMLDHDKWSNFFHNLLDFKRFGKPPKYEEIVKRGNFISLVC

ILYTIPGMLWYCLVSYLRIPECEKLNREKGLHEACGMVNPTWLPEKAMEGTSFYLCYFFQ

LTGIAVYVPASFAICVLPLEAVEVIVARINHLKMLFRDVFKADDQKLCIKQLQYCISYHQ

DIIRISNELSYLVKSSIGSLFFTAAFIIGSLGSQLLKESTPKAIIYVMGYVSALFFVCHA

GQRLIDESLDIASYAYDSDWYEMNAKIKKDVVFILKRCQKPICLAAPPSMGSAGYLLFCI

MIKTSYSYLTLLNQMV*

>SvelOR12

NFNNKAMSKVNNFSYFLRHFQYDLVKLKMFSAWINYDFKVMKYYKLYCAIATVFFVFTFN

LQLMIYWVSNSTSIQSFAAIGYLLMMCFLGGVKSLFIFLNRNEFEDLINQLNNELFKPQN

EDELIHTEEVFAAYKKIKNILVTCSLTATASSLFTPFVYQKGDGFPSTAWYPMDVSHAPL

FQIAYIHQCISIIYLAGLLIYTDATVGRLFTFIGLQCDLLGKRLEKLGSMSDEENQKGLA

ECVNHHWLILNFHSTTQQVFGVIYCGQIFASAAALCMDLFLLSLTTPNSFEFFYLITYQV

SVTNLMFVPCYFASVMTDKSENLLNSAYNSQWINANKSFRKELLVFMGRSFFPLKFFAAN

YFHISVDIFVKVIRASLSYYAVLRNLNTTEDE*

>SvelOR13

FSTKVSGKQLLLYQIYSKFVSIVFMSSFLSCGMNFLDALLIKGNKTKAYEQLSYFSFLAL

NLLKSRICQKRQMLVLIQEGIYQSNEESRQDLKVKRIILEHLKYCNFLTISLLIYSWLII

LAFSAQGILELINFRKEHGYDNQPVHLLLIWYPFNVTHHFTTAFISELCIRPFLATYNSV

TQAIFITVMVNCIAQLKVLQYYFSNFHRFNGYLASHNIALENLKKCIRKHQKMIRYVKLL

NESTKYIILIEYSITSVMIACVVLQILKKINVFFNVGYLFMLTIQLIALCRNADLIRLES

MNILSAVYYSRWYEYNKDIKRIILFVMIIAKKPLTLTIGPFGPMTSGAVGSRLKLAYSYL

SVMTKGN*

>SvelOR14

MTSDKELFRLCKILMIFAGLWSQKYAAPIYQTLYKIFATSSQIIYTSSIILFAVELYRLI

STRNINGALENVSRMIFVILVSCKLRLCQSKKIIKLLELADKEEKHINSSSDKGTLNIYL

SHVKYCRSVVILLTISTLNAGTIFIGDGLYNTYVFFKEHSEEEWKLQEKPFPLPLWYPFN

KNKYYFLAMFQQCSNISQSMAFNIAIQALVHSVMVFLRAELKILQHNVRNLNLYSETETD

CAEGRDRIFDTLKAAVKKHQELIRWVEQFNDSIKYLLLLEYCVTSLMLASTLIQIFQKTR

VIFNIFFLFLCLLQIFLLAWNANEILLGSSVGLHDALYRSRWYEQSTKSKILIFFMMMRC

RRPLALTIGPFGPMTVDAAVSRVKLAYTFLSVMSTN*

>SvelOR15

IQANMYQPKKNDGFYYTLTFMHILHFYPRKKNEKNLKLYRSVSVLLRIISTFAWLNCITH

CIMALKNRIPIDISEDVAVLTAVGNSILVCVFFELYNEEWSSLFRDITDTKKFGIPPKIK

QKINRMNLYALLYFLYCNCGVCIYGAVAILDTGHCERLNKEKGLNEICGTFTSTWWPYDE

ISTFLTILINIGQIFTAVFYVPPSAVLTFMPLEAAEIIISKIHHLKELFANAFNVEDKHL

RTQRLKFCIRYHQEIIRLTEELN

>SvelOR16

MVNDRVFSFPTTYSFDKKLMMYVGIFPEPPSVNKYRLLRWCRLI

VTMLIIISLSLSNIIFIAMNITDTVKMIETLFICASGIVVLVKITILILNKEQLPYLESV

LQNEVLYSTSNSNIRMIKIKVEETRKYMWMYKFIVWLSTVFFFTVPLVDDKNKYRLPIAS

YYPWNIENNLNYFLTFSHQVISLSLGATVGATVDVLFAKFSNAAYISFKILNNHLKYIDF

SQDSSKVKKKLLENIKLHLEILKITKTIEYVFCYVILIQFASNVFTICFSIFLMTTGSLM

NYSSVYFLLAYWCNCFLYFLCMLIETVIYCAYGQKVMNESSTIHEALYLSKWYVANIDIH

KVLMIIRERANRPVILTAGKLFPLTVETLAAVLRASYSFFAVMQRTK*

>SvelOR17

MATKNKDEIINIGKVSMILAGIWPRKMKNSLLHKISLVYGVIVKLDYLTFYILLIGELLR

LLVKGYEFSVVLSSFTVVITVTKISIILMIYYKKNIIGLMEEITRREAFIWKENDEKVIK

VFLKNINLGKLIRNVVALGTTLTVFAQNLIGIIGEQKLIKYNKQKNATVESHLMYQLWLN

GVNKLEHRILAETLSYVTSISGLIFNVTTTTILVMFLVCSVSYLEILQYRLRTYIPDDLK

ESEIDGKIYTLVKLINEHRYIIAFTKHINNCCKNIIMMEFVLTSLDVATGAITLIKVHGN

MGWLLFYEFMITAQIFCLAWTANEVNVQSQAVGDAAYESRWYLLNKKGKILTQLMIQQSH

HKPLTMDVGQFGPFTLETFLKIMRIAYAYISVIQD*

>SvelOR18

MGLGKISPEYPKEDFFFAFDTAKFICGHATLPKDTFLIIRLLYKLYNFSLFSLAIIFIFC

ELIVFKKSLGDTQTMVSQIGMMFTHLVGLGKLWILVGKREHIDKIKDKLQDKQFQYKPID

DFQPGIRIRKEKFISVFIATFIFCLYSFVGISAHISTVLIVRKNTIGEQFIGNTSCETFL

PYYYYYPFDVSSKSGCFYSLDFMDICLDIYAWYIATLDMIFVTVLHLLKTQLNILGEALR

TIRKRCLDRLGLERDFAVLHDKEYPVLEREMYRELTHCTKHLMSLLDVRNDIEETFTFIT

LAQTVASLLIFASCLFVAARVPMTSPNFFSQLEYFSAVLSQFSVYCWFGAQITMACKAIG

SALYESDWFSCSERYKKSMVLTMTRVQRPLYLSIGKFTPLALTTFLAVIKGSFSYFTVFQ

SVGTVEQLE*

>SvelOR19

MILGGMWKLYITDNPFLKKLYFLYSIFIRGNITVYFILITMEIIRIVKEKEDAETIFLTF

GIFVNSIKIITKIAVYQREKIPTMFRQIIKEEKAARESDDPDIVKAYMEHVNYHEILTQA

QAVLTIAVVGSFLVNGVTLNIEQDRIERETNQTVEVPFMYQFYFPNKRQHLASVYTINVI

FGYACIIYNCVGYTILTACLVFSATQLKILQIKLTKIRKISQEFHGDDVVVTLKKFIKQH

KFHIGFVEKLNNSTKNLILMEFLLSSIDCASVATQVIKSSGIADALYYSEWHLCDREVKK

LMLIFMTRAQKPLIMTIGPFGEMTTASTLMIMKAAYSYVTIMKNT*

>SvelOR20

NKMRFDLTSIFTKNRQIFLYFGFWKVDTNCKYKTRYYLYTCFCFAIMIFFLLPQIIYMFV

NGNDVLEVTGTMYVFCTFFVNLVKTIGLYPKMDDIKKLLRNFGENPLLQVKCRKHLTIAR

HIKKICDYLFFYGSFLGAGTQVFWTLVPFLQEQRSLPTKGWYPYDAMSSPYFEATNFFQN

FASAFNILITMNIDSFTINLMMEVGMECDFLKTTLEHLTMFSSVNGSLVLRTRKLNKVDT

DFSEEMTNNLKLCIKHFIEIKRIANTVEDIYRVSLLTLFLGGAFIFCTIFYQLLLIKTGS

PEFFYLMFFLFSMLTEQFIFCWFGNEMTYRSQRIFRAVYNIPSWTDCDLKFRKMFLLFMI

SVQKPIVVYAGHIIPLSVGVFINVLRTSYSYFTLLKKL*

>SvelOR21

MSIADMQAVENTDEYATDFFFVNRWMLRCAGLWKPDSKNEKIQLLYKIYAVVVFLFVNLW

FTFTEFVSVFYTYKNESELIKNVNFFLTHFMGAVKVIFWYFKGNYLRGIMNSLEDSKLQY

ESYMQYKPGRISKEYKKLGIKYSLLFLSLAHATLTSSYVPPLITTVQYIKYEGTLNNSTV

SLPPRLPYYSWMPFPYNTGKMYLLAMAYQAGPMFSYAYSICGMDSLFMNIMNCIAGNVKI

IQGAFLTLRERCAIRLNNLSSTNTTIQEDEKMMAVMNFEMKKIIKHLQIVFKACEDLERV

HRYVTLCQVTATLFILCTCLYLVSMAPPLSKQFIAECVYMAAMFFQLYLYCSFGNEVTLK

FQELPYYIWDSDWFATDSTFKKNMIFTKMRAKRPVYFTAGKFSPLTLSTYMSIIKTSYSI

FALIKNTST*

>SvelOR22

NETMSQINFKQFFRIDYVLLRFSGSYLGEIHEIEPSWYKYYSFIVNAIMLSYNINVFGYL

LERGGNVKEIIAVGYEMAVATMTPVKIMLTSKYKKDLVELIEQYSNEYFQPKGEEEIEAV

NKKLKEFVAVRRFLGFVCWLWGGMLIIAPLGVELDQGIIFQGWFPWNEYTPLGHKIAYGY

QIFVVVTVACGTIYTDTYMAANCTFVGLQCTLLSIRLRNIGTSGTNPRKAFSYCMEHYRN

ILRIYEIIVKVYGHIYFCQLFSSTLSLCCTLFLISVAKTGSYEFFMHISGTCAIGFMLMV

PCWFASEMTTKSESIPHAAFHSNWPDTDRSLKKDIIFFILRTQQPLVLYGTWVFEISKTT

FVGVLRAAGSYYTVLHNLDLGDFD*

>SvelOR23

YVLHTIKIKKLKFQKKKMFFHPKKDESFYSILFFFKIQMYYPTQNFEKVTVRFFLLLIIR

SLTVFNYVGIAIHLTMSIKNGSYPVIIEDIALGGGYVSTFIGALLFRKHHKQWAQFFSEL

TDFQKFEKPANYDPMVKIFNGLSIAGSIYVLIGIYLYARLPIVNHKPCERMNELHGLNEI

CGLIYPFWWPHELTSSQKLTINIFQTISLILSSTPSVQMIFIPVTAVELLKIRIEHFCSI

LSTVFDDASNEENVKLKLKYCIEYHQFLINLGQNFNKLTLSTFSHIPAVASIVIAMILTQ

LLQVFNFGNVFHVSGWIFGIFLFCRSGQMLTDAFLDIAEAAYFTKWHETNVSNTKEILLL

MMRTRKQIFLEAVPCGTTNFELFVAVAKTTYSYLTLMTQYK*

>SvelOR24

MKVDFRINKSLYKLSVLFMVAIGLWKLPITKNKFLQMLYSLYSVTVQFMYILVNILQIIR

LIQHITEGTTPKIFYSAFTLTILVVEINVKMIIYLKNGIPFMMPDVIEREKNILENENEE

IRASYLKQAKFYLTISTPQCCMSFSGISWFMFFNFYKRAYDLFEPGEAFLYDLWFPFDKD

KHFYLVTFYNVFMGYNGFFFNCAILTTLQTLMIFAASQLRILQIRVRNVYSSKTTNQNDN

VAKTKELIKEHQFLIDFITKLNNAIKNITLMEFSLESINGAAGVLQLIMGVTAVEIAYSV

LYLVFISVNLLVMSWNCNEILEQSANVANAVYDSNWMDQGKSVKQLLQIMILRAQKPLSI

TIGPFGPMNNEIALMTIKGAYSYSSVMIQARH*

>SvelOR25

MLETYIKLFLIISGIWKGNWTENIFLQNVYIWISRIVLNLWILIYVLVLAKLDQLIHINA

DAEPIFFCVSILITYFNVGVKIFMYQKVKIWRLFRNIINEEQKIYATLNEELISSYFKTI

KVVSKYQKTANIFLFAALLGCSLTCFFQYLYDKDRVGKTENELIQRSIILPIWFPFKEND

HFVTVIGINILAFYVLGYFFCASHIIIAALMVFTVSQLKTIKVLLEQQDKNNNYYDGLKN

IIQKHYFIIEFVKELNDATRYILLMDFLLNSVNIAPIVFQITSKNSLKEMTIPFLYLVSL

IVQVFIPAWLADKIKEESLSIGYALYQTKWYDQDIKYRKMLLIILHRTQKPLTMLIGPFG

PLTVETILSVMQTTYSYITVMKNYSL*

>SvelOR26

MARSKYNYFQHFKDDLVRLKMFGGWINYKFKILKYYKAYYFTVNFLLIYLFNTLLVLYWI

LHSHSIKSFAAIGYISMICLMASVKSFFFYYYRDEFEELVIQMDEDLFQPKDEREEYIVK

KNFNFYWIIKTALVTVSFFSTICSFTVPLFYQKGDGLPTNAWYPVNVTSSPLFEIAYVHQ

SISIFYISVVNIYTDVIVAGFFTFTGFQCDILCERLKNLGRTSSYKNQIGLGKCVLHHWS

ILRFANITEKVFGKIYFAQIFASTIALCMSLFLLNLTEPKTFEFVYLVVFQFAIANLIFL

PCWFATELTRKSENISSAAYFCDWINTSNDFKKDLLFFITRCQRPIKVYAAILFEISLET

FVKVIRSSLSYYAVLKNLNMAEEELTN*

>SvelOR27

GEAAELSLHCNSKMGFENKMFRTYRQLAIESGSWVGGFPDKSRFVNYVYNFYALCTHGFF

ASMGPMFWIELYVLIDKNDISMATECTSKMVFVLLVQVKLILNRSDGLQKLLKSAMEEEK

RIYAEARKEVIKIYDDHVDICRKCVLFLTYLTYNAGTSLILVSWYDSYQYYQRVPPELRN

ITDKPFATSMAYPFDKHKYYGPAMVHQFFCVFISVLFNCAIQGLVHSVMIFIRSQFKILQ

YELTMVGNEIPEGHEKDIDDHNLKSLRTLLVKHMKLIDWVKDFNESIRFLLLLEYSVTSM

QLASTLIQLVLGAQVVFNVMFFFLCGMQIFVLGYNANEIILQSSTELCMAIYKSKWYEQT

KDSKTIIHTMMTRCQRPLHLSIGPFGDMNLQAAVSISKLAYTILTVVR*

>SvelOR28

MKSGENLFKVTKWLMLVAGTWKLELTMAPKIWKRLYNI

YSAVITICFYTSPLSITIRFCKLWGVDSDKAVECLSMLVIIFLLATKMALCQTKQMTTLL

TKAIDDVNTSSMTNDQYIKKEFSTTAKYTNFLNICITVYSFSVGLHLNIVGYFEYLEFKQ

QHPNNTENIDKPMCVQYWYPFDYNEHYGFVLFYQLASILFADLYNSAIQAIFNTMLIQLQ

VQLKVLQHNCRNFTKDIVTGRPVTKQKSFENVKLLVKKHQKIITYIEQLNKTLKYPILIE

YVVSSLMLASILVQILSGKKVFFNVQYFLILAYQWVILSWNANEISLQSQRMAYALYESL

WFKQSKETTQIIFIMVLRSRKPLSLEIGPFGPLTNDAAVSRLKLAYSYVSLMTGNG*

>SvelOR29

MSVARNFYYSELINTNTMENDRFIKVCKWFMIFSGIWNFDDESISPLAKRGYRFFARALS

GHYVLFCISLGIRIIFLIQEEQPPERISLAIAVFIIASDITVKILIYQVNKVPSLFKYVL

DMEKEVSSSTDEDIIKYYEQQIKTCKYYNIGLAIATLFSAGSYLVMDTIKQIKGLETIDE

YYFEIWLPLDKEKHRIFTYGINVSWGILSTLFNSTIRIALVTLVIFIETQLDILQIKLKK

FDGNGLGDQEKLAMIKDLIRKHRFLIWFVETLNEAVKFVMLMEFLLNSVNVAFLLFQLIT

IRASLMEVVFPVCYLNLIVVQIFTFAWTCNALKLKSLGISDAIYESKWYEQDENIKKLLH

IMIHRSQKPLTLTIGPFSPMTTETALSTLKAAYSYVMLMVNKYYRG*

>SvelOR30

MSKVDYKKFFRMDFFPLRFAGIWLSEEKEIKPKWWRSYSYLAFSVLLFYNMSLLIKWFLC

KNLKEAAFQGYVATITTMNPVKRMLLVSYGKDLNQLIKEFDNKYFQVTDDEERELVDEKL

GRHRVIIIAHQTIVWIWLALVIMTPVFYGSEDGLILPAWFPFPKEISPWFEIAYASQVIS

ILIAGTITIYTDVYMAANCTFIEAQCILLSKRLDRIGDHGLKNNEWFARCLQQYKEMLGF

YEITTKVFGMIYFWQLFSSATSMSMVLFLLSLAEPGSYEFVTLLSAVISVACILLVPCSY

AAEMTATSESISTAAFHSNWADSDDNVFKKDVMFFMTRSQQPLKFNARWVFEISKTTYVN

VLRAAMSYYTVLNNMQNA*

>SvelOR31

MRIYPKTEHLKIPMICSSSLGIFPWEFMFQENPSVQRFYAVFSKVMLSYYIFFLFTEYVQ

LYKLVSDYSPRMDEISGNLCITLIYTTTIIRQLIIKLNPGFKNIIRQIIDTEKVINAQED

KEVIKIYNETAKDTSSKCKMYLAILFVITMLYIFRPLVVHGYEKEIGFNQTTFVKALPLS

SWFPFDEQTLYLEAYLWHVLDSLVGASFVTYTDILMFSLIVYPTGQLKILQYILRNFEMY

RKRIQIQYRIQDDNEAAFITFRECIQEHKRIIRYVDYFNESMNALMVFDFLQSSLQIA

>SvelOR32

MSINAYSTHYKTLFKALKYVGTGVQYDKNEKTKLIPLYSAFINILFIMSFNTSIFMFWIK

NTNDLRSFADKGYLVPISVMIVMKSYYLFKNREILLHMFVYLDQDIFKPKDKVEHSIARK

MLREWRRIQTVLYGAGFAACTSLFISSKFQRREGEFPIELWFPFEINSTLYKVIYIYELI

SLVVSPSISILADLTLSGFCLFTGLLCDLLCHRLKLIGNISAKENREPFLECVKLHNSIL

GFLKNIETVFGKMYFGQIFASTTAMCMDLFLLSLLDPKSLKFVYHFVFLIAITLFLLVPC

WFATEMTIKSEHIPNAAYFCNWIDAPNNFKKEIIFFMVRSQRPLRLYAAYFFDISVETFV

KIVRAALSYFAVLQNLNMAEKEK*

>SvelOR33

MNSILYNSFSGHLALLRYFGAHPFPNWPIFWYRVYAFTSFAIITLPLPILNVAACYI

SVRANGDIQEVSAELFMTFQCIACIFKHFYFKIHPERIASTLEWLNKPIFNTYGKEQEET

VKATANFCRKIFRGYFTLAIVCYIVWISMPFFWEERSLSFKMLVPFDPFKSEVVYWICHL

FQLSVVTKGLWCNVAMDTMIPGLIWQAAGQIMVLKGTLRNIVKDADEYIVDVEKTGTFIT

EKEKLRQTIIYTKISKCVIHHNAIKDYVKELEDTYSSPLFFQLLTSVFVICVTLLELTII

NTSDVLFFELLSFLPAILIEIYLFCSCGQILFEESSSITKDMTTGDWLEMDTKSRKAVWL

IMEGSKKPLIIKAGGLIDLSLETFQMIMSRSYSLLAVLNNFDR*

>SvelOR34

MKSYFKVTKSLYKLSRLFMIASGLWKLPLTKNAFLQKVYSLYSVVVQVSYILYNISMIIR

LIQLMSEGITSQILYNSLTLTILISEINYKMVIYLKNGIPYRIPYVIERENIILRNESEE

IKESYLKQAKFYLTMSLCQCCMSFGGITWFIFFNFYKRLYKLFLSNEAFMYELWFPFNKE

KHNFFVTSYNVILAYQGFFFNCAILTTLQTLMIFASSQLRILQIRVKNAFDSRIDDDKDN

IIKTKKLIEEHQLLINFITSLNNSIKNITLLEFSLESINGAAGVLQLVTSCNVAQAVYEC

NWPDQRESVKKLLLIMLMRAQKPLSVTIGPFGPMNMEVALMTMKGTYSYASLMMQTRK*

>SvelOR35

FRILSPADVTIPLNYILVFILEMFTAVVVCFGVLGITMTISGFLIHIIAQIKNYRKVLLD

TFRSTYVPKNKDILETKRRMIFCIRYHQTIIRYTERVYAIFNYLLLMQVSLTSAIFGGIL

YLITSVQGTDKVKYILQLAGWNSLQLLTCYYGQCIITESTSIADAAYASLWYDGPLELQK

NIKFIIIKGQNPLTLRAASIGVISLETFVKVIKTAYSFFTLLITISD*

>SvelOR36

MTDKTKNGQLFKYCKLLMIIAGVWSKGLETPSGFKQKCYKFYSLGGQIYYVSCLILFIVE

LFMVTGVDMEATIANLKSLTFVTTIIAKMILCQTSKMVKLINSALAEETMINTSNNISIQ

MIYFEHVNYCRKMIIAIIVALYVAGITCLGEAFYESVQYYKEYNYWLSRNETVAADIYKP

HPVQFWFPFDKDAHYILAMTYECIHIIQSLLFNGAAQALVNSAMVFLRAELKILQHYLKN

YNEYPIEDRFLEIQDVSKRTIKVFLIKHQEVIRWVKDFNNSFQYILLLEYCIISLMLAAT

LIEMYQGEKIPFNTFFFILCSLQLFILAWNANEILMESSSGLLNSLYTSKWYQDESNEAK

VFIRIMMIRCFKPLAISIGPFGIMTMNAAVSRMKLAYSVLSLLSSQS*

>SvelOR37

MFVDTVKLFLNTLGIWSPQWTENKHLKMYRLIKYVLLGCSIFGLILMILKLIQMIKSNAD

DKIIFSSIAVLISAWALVAKLLIFLNNKSILVSLFQKVLNKDRKINLTNDNEIALLYFVA

VQFCRKIEAGIKMYLPPFLLVFFLTSFLKCFYETKSDTSETEFFLFPFWLPFKERENIIP

STLIKTIGLFVYAWLFGAFQTIYITVWTYAVVELKMLQTYIKRLDNYDDKQRKNSYYKVL

RKMIVHHLYVISFIRELNGSTKYIILTDFLLNSINVASVAIQILIVNSPKELLFLVMYLS

ILVVQIFIVGWLADEIKHQGLAVGETLYQTKWYEQDNKFKRAVQIILMRTQQPLTIAVGP

FGPMTLETALGTMRAAYSYVTIMKNFQ*

>SvelOR38

MTIYPKTKFLQIPMMCAASIGLFPKEFMFEKNIFLQKCYSIYSRFMFGLYIIHLITTSIQ

LVKMIIEGNSQVSEISANAPTTLIYTITLIRLLIIKHYSGFKYILKQIIDAENIINVQDD

EKVMDIYNKSVKDSSRKCLMYPLTIYLVTILYLSGPLFTPGFEKQVGNETVFIKVLPISV

WIPFNEQTYYLYAYLWQIIFSLIGAAFVSYTDILMFSLIVYPTWQLKILQHKLKNFEIYR

KRTKIQSNIKDDDVAAFLTFRECIIHHKNIIKFVDYFNASMRVIMVADFLQSSLQIATVL

TQVLGNELTVMLVICVGSYVTGMVQRLILYYYYANEVVVLSDGLANSLWDSNWYEHSSNV

KVMMLIFIIRAQRPLQLQIGPFTVMSLDSLIKILKATYSYITLIWGTNQ*

>SvelOR39

MIFPNNQILKISMYMSSTVGIWPIIFMENKRLKKIYDIFSSILYYYYLEYIIRAYYQLFR

LLTAEHLNVEEILGNLCITLIYTVSLFRLQAFSKKSVRTLFSNIIEEEENILNDPDPNIK

KIYLTSVKDNKIYNMLFLFNGWVVSVLYFVHPFFMELPTMVVNNETITLKTLPLSTWWPI

DIQKHFWIAYYWNVFDGTLGSSFVINSDILTFSLIVFALSQLRILGYRLRSFANGNTVTR

NPAFREKIHKREFIFLIKQHQKIISYMKTFNDTMKYVMLFDFLQCSVQLATITLQLLVME

INVQNVIFVGEFLLTMIIRLVIYYFNANEVIYQSQNLAIDIWKSNWYEQSNDVKKMMVFF

IARAQRPLKFQIGPFGAMSLETFITILKATYSYMMLFINTSG*

>SvelOR40

MSKNPSDKRKWILNTDSILYNSFTITLRPWQVYGMYPIDGTPQWFYYLHAGFLHCVFTLP

VPVLAFTYLFVNINSLDFFAVIDLLFMVSEIGINVLKYMPFRLYPEKVRHTVEWLTKKEF

TTCHPDHEHIAKSTSKICRQVYIYYASTATCTLLIWCFQPILIGEKKFPNVIWLPFDPYQ

SDYVFWFIYVMVMLGTSNSCFGNISMDCVTTGLIYHAAGQIRIIKAILKDVITHTMEKIS

KEHGNNLPPKEFDMLKNRYIYKEICQCVDRYNLVKEFAKDIEETYSFPIFCQFTASIFVL

CFACIQFFFVEMWSVNFFVTVLWTGTMTWEVFLYCYFGSLLHHESSTFVTDIYLTDWLDL

DQKCQKALITIIENAKKPLMIRAGKIVDLSLDTFATIMRRSYSLLAVLNKFT*

>SvelOR41

MPTNMATINFDYRKFYRNDLFVLKLGGVFPDENSGKYSKWHTAFTLIVNVFFLNIINAV

MAANWVIKTDGLRSFAALGYIILILMMGQVKSIFWYLNRKSYGRMLVMFDEVKMFQPKNE

AEMAILKENYKHYGKIRRLLLSCCWISSFSNLTTPLFSGDKNALPVPSWYPFDIYYSPVF

EIAYIHQAVSVIFISFLNINTDLTVAGFLTFIGLQCDLICYRLKQLGKQNGITKNDWLRC

ISHHKNIVRYLETTSQVYGKIYFAQIFASTTALCMDLFLLTLLNPSSFEFFYLLMYLIAV

ANLFLVPCWFATEMTRKSEAIPTAAYSCNWLETTTSFKKELTIFMVKTQRPLKLYAADYF

EISVEFFLKIARASLSYFAVLQNLNTPAQSQ*

>SvelOR42

MPAFCILILGACIDPYKGQQKIFTIFWKYKMAKNIKILNLHIRVLKLLFL

WPKDCLTKTQNLIINYGYFFLNMCCNIPVVGAVVYQLSVGIDNLNIFIEALIGVCDIIGY

MVVYISLLTNQEVIEKIIQDINIFLKFCDRKVIEDIDKVCIRYTKYLLIYVTIAVISNFG

WALMSTKHCTESKDEFYLKHDPCGMVTQHHYPFDASKPNIFWFLFVVEACYCYHICCEFS

MATATVVGLLMHVIAQLKNCGIRFFEVFDENDKNSSTTEETFITCVKYHQAILEYAERVC

NIFSNMLIIYVTMTSCVLAIISYQIASNSNMQDRIRYSMLLLGWVLLFFLICYYAQKLEI

EFISITDSVYNSNWQEVHVDTRIRKNIILVIARAQKPMTLKVKYMGNISLVRFVSVLKTA

YSFFTLLLTVTDDA*

>SvelOR43

MLTLCPDFRIIDANTQTQHMPLVRMTMLWLGIWPMKHGTTMELIYRTYFWASFTYYIIFD

ITGAMLAIQTWGENYLFTAGSMGVVIEYAISAYRVLIFKSKEFGKLIYELIEKEKEILAS

RDPAFIKIFKENATYTRRVVVFYICVGTTSISLYYITPLVSEIFLPLGINNATGIKEHHF

IVYSWFPFDPNRYYWAAYLIQFSGTLYGYGYIVYCGSFYISILTFVLTRMKILKHILINF

TKYYKKFKNECRLPEEQALQAFIKTIVMEHRSIIDFVERINKQMKQFMLMNFLISSFQLS

LLAYQLLILPPLKIVAVFFYFSTLTTQLFFVYHTGHDISLQSENIAESIFKSDWYIYPPK

VAKMLQVICIRAQKPLVLAIGPIAEVKVNNLFQIAKALYSYVCIFIRT*

>SvelOR44

LFYQFATVLYSDLYNSAIQALFNTIMIELSVQLKILQHNFRNFTIDIDTGKKVSQHRAVK

NIKDLVKKHQELMKFVGDLNQALKFPILIEYGVSSLMLASILVQILSEKKVLFNVQYFFI

LAFQWFILSWNANEIIIQSQHLPFALYESRWYGQNKETKQIIFIMALQCRRPLSFEIGPF

GPLTIESAVSRLKLTYSYVTLITG

>SvelOR45

MELTNIPKRLLKIGGMWPVDVDLNWKPETRKYYKIYQYFLATYFMTFTFMNGIACVNLVR

EHKQFERISTASGLLVAQIFMMFKVAVITKNKVIHRLLTFTDFEDKIWESSKNEINNYYT

KYCRYLYRCVVVLVWSANIFFCVIHIFGNGGIFSVGIENWNFDNGDILFETYFPFNKRKY

LLVVFGSSFFYLYIIGFCDSACITTLYANLVFALVRLKCLQIRLETLNTEKHNRNVIVFK

KLIQEHQDIIRFVETLNDSTKYILLMDFLLNSLKLAFDLLEILKVQSLSNLSFSIFYALY

LLQEIFFIGWLSNEIKMQSLAVADAAYKSLWYEQNQKTQRLLQILILRAQRPLTMTIGPF

QPMTTDTIVTTLKAAYSYCLLMMNQNEI*

>SvelOR46

MKLFQKKKKMLQSDYASHMSYGKIYMTVFGLWPVKYQGFKKKCYEFYYYTTFTYFCLFVV

TLGIEAFLSLKTSKEAFAGTAGVTIAYSICIFRVYIYRSDLLISIIHRMEKMERKVRNTN

VSSINKIYKNNSRISKKIELFLLGMAVVGAMSYYVGAIVKDKMAFRNATVIPERYLVIQS

WFPFDKNVHYWKAYSIQFVGCLYGTAYLVFCPAFYITMLTFIIGQFKIIQHHLNNFCLMS

NRDEIKDNLNIEEKQNIFLKEIIMDHQCILKLVHDMDKCMKTLTLLDFAITSFQLAMVVY

QILH

>SvelOR47

DFEFCLASVLLNIRILLRNNMAPKDKMFKIARFLLLCNGMWTQELPPWPKICQIIYRVFS

SFSQGLYISCLILFAVELYRLSKVDINGAISLLKSMMFISVIIAKMLMVQTKKIAKITKL

LMEADKQIIALKDRAVIKIYNDNVKYCHGLLYVVLSSIYSCASIYIGGALYESYEFYATH

PFSSETTEKPHPVQFWFPFDKNKYYKVSMIYQIIHIAHAGLYNSTVQALVTSVTIFLRTE

LQIQQHYLRNIRPYKAANKLTHNMKEIPYIVLKTCIKEHQKMIRWVQDFNESFQYILFLE

YLITSFILAFILMDILNRPDKAFNVTFFTFVTLQFFTLAWNANEIIIESSAGLSDALYES

SWYTMDRQSQILIQIMMIRCRNPLALTIGPFGQMTVNTA

>SvelOR48

MKNFEEETPRVKLTSLEKNGLLYVPRIFMQIWGMWPLTLFENRATRRWYSVYRIFTLGFY

STFFILLAIECANLTIHKKGFERIARSAAIVFIILVLNFKAIIYQKNEIPKMCLAFMKVE

KDIWRSNDKEVQDLYRTILQYNKYVNSGVIVSSIYTVVSFSMMSILELFNTNIKDWNFDE

KSFIYELYLPFNKTNHFYFILFFNIYAGFLGIVLNIACQSMFYSLIIFAVSRLIILQIRT

RKFDKFAKDRNEDIVLTIKGFIREHQETIRFVEQLNESTKYVMLLEFILNSLNMASFSFQ

IITIQSPVKLIYPLCHFSLVFFQVFILGWTANEIQIQSLALADAIYESPWYTQNKAAKQM

LFIMILRAQKPLTLTIGPFTPMTIDTAIGTVKAAYSYVTLMTNSYNIE*

>SvelOR49

MPSKTMKLARILKLLMVFTGSWRIKISNTKLMQVSYNVYSAVFQITFVYFPLALILNLPT

LIKSKKFELIAENVGYTTITLVMLAKLVIIQTGKVQSLIKQCILEEKRTYMEESAQIKDN

FDLHVQHLNNIYYVLIPITISVTSTFSVANYADYAAYKDFNSTVVKPLPLPIWYPFDTNY

YYTTAFWLAIFITLVGTLYNVVTQALFLTLTTCVALQLKALKIYAKYFHLYSKHSNLSSG

EYNEVDILRVFRNISIKHQNVISFVNDLNIAMRNALFLEYLLNSLNIASLMIRISMMSNT

RTMLLFCILMLTHQTLQLFWMSWHANEIEIQSVSLASALYESKWYEQSQKVKKIVHIMMM

RSRKPFQIFIGPLFPMSIRTGVATLKAAYSYVTMIFTLAAGE*

>SvelOR50

KTFEFFYYTVYLICMSNLLLIPCWFASQMTEKSEGISVAAYNCDWVNSNDSFKKEITFFI

HQSQQPLKLYAADFFEISVEIYIKILRA

>SvelOR51

NEIMDQSSSLGDALYQTNWYVQDLRVKKLLPTVLLHIQQPLIISNGPFGPMTLETALKVL

KAAYSYIMLMKNFR

>SvelOR52

GNRLIEESSSVMEAAYSCHWYDGSEEAKTFVQIVCQQCQKALSISGAKFFTISLDLFASV

LGATVTYFMVLVQL

>SvelOR53

VKVVGDLVFLLFYFSLMVLQIFFLGWTTNEIKIQSSSISNALYESKWHMFDKKTTKLLQI

MMMRAQQPLVMRIGPFGPITTETPLLIMKAAYSYVTIMKN

>SvelOR54

QSQAVGDAAYESKWYLLNKRGRFLTQIMIQRSHHRPLVMTIGPFGPFTLQTFLMVMKISY

TYISVMK

>SvelOR55

MLIDFLLNSISVASVAVQILVVSNYKELLFLLIYLGLLLTQIFFIGWLADEIRNKSLAVA

DVLYSTKWNSYDIRAKKLIPLILLRAQHPLTMAIGPFGPMTLETALKTMKAAYSYITLMK

NFE*

>SvelOR56

YIHQSISLIYLSGLLIYTDVLVARLFTFIGLQCDLVCKRLEKLGTMSDGDNQKDLVQCVH

HHWFILKFLSVTQQVFGVIYCGQIFVSSAALCMALFLLSLTKADSFEFLYLIIYQVAVAN

LMFVPCYFASVMT

>SvelOR57

DDNMKYLMLLEYTISSVMIASILLQILNGNKVIVFVPYGLVLIFQLFLLSWNADEIRIQS

GNIAYALYESKWYEQGTDIRKLMFLIVMRCRRVLTLNIGPFGPNTVDVAATRMKVAYSYV

SVMSGNTV*

>SvelOR58

DFIQIEKNSVCQKIIKERLVVCIKQHRAISSFAKLAIKFFYNTVLFHAAVGVSLVGTHCY

IIMFHNSGSPVAVVTNALAGLFVASLYGYFGELFQEQTEKLYDAAKWTNWEHFNVKNRKL

FLIFLINLKRKFHLTGAGIFYLNCSFIY

>SvelOR59

YGSQLISTAVTGAAATFIEVLFAAQCIFIGLQRTILSMRLAKISSNGTESLINCIKHHRS

ILRFHSDVSRVFGTIYFWQTLATTISLCMSIFVFSLAEPGSYNFFLFSLISFGLIVLTLL

FCW

>SvelOR60

LRSSLVQVIFPACYLNLVLMQIFALAWTCNDVKLKSLGIADAVYESPWYDQNKPIQKLLH

NMLHRAQRPLELTIGPFYPMTTDTAVTTIKVAYSYVTVMVNKY

>SvelOR61

MTAGSTMESSTILFLLVYSCECFLYFLSLLIETAVYCIYGQKVINESSLIHEAIYLSNWY

AANVDIHKDLMIIRERVNRPVILTAGKLFPLSVETLAAVLRASYSFLAVMQQT*

>SvelGR1

MEDDSGSDRYLSSKPKSAEEARRKSENIRIITPNTTRGDNEPDDELLEKLHKYDNFYETT

KSLLVLFQIMGVMPIQRGKGKTVYRWFSPVACWAYFIYTIETIFVTIIFKERVKLILQPG

KRFDEYIYGVIFLSILIPHFLLPIGAWTNGNEVAKFKNMWTKFQLKYFKMTGTPIVFKNL

TLISYSLCALSWIVGILVMLAQYYLQPDMLLWHTFGYYHILAMMNSLCSLWFINCTAKGR

VAGWLAESLHAALQTKDSASKLADYREMWVDLSHMMQQLGKAYSGMYALYCLLILLTTIV

ATYGCLTEILDHGLSFKEAGLFLISFYCVTLLFIICNEAHAASRKMGPEFRERLHREKWD

>SvelGR2

HDQFYKDHKLLLILFRWMGVMPVQRIESKVTFSWTSRPMILAYIIYCVTTMLVVLVGYER

IDILLNKSKRFDEYIYAIIFIVYLTPHFFTPFVGWSVAMDVCDYKNSWGSFQLNYYKITG

KDLEFPYLSTLIGVISLGCLFLAVSFLLTLSALMEGFTLYHTTAYLHIITMINMNCALWY

INCRAIGNASTGLADSFKMDLENYCAAYIVKHYRILWLELSELLQKMGNAYARTYSTYCL

LMMANITISIYGFTSEVVDHGLKFTFKEMGLLVDAIYCLTLLFVFCDCSHKASAHIAERV

TWSLMEINLDTVEPDTTQEIQLFLIGIQMNPPKVSLRGYTIVN

>SvelGR3

MDITMHNNSKLMKKNTLILKSQKNCNTFHSCVRLTIKIAQFFSFFPVYGINGPTYKSLQF

RWISWKMVYSLTTFVLFVINLVILFYILSLEGVNILRAAFLAFYMNAFIVKILYIKLARE

WPEFVEQWHKCELSMKNYDPAKNMTKYTNMVASTIIIIAAAEHNILTYNKFASSMKRFSN

VSEVFRDYFERICYWDVFYVTSYSPWIGMICQVLNWQRTFIWTYTDVFIIVIGISFSYKL

QQIKLRIRNMVVLKVSCPYSWRTLREDYARLEELFNVINDKMAWIILVSFSANMFFILLQ

LYHLIKDFYTPLELVYFLISFFLLVGRLMCVCYYGGAPATQTMAIAKALNAVDRSAYNVE

VQRLITYVDSTEMAFTGMNFFKITQNLILKIGTAIMAYELVMTQINEYTKY*

>SvelGR4

SDFVTKMANPMLISANALGLRMINLVYSQKYLRSIFSNIESAEQIMDFKNITNKEYASVK

FIIAVIFLLSVPYQIYTATNEISGENYLLSIILALNFYENFSATCAEFQFSFISNILRNR

FKIINQNLKQMTNPHEDIVYFNSAANLRRKEILLAHIQQLPNNTSRKILKIRDCHHKICV

CCTELNEFYSGQLLFSLTSCGINVLLNLYFAIFGGFQKPDSDGVVKSTATNVMLQIFWGV

YYFIRFANLCVASDRLSREADVTKILICDILRNCRDLNLKDELLIFHLYATSNKINITAS

GFFTVDMGLISSAISVGTTYLVILAQFHGNNN

>SvelGR5

MAHVTPLEAITECHTHLQKIKEKLYEEHDPIVLKCLVRLIYRKKALRYFLGLFICIHIVG

FLYSTQGKFVDAFRTQKVDHAIIRFVDILSNGVLMATNTFALISFSRSACLYKVILDNIK

SKSKTIKRKKRFKYSMLGLYALTCLLLVANVYFFVFKVGWEIYQYLLARDLEFYCFNLSV

YAVLCFARKIQEIFQDVNQILLEVDQELPVCNPIGVTKETILENQPSSKCLEKLRLITQY

HNNICNLLDQFNDYFKTVFVLLLFCMLMNILWDCTIMMEFAVHPRTVNGVNTNTFYSIIC

PLQTLITAGQAAVAALVGENLTNEGKRTSVLCYNFLNKLPSRLVTENDQLIEKQLSFLLD

QSKSRNINLHAGGFFILNFAILGSLAASVATYSIVIIQFMLK*

>SvelGR6

MSGVIVMSKVRTDYEIIQSWLKFSRYLAFTSFDVQGSSKKLKTCLFSKIYPSILFLLYLG

ATVFSFYERVYMYKHFNVSQMILDSLQGFVESLFIEYAVLRSVLGRQRWSRLLKDIEELE

KKISPQINLYVEDSTLKFIRCQILFYHFVYVGVHVYDSVTNWRDVYYSLAFVIFRFTTYY

FMFSTLFMIFLCTWLKNRYIFLECVLNDAVRPKKFKLGFLERDPDFTFEKLKEFSKYYKS

LYLVVQEINQIFGYHFFLLAICTVLEILNAVNYGMPTKNSMVLNDNVIVNILYSTLYAIC

KTRIVMSCDSVEKCGYKIHKTCLVLHESLEKREMKKELIRLACFVKGLGPEFSAAGFWKV

NQEMLSTLFSSVMTYLIIIIQFNMTLK

>SvelGR7

MRSASKTIRKDNSMVVSARFYKNFEYILFLNRIIGLFPITVIQKEDTYILKWSWLRVIIG

SFGVLLFAAVTLTGLSLSYTYPAIYNIRFKAVAAKLVTLAEIITLLFTWLIGQVSVPILF

TEYWQYLSCLKKCDDVIGTVQPSKKITLMIILVFVFTFTCLGMDTYMWARIAIPLMGILN

FIWKMVPFYFTYVFTMIMELNYWIAIYGITIRLKRLNKLICSYINMAQSEKEKDDITLET

LDIIKDKKEIRFIKKNSSSSLPWLSSDTVKGLIVVYYNISHATKAINNFFGVIITTILFT

VIVCLVITPYVLYQQILNSRSNEFIFTQSFWMACHILRLLLIIEPCHKAQNKGAEIRKSI

LKLLNFNLSDEVKKQVKIFWLIAVENNIEFNANSMATINRTILPVIGGAVFTYLAILFQY

NSDVQT

>SvelGR8

MLRFSKNKIHILNKTWNHSLTDNHKEKNIHTSLEFFLCFSQFMGILPQENIFKGKDQMRF

KWLSFRVLYCFFYIVCLSVAMVCCIAKGWEVGYDISLAATHVFYAGSFVNIILYLHLARS

WSSFIQRWCCVDKIMNYRYGYLENVDKKIRWFSFVYLIAGANDHILSVANRYYGLNEILK

ENYTDAVYYNETFPQLFTYIEYSIYGAIYTSVLTIHANFSWAFNDLFIIIMSILLASRFQ

QICKKLSKECQKEKPISYWQQIREDYYRLYILCCDLDSHIAYLIVLSYFLNLFFLLIQLY

HSFESFITFYGKLYFIYSFLYLFFKMGSVSLCAAWINDESKKPAFILSYVPSSSFNVEVK

RLLVQISFNNVTLTGCKMFRITRRLLLSVASAIVTYELVLIQFTILSQDIQNNKV

>SvelGR9

SLGSRYEKLSTSHRGTLTVSTYYNESFPQLFAYVPYNAGTAIFCTIITVHSNLTWAFNDL

FIIIVSVALALRFQQISRKLNREKHKSNSLAFWKEIREDYDRLASFCKELDSHISAIVLL

SYFLNIFFLLIQLYHTLESA

>SvelGR10

MSKKVKKLRDDELTINKCLKKFIMLGQIFGH

FPVQDLSSPTQKFTYKSFKMAYSLFSAIGPAFLCVMQLKKCTTEQFNIFQMQYISRYVTS

CYVAVLFIQLAKNWPKLMEEWRDVERKMQSYGFPKKLNKRIIGVMSFFMTTFFVEYILHQ

TTRIYRVIECNRDPDRSAKFFFGNLTLSHVFQTIPYNLPTSLVIHFWFLQIEFLYAYIDI

FIMVISLCLASRMNQVTKRVRLTSQRQVADEEIWIKLRRDYTRLGTLCERVNDKISNIIL

VSFLPNIFTILIQVYNSFKPKQNYYEQIYYYFSLIVLISRTASVCICGAMISEESRRPLS

ILNTVSHEIYNREIEIFINQISNCEIALNGKHFFNIKRHVILEIAGAIVTYELVLIQFNQ

EYLNSWSKSKRCE*

>SvelGR11

NKNPPNKFWKEVREDYYKVSNLTKVVDAQIAGLVLISFINNIYFLCIQLYNSIKERAAII

DSVYFFYSFGYIVFRVVAVSLYSATLNEAARKPLKYLYSLPTENYTIDVSRLITQINYLP

NGITGHGFFLITKNFLLQAAATVVTFELMIFQFSPAIKTLASSRNEFTCIR

>SvelGR12

LCIHLATKWPKFVKEWHLIEASMKNYETTNNLKRKVIIICMSILSMAVIEHGMVIFDRMK

KSIRANPNSFYDVLHDYFAIYRFPEVFHILPYSIWLGLLFQFAVFQKTFMWTFLDVFIIL

VSTCFNFRLEQITKKLTYLSKMQVADENVWRVIREDYVKLSRLCDLINKHLCWFIIISYL

TNIYHILSQLFSSLRPIENSFEKIYFYLSFFLLILRVTLVCVFGGAVYDQHGETVKILTG

VPSSVYNIEVERFITHLATCEMVLSGKHFFRITRNLILKVTSAIVTYELVLIQFNAQDFK

KKDL

>SvelGR13

KVKWLHIETHKVIYTFNELFGQLLLLMAVQCSLQVLDFGVFLIEQVLPKLTFEVDTLFIY

VAFIILNLIWFSATQFRCDSARHQSVRLLSVCYNLLDNQCSSSELHQELKTLTNQIKNRP

VHFTAAEFFEIKKSTTFS

>SvelGR14

MLSEATYKIIQPLVYTSRIFAICPVAFTKIGSFYQIKYSSFLVLYSYAFVIAFGVATVLG

VKTDIGEGTNAIRMKVKKTRYISICDMFIVFVIALYGVISIPYKMRQFVKMLDIWSHVDS

VIPITKYQKYRRISIVFLSTTLLTTTALFLFDICIYCFTSKASHSPIFYVEMYFGYYVLY

YIMFLHEIFFWHMVFFIHIRLVSLNENLSNEKLNIKNGLKRLFAISYNTRNGNKDSSGEK

RPKMSGSEVTKFGKNTAERMKELLKIYGKIRESVEILNESASYGIVLIFLSCLLHLVVTP

YFLLLEIFIKSNGSIIFILLQGVWFLAHVGRLLIMVEPCQKCINEYKITQNLVSEMVLLD

VDAETKKMLKCFAANFSYADISFHACGFFRIDRNLLTSVTGAVTTYLVILFQFNGN

>SvelGR15

MDTYMWAKIAIPIIGPFDFIWKMTLFYFSYVFTMIIELNYWMTINGVTRRLNMLNKLMVS

YINIASSQTQENYFSGLRELNFKKKEPISFRLKRKSCVDCTLLSSGTVKNFIAVYENLSD

ATKAANEFYGLVISAVLFAVFASLVLTPYVLFQQISNVKSKSFLFTQSYWMICHILRLLF

VIEPCNTAQRQGIQIKKSVVKLLQLQLSDETYKQVKILWLTVIENVIEFTASDMIVINRK

LLPPICGAVFTYLTILFQYNASA

>SvelIR8a

MHFITIFLAFHFVSELKAQTFKIVTVLQDDQERQVQAFEDIITTINKNSNVTFSRIKLDD

VKDEFQQICDGLANGISLLMDFTWSGNEVAENIAVNMSIPYIHAEVSVAPFLILLDAYLD

SRNSTDVLLIFDKEDYIDQALYFWIDSTRLRMVMSETLDRATAEKIRKIRPIPNTFAIVA

ANRNMNKLFLQAQNGDLLTLPDRWNLVFTDFQSKFFPRSILNNSSVSLLYFKEDLCLDLL

DKDSCPTNFDLQEQFLYWLAYSINRVVKNMVDEKLDFPNENFECNKTKFADETKERFEEI

LDFVISENSKVLTRVDNTVRVRIRGQIEKLYNGSMQLIAQYRYGRLEVEPGKKIDPIKPF

YRIGITHALPWSYKETDPDTGEQYWTGYCADFAQKLSEIMNFDYVFVEPKTGTFGEKVNG

TWNGVVGDLARGDTDMAITAIIMTADREEVIDFVAPYYEQTGITIVMRKPVRKTSLFKFM

TVLKLEVWLSIVAALIVTGFMVWFLDKYSPYSARNNKKAYPYPCREFTLKESFWFALTSF

TPQGGGEAPKALSGRTLVAAYWLFVVLMLATFTANLAAFLTVERMQAPVQSLEQLARQSR

INYTVVQDSETHKYFINMKNAEDTLYRMWKELTLNASTDDTRYRVWDYPIREQYGHILLA

INDSNPVANASEGFRIVDEHLDADFAFIHDSSEIKYEISRNCNFTEVGEVFAEKPYAVAV

QQGSHLQDDLSKVILDLQKDRFFEELQAKYWNSSAKGDCPSTDDNEGITLESLGGVFIAT

LFGLALAMITLAGEVLYYRRKGQKEKEKTKQQLSKNLSPNIQTLKPPPKYTSEPPTLEKF

NIGKTITIGTTFKPVNLKEQLKEADNVNIAHISLYPKARNRIPRVE*

>SvelIR21a

DSSVIIKMIAKVFFSVSLFLKLVGCLSFDGFETSVTKRALQKSHEKPRLTKLSELFLEKD

LYDEDADASLIGLLNVIANRYLSDCTTAILYDNYTESSDNVFLKKFFMQYRLTYVHGRIP

NDYHIQLGELVNKNDKKCVHFILFLRDVMRCQDVVDKRSEKVVVVAKSSQWRVQEFLAGE

MSQEIANLLVIVKSDKLLPQKQEAPYILYTHKLFVDALGSSQPVVLTSWAAGSFSRNVSL

FQTKIEHGFSGHRFIISVAHQPPYVIKKQRNENDEFEYEGIEVKLVTLLSKMFNFSIDFK

EASEVKVLGSGEAVIKAVKSGNLNLGIGGIYITKDRFDSGIFHWHDEDCASFISLASTAL

PRYRAIMGPFRWSVWVCLIMVYLSAICLFSFSDKLTLRDLIKNPEEIENMFWYVFGTFTN

CFTFTGRRSWTKAEKNTTKLLVGVYWIFTIIITACYTGSIIAFVTLPIFPAVVDTIEQLL

GGRYQIGMQDKGGWPKWFKNMTDKSSLTLLKKVDYVPDIESGLKNVTRAFFWPYALLASR

EELKFIAKTNFSIESKRQLLHISQQCFVPYKVGIVLPQHSVYSAVLADGIQRMVQAGLTL

KMKGDVEWAMLRSATGKLLAASSRVGSIKTLTYEDRALTLDDTQGMFLLLGAGFLAGGAI

LLSEIFGGCINLCKIKNEDSRASTSSSLPSNPRLHEMQTGRERYHSLSLAQHQSRSMSLA

SNEETLKPKIEEPLFGSCSHGDNEISPFDKNILDEDDLSDLEVDDNIETTSKNGCRENDE

HTEHDKEMNQVC*

>SvelIR60a

VIFGFGLLFYISKCNTKKEVVTFRTLEKCVAAAFEIFMGVAVPIQPITHFTRVFFIGLAF

VAIIINSIYTSSLIYYLQNPIREHQISSIPEVAISGMGVGGPPKYIQIFKSSKSPLAEIL

LRRYQTVNDTLDTYTYWLNLVANKRNISTMTVKLCVLYLMSKKKSVVVDRRGDSKVFIIP

RTIITQSIGIMMRRGAFAREKINTVVQRLILEAGLKEKIKSYYIREGKSEEGSVMQESID

IIDLDDDDEGISPLSPYHLEGAFAILALGHGFALLTFIIEIICYRINKQRKKRV*

>SvelIR75a.1

VLLLQSNTNRIRHLQDLYGAKLDFGVEDTPFNRYYFTGPSDRANEHWRKIIYENKIKSPG

DDKFLNAGDGMTLVRDSYFAFHVEQNVANYLIETTFSNSQKCSLRVVETIYKSDIPHLSC

PKHSPYIEFFLVSFRRLAETGVHNREFRKCFTKQPVCEGRGNNFVSVGLIECYFAFLVFG

TGVCLSLIIFALENLSQTYLHFQCAL*

>SvelIR75a.2

MLTINFVFFLLSFVNAALTKSYNDKVSLVIDFIKIHKSPEIILDNLCLSKGNKIYFQNQV

MQNNMRYLGYNNNSLVFEEFEYNYTFTVVTELECNFDEFLNNALGTPLVAYPNVWIIFGD

FLKIKKLNLYFPVNSLFVIPTRDTEYNKTLLKTVYKTNKNFTGYVESTLGEWNEFDHVCN

FKEIILTRNRTNFFGMHLRVSYVITDKSTFQHLTDYREKHLDNLTKINYILFAVISDLLN

TTQVKIFRNDWGFKGKHPKKPFTKGMFGDLTYDRADIGGTVSFTPATRLKYFKYLFAPMK

EFSVNFVFRAPSLAYYSNIFALPFDKLVWLTIGGLLVFCSIIIKLIFDWETKERVFVKEM

EDNKENAPTILDVIMMQIAIMCQMDFFYEPKGLSGKIATFMVLITFTFIYTSFSARIVLL

LQSNT

>SvelIR75b

MKIFNFLLFSFTLNFKNVSSSKKILFVRDYLKLHNETSILQANLCWKQDDLIQLANVLSK

TRVRYALINSRELLPVENVHNMMFITDMDCNFFDLLLSEVLQKYLYKFPNKWIIFGSLEK

LKIMNYYFPINSYIMVLDGTYSNNTFNIKTLYKLNSTVLEYSTSTLGIWSIDKGFWFFND

LVLVRNRSDFMKMPLKVSYIILDKNSLKHLMDNRERHLDKLTKINYLMYWHFIDIWNMSQ

KIIIRDDWGFEPHPTKLYHKGMLGDLYYGRADISGTLSFTPTERLKYFKYLVATAKELGC

TFIFKAPPLPYSTNMFALPFDKYVWISCGIVVILCCLVIWIILKWEETQKIFVDTRKAAD

ENAPNFLDTLMMQIAVVCQMDYYYEPKSSSGKIATFTLLLTFSYLYTAFSARIVLLLQSS

ANNLKDVKGLYDANINMGVENIPYNRYYFTRPAKRTNEYLRKLIYENKIAPKGSPEPKFY

QALEGIEMVRTSYFAFHMEITTAINLVESLFTNEEKCSVRTVETIFKSDIAH

>SvelSNMP1a

MQFTKNKLKQIFFVLFPNLRTAEFAVVSTKKVYGFVKKVKSSKRKMRFPVKLAIGSAFAF

FFIIIVGFIMFPKMIKGKIKGMINLSPGSEIRGMFVNVPFGLDFNIFIFSVLNPEEVQNG

GIPKLQEYGPFCYEEWKTKINVEDNDADDTMSYNPVDTFLPARGKYKECVDPDTEVTIAH

PMILGMINAVARQKPGALTLAGKAIKSIWSNPTSIFVTVKARELLFDGVVIHCGVQDFAG

KAICTNLRSEPSLVKLNENDLAFSLLGSKNGTPGKTFKAYRGNTDFSKVGSIVTYDGQPK

QEIWNSSKCDVIVGTDGTIFPPLLKKEDGLASFAPDLCRSLIAFWGNNEKYDGITVSAYY

ATLGDPSNNPDDKCFCSTPDTCLKQGMMDLYKCAKVPIYVSQPHFYDCHESYLKGVKGLK

PDVEKHRIKILFEATTGSPVWAKKRLQFNMPLEPNQKIDLFHNFTPTVLPMFWVEEGIAL

NDTFTKPLKDFFTIKKIVGVIKYVVLVSSIVGFAIAAYLYFKEDDSISTKSVQKVEPAAE

RKSGISTVYNGDEFGGKVNEGYEDKY*

>SvelSNMP1b

MRVAEKLIYASLVIIFMTVLTKLWLFDTILKIGIRDQTALRKRNEVRDVYLKIPFPLDFK

VYLFNVTNPMEIQKGAKPIVKEVGPYWYDEFKEKIDVVDNEMEDSLTYTPYDIFRFNQNK

SGSLSDEDYVTILNPLIVGMVNQVMLDSPVFISIVSQAISKIFNNPESIFLKTKVKDILF

DGAELNCKGKDFATSTVCSQLKGKIPGLKFKEGDEKIFLFSLLGSRNATMTSRTKVLRGI

KKSKDLGRLIEFDGKKQLDLWSTTYCNRFNGTDGWIFPPLLEPEEGLRSFSTDLCRNVKV

EYVNDTTLKKLKVRIYEGNLGDQTNNEEDKCYCKESGSCLKKGIFDLTKCMHVPILATLP

HFLMVDESYLNYIDGLEPDFDKHSLRVFFEPMTGSPIKAAKRMQFNFEIQQYSKVPLFTT

LPNALFPLLWIEESVDLEGDLLKKLQGVFLLLTIADIMIYVLLCFGIVTLSIAIFYHYKN

KKEVSITPVYEIKKIENEDDEGNDMSTLTRPEKKSEKTGHTNPVMSGHEFDKY*

>SvelSNMP2

MFINCCSSRMVFIWTVLVALCLGGSLLLAFWGMPKIIKNQLHKQTQLENNTDQWDRFMEL

PFSLDFTVRFFNVTNPDDVLYKNATPVLKETEPYTYKMTLKKRNVRVDDDQEDSVTYDRQ

MLFEFDDSGTTKEDDEVTIINAVLMSALQLTGDIERIAMAGCLEKLLKPHGMDTIFIKKP

VRQILFDGIPFSFENSTGYACKIVREKMLKVIGGIRVIEQMDGEAEGNGYLKFSVFNYKR

DSYTDKKPKPDGVYTVNRGIKDMSLIGNIMRWNDNTEVSIWGNSASTNNDTCKRVSGTDS

TIYKPQLKENEELMIFNTDLCRTVRLVYKNSEESYKGIKAFRYEPSPLIFRPSTAFAEND

CFCAKATKDVFGKDNCYLDGAFDFKPCLGAPVLVSQPHFLNADKHYLEGVEGLSPDKSKH

DIFLLIEPNTGTPLQGMKRIQLNNVLRREPLISFITPKNMTETVVPILWLEEGVNLPQKY

VDLVNNQYFKAVRIADGVKYALIALFASLFAVCLFFVWRKEFLKKKTSV*
