# Supplementary material for: Candidate Chemosensory Genes Identified in the Adult Antennae of Sympiezomias velatus and Binding Property of Odorant-Binding Protein 15
Source: Front Physiol. 2022 May 31;13:907667. doi: 10.3389/fphys.2022.907667 (PMC9193972; doi:10.3389/fphys.2022.907667)
Supplement: Supplementary file 4 [file Table2.DOCX]

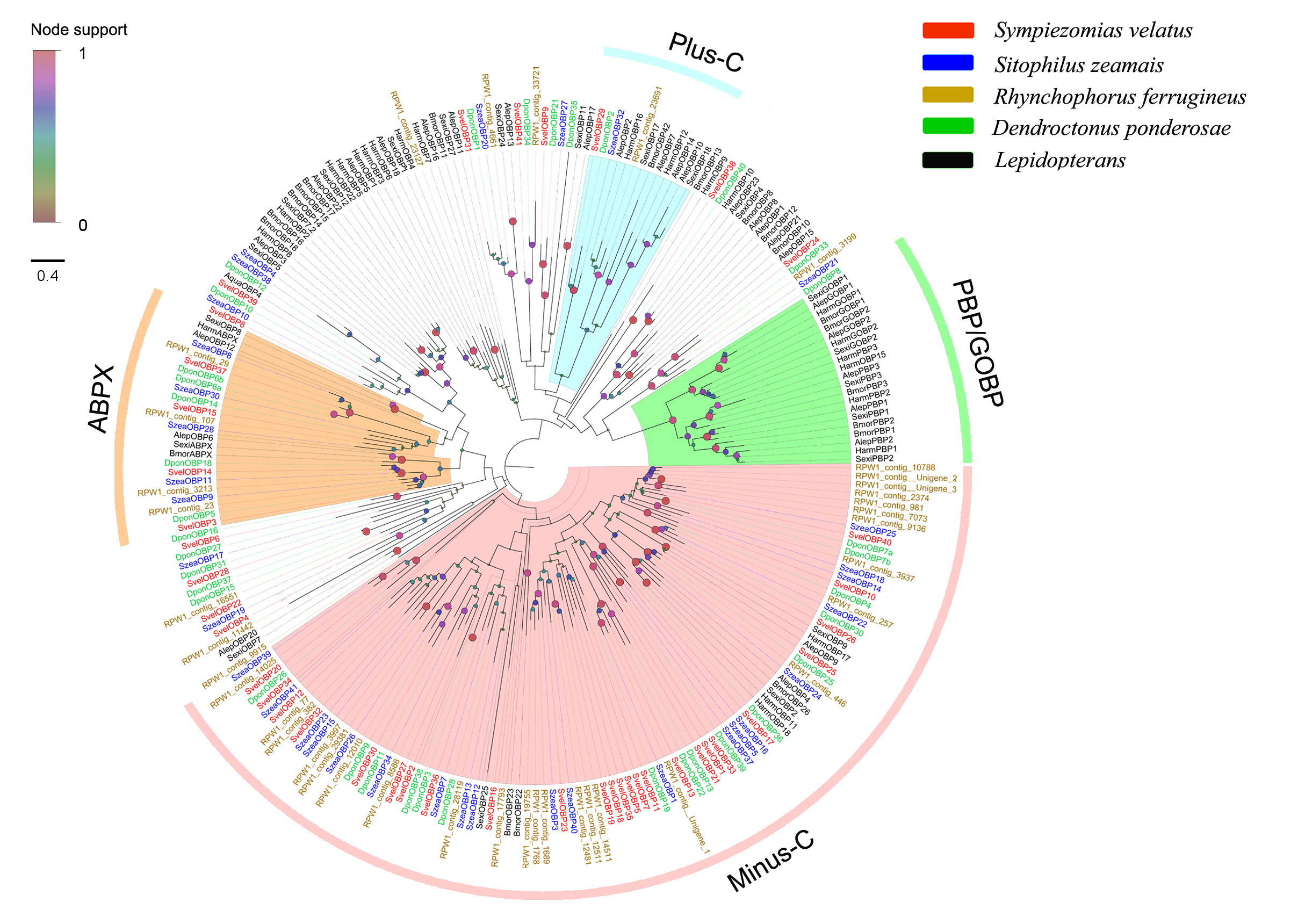


**Figure S2.** The neighbor-joining tree of odorant binding proteins (OBPs). The amino acid sequences used for phylogenetic analysis were from four weevil species including *Sympiezomias velatus* (Svel, red), *Sitophilus zeamais* (Szea, blue), *Rhynchophorus ferrugineus* (RPW1_X, yellow) and *Dendroctonus ponderosae* (Dpon, green), and four lepidopteran species including *Bombyx mori* (Bmor), *Helicoverpa armigera* (Harm), *Athetis lepigone* (Alep) and *Spodoptera exigua* (Sexi). Tree was constructed using MEGA 7.0 with the Jones-Taylor-Thornton (JTT) substitution model, pairwise deletion of gaps and 1000 bootstrap replicates. The bootstrap values are indicated by the colored circles and increases with the brightness and size of the circles.
