# Supplementary material for: Candidate Chemosensory Genes Identified in the Adult Antennae of Sympiezomias velatus and Binding Property of Odorant-Binding Protein 15
Source: Front Physiol. 2022 May 31;13:907667. doi: 10.3389/fphys.2022.907667 (PMC9193972; doi:10.3389/fphys.2022.907667)
Supplement: Supplementary file 5 [file Table5.docx]

**Table S2.** Binding data of the tested compounds to the recombinant SvelOBP15

| **Compouds** | **CAS No^#^** | **Formula** | **Source** | **IC_50_ (µmol/L)** | **K_i_**  **(µmol/L)** |
| --- | --- | --- | --- | --- | --- |
| **Alkanes** | | | | | |
| Pentadecane | 629-62-9 | C_15_H_32_ | elm | - | - |
| Tetradecane | 629-59-4 | C_14_H_30_ | elm | - | - |
| Dodecane | 112-40-3 | C_12_H_26_ | elm | - | - |
| n-Hexadecane | 544-76-3 | C_16_H_34_ | elm | - | - |
| **Alcohols** | | | | | |
| Z-3-hexen-1-ol | 928-96-1 | C_6_H_12_O | peanut, elm^#^, maize, cotton^#^ | - | - |
| 4-Methyl-5-nonanol | 154170-44-2 | C_10_H_22_O | pheromone^*^ | - | - |
| 3-Methyl-4-octanol | 26533-35-7 | C_9_H_20_O | pheromone^*^ | - | - |
| (E)-3,7-Dimethyl-2,6-octadien-1-ol | 106-24-1 | C_10_H_18_O | pheromone^*^ | - | - |
| 1-Octen-3-ol | 3391-86-4 | C_8_H_16_O | peanut^#^ | - | - |
| **Aldehydes** | | | | | |
| trans-2-Hexenyl aldehyde | 6728-26-3 | C_6_H_10_O | peanut | - | - |
| Nonanal | 124-19-6 | C_9_H_18_O | elm | - | - |
| **Ketones** | | | | | |
| (1,1`-Bicyclopentyl)-2-one | 4884-24-6 | C_10_H_16_O | elm | - | - |
| 4-Methyl-5-nonanone | 35900-26-6 | C_10_H_20_O | pheromone^*^ | - | - |
| **Acids** | | | | | |
| Palmitic acid | 57-10-3 | C_16_H_32_O_2_ | peanut | - | - |
| Geranic acid | 459-80-3 | C_10_H_16_O_2_ | pheromone^*^ | - | - |
| **Esters** | | | | | |
| cis-3-Hexenyl acetate | 3681-71-8 | C_8_H_14_O_2_ | peanut^#^, elm^#^, maize | - | - |
| trans-2-Hexenyl acetate | 2497-18-9 | C_8_H_14_O_2_ | cotton | - | - |
| Diisobutyl adipate | 141-04-8 | C_14_H_26_O_4_ | elm | - | - |
| **Benzene derivative** | | | | | |
| 3',4'-Dimethylacetophenone | 3637-01-2 | C_10_H_12_O | mungbean | - | - |
| 4-Ethylacetophenone | 937-30-4 | C_10_H_12_O | elm | - | - |
| 1-Methylnaphthalene | 90-12-0 | C_11_H_10_ | elm | - | - |
| Diisobutyl phthalate | 84-69-5 | C_16_H_22_O_4_ | elm | 15.82±0.42 | 12.57 ± 0.34 |
| Methyl salicylate | 119-36-8 | C_8_H_8_O_3_ | elm^#^, maize | - | - |
| Benzalehyde | 100-52-7 | C_7_H_6_O | elm | - | - |
| **Terpenes** | | | | | |
| α-Farnesene | 502-61-4 | C_15_H_24_ | peanut^#^, elm^#^, cotton^#^ | - | - |
| β-Ocimene | 13877-91-3 | C_10_H_16_ | peanut, elm, cotton*^#^* | - | - |
| Limonene | 138-86-3 | C_10_H_16_ | peanut, elm, cotton^#^ | 14.68±0.53 | 11.66±0.47 |
| (+)-α-Pinene | 7785-70-8 | C_10_H_16_ | peanut, elm, maize, cotton | - | - |
| Nerolidol | 7212-44-4 | C_15_H_26_O | peanut, cotton^#^ | 16.92±0.46 | 12.94±0.43 |
| (+)-β-Pinene | 19902-08-0 | C_10_H_16_ | peanut, maize, cotton*^#^* | - | - |
| (-)-trans-Caryophyllene | 87-44-5 | C_15_H_24_ | peanut, elm^#^, maize, cotton | - | - |
| (E,E)-Farnesol | 106-28-5 | C_15_H_26_O | maize^#^ | 9.26±0.43 | 7.36±0.36 |
| Linalool | 78-70-6 | C_10_H_18_O | peanut, elm^#^, maize | - | - |

^#^ represents herbivore-induced plant volatiles (HIPVs) that do not exist in healthy plants. ^*^ represents reported aggregation pheromone components of other curculionids. “-” indicates that the IC50 value exceeded 20 μmol/L, and thus, the binding affinity (Ki) of the volatile is not calculated here. Low Ki values mean high binding affinity between protein and ligands.
