# Supplementary material for: Candidate Chemosensory Genes Identified in the Adult Antennae of Sympiezomias velatus and Binding Property of Odorant-Binding Protein 15
Source: Front Physiol. 2022 May 31;13:907667. doi: 10.3389/fphys.2022.907667 (PMC9193972; doi:10.3389/fphys.2022.907667)
Supplement: Supplementary file 6 [file Table3.DOCX]

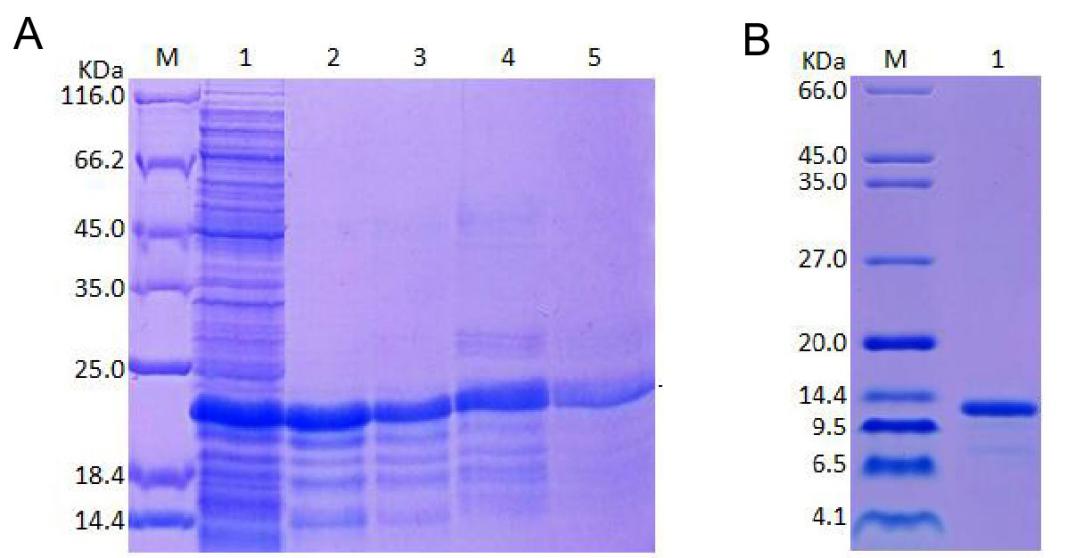


**Figure S3.** Prokaryotic expression and purification of recombinant SvelOBP15. (A) SDS-PAGE analysis of purification of recombinant SvelOBP15; M: protein molecular weight Marker; lane 1: pellet of recombinant protein after sonication; lanes 2-3: protein after elution with 20 and 50 mM imidazole solutions; lanes 4-5: protein after elution with 500 mM imidazole solutions; (B) tricine-SDS-PAGE analysis of the target protein with His-tag removed by recombinant enterokinase. M: protein molecular weight Marker; lane 1: re-purification of the target protein after the removal of His-tags.
