# Supplementary material for: Candidate Chemosensory Genes Identified in the Adult Antennae of Sympiezomias velatus and Binding Property of Odorant-Binding Protein 15
Source: Front Physiol. 2022 May 31;13:907667. doi: 10.3389/fphys.2022.907667 (PMC9193972; doi:10.3389/fphys.2022.907667)
Supplement: Supplementary file 7 [file Table4.DOCX]

**Table S1.** Primers for qRT-PCR analysis of chemosensory genes in *S. velatus*

| Primer | Sequence（5’-3’） |
| --- | --- |
| SvelOBP1-F | GCGCTCTTCTGTTTGCAAGT |
| SvelOBP1-R | ACCGCTCGAGCTATGGTTTC |
| SvelOBP2-F | AGAAGATGTGCCGTCGATGA |
| SvelOBP2-R | TCCCCAAAAATGCTCGTTCG |
| SvelOBP8-F | CAAAGCCACTCCAGATGAGGT |
| SvelOBP8-R | AATATTCCCAGGCAGCGACA |
| SvelCSP1-F | TACGCCAGATGGCCTTGAAC |
| SvelCSP1-R | GGCTGCTCTACAGGTTCCAC |
| SvelCSP3-F | CCAGATGCTTTGGACAACGG |
| SvelCSP3-R | TGGCTGCTACTTCATTCCACA |
| SvelCSP5-F | AGGTGTCGCAATTTTCGTGC |
| SvelCSP5-R | TCCACCATGGGCGTTTGTTA |
| SvelCSP9-F | ACCTTCCAGATGCTCTTCACA |
| SvelCSP9-R | GTTGTCGATCAAATGGCGCA |
| SvelOR18-F | GCGCCCAAATTACAATGGCT |
| SvelOR18-R | CTAGCTGTTCCACAGTGCCA |
| SvelOR45-F | TGTGGCCAGTAGATGTGGAC |
| SvelOR45-R | AAGCCAGAAGCCGTGCTAAT |
| SvelIR8a-F | TGCAGGCTCAAAACGGAGAT |
| SvelIR8a-R | GGGGCATGAGTCTTTGTCCA |
| SvelIR21a-F | GGAGGCTGGCCTAAATGGTT |
| SvelIR21a-R | GGAAGCCAGCAATGCGTATG |
