# Supplementary material for: Candidate Chemosensory Genes Identified in the Adult Antennae of Sympiezomias velatus and Binding Property of Odorant-Binding Protein 15
Source: Front Physiol. 2022 May 31;13:907667. doi: 10.3389/fphys.2022.907667 (PMC9193972; doi:10.3389/fphys.2022.907667)
Supplement: Supplementary file 9 [file Table6.DOCX]

**Table S3**. The Blastp match of *S. velatus* candidate OBPs and CSPs.

| **Gene name** | **ORF**  **(aa)** | **Complete**  **ORF** | **Pfam ID** | **SP^a^**  **(aa)** | **C nb^b^** | **Best Blastp Match** | | | |
| --- | --- | --- | --- | --- | --- | --- | --- | --- | --- |
|  |  |  |  |  |  | **Acc. number** | **Species** | **E value** | **Identity(%)** |
| SvelOBP1 | 134 | Y | PF01395 | 1-19 | 4 | [ANE37547.1](https://www.ncbi.nlm.nih.gov/protein/ANE37547.1?report=genbank&log$=prottop&blast_rank=1&RID=NEFXPPFT016) | *Rhynchophorus ferrugineus* | 2e-13 | 32.1 |
| SvelOBP2 | 160 | Y | PF01395 | 1-16 | 4 | [QCT83288.1](https://www.ncbi.nlm.nih.gov/protein/QCT83288.1?report=genbank&log$=prottop&blast_rank=1&RID=NEJ163UF014) | *Sitophilus zeamais* | 1e-29 | 39.6 |
| SvelOBP3 | 133 | Y | PF01395 | 1-20 | 6 | [AIY61044.1](https://www.ncbi.nlm.nih.gov/protein/AIY61044.1?report=genbank&log$=prottop&blast_rank=1&RID=NEJ2UDAW016) | *Dendroctonus armandi* | 3e-58 | 67.4 |
| SvelOBP4 | 147 | Y | PF01395 | 1-24 | 6 | QCT83273.1 | *Sitophilus zeamais* | 2e-24 | 44.5 |
| SvelOBP5 | 159 | Y | PF01395 | - | 4 | [AVR54527.1](https://www.ncbi.nlm.nih.gov/protein/AVR54527.1?report=genbank&log$=prottop&blast_rank=1&RID=NEJD1UP4016) | *Sitophilus zeamais* | 2e-23 | 37.9 |
| SvelOBP6 | 118 | Y | PF01395 | 1-18 | 6 | [AGI05186.1](https://www.ncbi.nlm.nih.gov/protein/AGI05186.1?report=genbank&log$=prottop&blast_rank=1&RID=NEJJHEVK014) | *Dendroctonus ponderosae* | 3e-31 | 48.2 |
| SvelOBP7 | 130 | Y | PF01395 | - | 4 | AVR54527.1 | *Rhynchophorus ferrugineus* | 6e-13 | 29.3 |
| SvelOBP8 | 148 | Y | PF01395 | - | 8 | ANE37553.1 | *Rhynchophorus ferrugineus* | 8e-71 | 71.6 |
| SvelOBP9 | 234 | Y | PF01395 | 1-18 | 8 | [AMP19495.1](https://www.ncbi.nlm.nih.gov/protein/AMP19495.1?report=genbank&log$=prottop&blast_rank=1&RID=NEK0VBPP014) | *Tomicus yunnanensis* | 9e-76 | 53.9 |
| SvelOBP10 | 493 | N | PF01395 | - | 19 | QKV34985.1 | *Dendroctonus adjunctus* | 1e-132 | 48.8 |
| SvelOBP11 | 125 | N | PF01395 | 1-18 | 4 | [ANE37546.1](https://www.ncbi.nlm.nih.gov/protein/ANE37546.1?report=genbank&log$=prottop&blast_rank=1&RID=Y8JH29UU016) | *Rhynchophorus ferrugineus* | 4e-24 | 36.0 |
| SvelOBP12 | 143 | N | PF01395 | - | 4 | [ATU47278.1](https://www.ncbi.nlm.nih.gov/protein/ATU47278.1?report=genbank&log$=prottop&blast_rank=1&RID=NEKFEWRN014) | *Rhynchophorus ferrugineus* | 2e-34 | 55.9 |
| SvelOBP13 | 135 | Y | PF01395 | 1-24 | 4 | QCT83255.1 | [*Sitophilus zeamais*](https://blast.ncbi.nlm.nih.gov/Blast.cgi#alnHdr_QCT83255) | 1e-43 | 59.1 |
| SvelOBP14 | 139 | Y | PF01395 | 1-23 | 7 | [QCT83265.1](https://www.ncbi.nlm.nih.gov/protein/QCT83265.1?report=genbank&log$=prottop&blast_rank=1&RID=NGBTRABA014) | *Sitophilus zeamais* | 1e-66 | 72.7 |
| SvelOBP15 | 150 | Y | PF01395 | 1-26 | 6 | AHE13800.1 | *Lissorhoptrus oryzophilus* | 4e-56 | 57.0 |
| SvelOBP16 | 138 | N | PF01395 | - | 3 | [APG79365.1](https://www.ncbi.nlm.nih.gov/protein/APG79365.1?report=genbank&log$=prottop&blast_rank=3&RID=X0GGU7EM01R) | *Cyrtotrachelus buqueti* | 3e-16 | 37.4 |
| SvelOBP17 | 140 | Y | PF01395 | 1-17 | 4 | [AVI04900.1](https://www.ncbi.nlm.nih.gov/protein/AVI04900.1?report=genbank&log$=prottop&blast_rank=1&RID=Y8JYZPCK013) | *Anthonomus grandis* | 2e-38 | 45.3 |
| SvelOBP18 | 134 | Y | PF01395 | 1-21 | 4 | XP_030764727.1 | *Sitophilus oryzae* | 3e-14 | 37.4 |
| SvelOBP19 | 126 | Y | PF01395 | 1-18 | 4 | [AVR54527.1](https://www.ncbi.nlm.nih.gov/protein/AVR54527.1?report=genbank&log$=prottop&blast_rank=1&RID=NGCTBEZH014) | *Rhynchophorus ferrugineus* | 6e-23 | 37.6 |
| SvelOBP20 | 154 | Y | PF01395 | 1-20 | 5 | [QCT83293.1](https://www.ncbi.nlm.nih.gov/protein/QCT83293.1?report=genbank&log$=prottop&blast_rank=2&RID=NGCX5A2J014) | *Sitophilus zeamais* | 2e-45 | 51.4 |
| SvelOBP21 | 134 | Y | PF01395 | 1-19 | 4 | XP_030764727.1 | *Sitophilus oryzae* | 8e-22 | 34.4 |
| SvelOBP22 | 141 | Y | PF01395 | 1-23 | 6 | [QFO46770.1](https://www.ncbi.nlm.nih.gov/protein/QFO46770.1?report=genbank&log$=prottop&blast_rank=2&RID=NY5V76HW014) | *Cylas formicarius* | 5e-14 | 35.3 |
| SvelOBP23 | 130 | Y | PF01395 | 1-18 | 5 | [XP_030764727.1](https://www.ncbi.nlm.nih.gov/protein/XP_030764727.1?report=genbank&log$=prottop&blast_rank=1&RID=Y8KPAZ1V016) | *Sitophilus oryzae* | 3e-38 | 46.3 |
| SvelOBP24 | 147 | Y | PF01395 | 1-16 | 6 | QCT83275.1 | *Sitophilus zeamais* | 1e-42 | 48.2 |
| SvelOBP25 | 150 | Y | PF01395 | 1-19 | 6 | [XP_019758050.1](https://www.ncbi.nlm.nih.gov/protein/XP_019758050.1?report=genbank&log$=prottop&blast_rank=1&RID=Y8KR2EU3013) | *Dendroctonus ponderosae* | 1e-78 | 78.0 |
| SvelOBP26 | 134 | Y | PF01395 | 1-19 | 4 | [APG79378.1](https://www.ncbi.nlm.nih.gov/protein/APG79378.1?report=genbank&log$=prottop&blast_rank=2&RID=NY61CE78016) | *Cyrtotrachelus buqueti* | 6e-53 | 61.9 |
| SvelOBP27 | 110 | N | PF01395 | - | 2 | [QCT83288.1](https://www.ncbi.nlm.nih.gov/protein/QCT83288.1?report=genbank&log$=prottop&blast_rank=2&RID=NY6GW50D016) | *Sitophilus zeamais* | 2e-19 | 50.0 |
| SvelOBP28 | 128 | Y | PF01395 | 1-23 | 6 | [ALM64973.1](https://www.ncbi.nlm.nih.gov/protein/ALM64973.1?report=genbank&log$=prottop&blast_rank=2&RID=NY6M6T93016) | *Dendroctonus armandi* | 6e-16 | 33.9 |
| SvelOBP29 | 256 | Y | PF01395 | 1-20 | 12 | [QCT83286.1](https://www.ncbi.nlm.nih.gov/protein/QCT83286.1?report=genbank&log$=prottop&blast_rank=2&RID=NY6S97V4014) | *Sitophilus zeamais* | 3e-102 | 60.7 |
| SvelOBP30 | 115 | N | PF01395 | - | 2 | [AKK25144.1](https://www.ncbi.nlm.nih.gov/protein/AKK25144.1?report=genbank&log$=prottop&blast_rank=2&RID=NY6WX6DF016) | *Dendroctonus ponderosae* | 2e-32 | 51.5 |
| SvelOBP31 | 177 | Y | PF01395 | 1-19 | 8 | [XP019773433.1](https://www.ncbi.nlm.nih.gov/protein/1130205352?report=genbank&log$=protalign&blast_rank=1&RID=YH5PJ6CC015) | *Dendroctonus ponderosae* | 2e-94 | 79.2 |
| SvelOBP32 | 134 | Y | PF01395 | 1-17 | 4 | [QFO46772.1](https://www.ncbi.nlm.nih.gov/protein/QFO46772.1?report=genbank&log$=prottop&blast_rank=2&RID=XE75SXCK01R) | *Cylas formicarius* | 5e-37 | 43.9 |
| SvelOBP33 | 134 | Y | PF01395 | 1-15 | 4 | [QCT83291.1](https://www.ncbi.nlm.nih.gov/protein/QCT83291.1?report=genbank&log$=prottop&blast_rank=1&RID=XE77XV3S016) | *Sitophilus zeamais* | 5e-33 | 41.8 |
| SvelOBP34 | 132 | Y | PF01395 | 1-19 | 4 | [AHA39270.1](https://www.ncbi.nlm.nih.gov/protein/AHA39270.1?report=genbank&log$=prottop&blast_rank=2&RID=NY72W6PS016) | *Monochamus alternatus* | 2e-08 | 30.5 |
| SvelOBP35 | 133 | Y | PF01395 | 1-18 | 4 | [QCT83257.1](https://www.ncbi.nlm.nih.gov/protein/QCT83257.1?report=genbank&log$=prottop&blast_rank=2&RID=Y8MP2CZ2016) | *Sitophilus zeamais* | 6e-18 | 34.8 |
| SvelOBP36 | 76 | N | PF01395 | - | 2 | [QCT83266.1](https://www.ncbi.nlm.nih.gov/protein/QCT83266.1?report=genbank&log$=prottop&blast_rank=1&RID=NH24VSF5016) | *Sitophilus zeamais* | 7e-16 | 49.3 |
| SvelOBP37 | 97 | N | PF01395 | - | 3 | [AMP19491.1](https://www.ncbi.nlm.nih.gov/protein/AMP19491.1?report=genbank&log$=prottop&blast_rank=1&RID=NH2KFZM4016) | *Tomicus yunnanensis* | 1e-49 | 75.0 |
| SvelOBP38 | 180 | N | PF01395 | - | 7 | [QKV34990.1](https://www.ncbi.nlm.nih.gov/protein/QKV34990.1?report=genbank&log$=prottop&blast_rank=1&RID=XE80XXR2013) | *Dendroctonus adjunctus* | 1e-49 | 74.2 |
| SvelOBP39 | 66 | N | PF01395 | - | 1 | [ANE37553.1](https://www.ncbi.nlm.nih.gov/protein/ANE37553.1?report=genbank&log$=prottop&blast_rank=2&RID=NH2ZX9XB016) | *Rhynchophorus ferrugineus* | 1e-17 | 53.0 |
| SvelOBP40 | 136 | Y | PF01395 | 1-19 | 4 | [QCT83279.1](https://www.ncbi.nlm.nih.gov/protein/QCT83279.1?report=genbank&log$=prottop&blast_rank=3&RID=XE86KW0T016) | *Sitophilus zeamais* | 3e-54 | 61.0 |
| SvelOBP41 | 86 | N | PF01395 | - | 4 | [QKV34995.1](https://www.ncbi.nlm.nih.gov/protein/QKV34995.1?report=genbank&log$=prottop&blast_rank=2&RID=XE8D2TNG013) | *Dendroctonus adjunctus* | 6e-22 | 44.3 |
| SvelCSP1 | 141 | Y | PF03392 | 1-20 | 4 | [QFO46794.1](https://www.ncbi.nlm.nih.gov/protein/QFO46794.1?report=genbank&log$=prottop&blast_rank=1&RID=Y8FVGKX5013) | *Cylas formicarius* | 3e-67 | 76.6 |
| SvelCSP2 | 127 | Y | PF03392 | 1-18 | 4 | [NP_001039289.1](https://www.ncbi.nlm.nih.gov/protein/NP_001039289.1?report=genbank&log$=prottop&blast_rank=1&RID=NEF5NC9201R) | *Tribolium castaneum* | 1e-61 | 69.1 |
| SvelCSP3 | 132 | Y | PF03392 | 1-20 | 4 | [AHE13802.1](https://www.ncbi.nlm.nih.gov/protein/AHE13802.1?report=genbank&log$=prottop&blast_rank=3&RID=XGWG7DPU013) | *Lissorhoptrus oryzophilus* | 6e-59 | 69.5 |
| SvelCSP4 | 147 | Y | PF03392 | 1-26 | 4 | AKK25146.1 | *Dendroctonus ponderosae* | 6e-59 | 63.3 |
| SvelCSP5 | 130 | Y | PF03392 | 1-18 | 4 | [AVI04875.1](https://www.ncbi.nlm.nih.gov/protein/AVI04875.1?report=genbank&log$=prottop&blast_rank=1&RID=Y8GJXH6K016) | *Anthonomus grandis* | 4e-61 | 67.4 |
| SvelCSP6 | 367 | Y | PF03392 | 1-18 | 4 | [QFO46792.1](https://www.ncbi.nlm.nih.gov/protein/QFO46792.1?report=genbank&log$=prottop&blast_rank=5&RID=NEEEJHA2016) | *Cylas formicarius* | 2e-64 | 58.3 |
| SvelCSP7 | 130 | Y | PF03392 | 1-18 | 4 | [AVI04875.1](https://www.ncbi.nlm.nih.gov/protein/AVI04875.1?report=genbank&log$=prottop&blast_rank=1&RID=Y8GWUA37013) | *Anthonomus grandis* | 3e-56 | 66.7 |
| SvelCSP8 | 122 | Y | PF03392 | 1-18 | 4 | [AXF53965.1](https://www.ncbi.nlm.nih.gov/protein/AXF53965.1?report=genbank&log$=prottop&blast_rank=1&RID=NEES075U014) | *Dendroctonus armandi* | 4e-49 | 61.0 |
| SvelCSP9 | 128 | Y | PF03392 | 1-17 | 4 | AHE13803.1 | *Lissorhoptrus oryzophilus* | 5e-70 | 83.5 |
| SvelCSP10 | 131 | Y | PF03392 | - | 4 | [AHE13805.1](https://www.ncbi.nlm.nih.gov/protein/AHE13805.1?report=genbank&log$=prottop&blast_rank=2&RID=NEF0V57Z014) | *Lissorhoptrus oryzophilus* | 1e-48 | 57.3 |
| SvelCSP11 | 87 | N | PF03392 | 1-23 | 5 | [AUF72993.1](https://www.ncbi.nlm.nih.gov/protein/AUF72993.1?report=genbank&log$=prottop&blast_rank=3&RID=Y8HD5H3T016) | *Anoplophora chinensis* | 1e-20 | 67.2 |

^a^signal peptide; ^b^the number of conserved cystein.
